# Regioselective Reaction of 2-Indolylmethanols with Enamides

**DOI:** 10.3390/molecules28083341

**Published:** 2023-04-10

**Authors:** Yuting Tian, Dongqing He, Limei Gao, Yu Zou, Xiaoshuang Liu, Qiang Wang, Enxiang Liang, Yongsheng Zheng

**Affiliations:** 1 School of Pharmaceutical Sciences, South-Central Minzu University, Wuhan 430074, China; 15072425013@139.com (Y.T.); 13667157337@163.com (Y.Z.); m15025728534@163.com (X.L.); wq@mail.scuec.edu.cn (Q.W.); 2 Department of Chemistry and Chemical Engineering, Institute of Science and Technology, Yueyang 414000, China; m17671776102@163.com

**Keywords:** 2-indolylmethanols, enamides, regioselective, addition, hybrid

## Abstract

A highly regioselective reaction of 2-indolylmethanols with enamides has been developed at room temperature by using AlCl_3_ as a catalyst. A wide range of hybrids (40 examples) of indoles and enamides were obtained in moderate to good yields (up to 98% yield). This transformation represents the efficient way to introduce biologically important indoles and enamides skeleton into structurally complex hybrids.

## 1. Introduction

**Figure 1:**
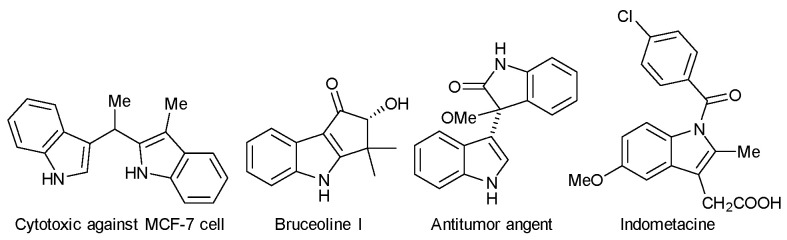
Representative drugs and biologically active compounds bearing indole unit.

Indole scaffold represents one of the most important heterocyclic frameworks, and it is found in the cores of many natural alkaloids, pharmaceuticals and functional materials (Figure 1) [1,2,3,4,5]. The synthesis of indole-containing derivatives has therefore remained an attractive research field. Among the established approaches, indolylmethanol-involved reactions have been proven to be straightforward methods for the synthesis of diverse indole derivatives through cycloadditions and substitution reactions [6,7,8,9,10,11,12,13,14,15,16,17,18,19]. In particular, 2-indolylmethanols that serve as three-carbon building blocks have been extensively explored to synthesize indole-fused scaffolds via [3 + 2] [20,21], [3 + 3] [22,23,24,25] and [4 + 3] [26,27,28] cyclizations. On the other hand, 2-indolylmethanols can also transform into carbocations, vinyliminium and delocalized cations in the presence of a Lewis or Brønsted acid to participate in various substitution reactions. In this context, various nucleophiles include indoles and electron-rich heterocycles [29,30,31,32], naphthol [33], phosphines [34,35], thiophenols [36], tryptamines and tryptophols [37], sodium sulfinates [38], aldehydes [39], vinyl silyl ethers [40], 2-alkyazaarenes [41], azlactones [42], prazol-5-ones [43], anhydrides and cyclic enaminoes [44,45] and guaiazulenes [46] and have been applied in the substitution of 2-indolylmethanols either at the C3 position or benzyl position. Nevertheless, the design and development of efficient, atom-economical and practical approaches are still needed to generate biologically important indole-containing compounds.

Nitrogen-containing heterocyclic compounds constitute important building blocks in biologically active compounds [47,48,49]. Enamides are versatile synthons in cycloadditions [50,51,52,53,54,55] and various functionalization reactions [56,57,58,59,60,61,62,63] to construct nitrogen-containing compounds. Because of our continual research interest in biologically important nitrogen-containing heterocycles synthesis and enamides chemistry [50,51,52,53,54,55], we envisioned that the hybrids of indoles and enamides would be obtained because of the nucleophilicity of enamides and the electrophilicity of 2-indolylmethanols under acidic conditions. Shi et al. reported the Brønsted acid-catalyzed reaction of 2-indolylmethanols with cyclic enaminones for the synthesis of 3-functionalized indole derivatives under elevated temperature [64,65]. Han developed the chiral phosphoramide-catalyzed asymmetric C2 alkylation of 2-indolylmethanols with acyclic enamides [65]. Herein, we report the highly regioselective C3 alkylation reaction of 2-indolylmethanols with cyclic enamides for the construction of hybrids of indoles and cyclic enamides under mild reaction conditions.

## 2. Results

Initially, the reaction of cyclic enamide **1a** and 2-indolylmethanol **2a** was employed as a model reaction to testify the feasibility of this reaction under the catalysis of a chiral phosphoric acid (CPA) at room temperature in toluene (Table 1, entry 1). The anticipated product could be obtained in 35% yield albeit with no atroposelectivity. The product was unambiguously determined by X-ray crystallographic analysis (CCDC 2224916). To improve the atroposelectivity of the reaction, a series of CPAs were screened. Unfortunately, further screening of CPAs did not give improved atroposelectivity for the reaction. Then, we turned to the racemic version of this transformation. No reaction occurred under other Brønsted acids, such as trifluoroacetic acid, AcOH, even at elevated temperatures (Table 1, entries 2–3). Therefore, the stronger acids were examined and found that TsOH catalyzed the C3 alkylation reaction smoothly and generated the product in 38% yield (Table 1, entry 4). Then, a variety of Lewis acids were screened to improve the reaction efficiency. However, Sc(OTf)_3_ and Cu(OTf)_2_ did not work at all. No desired product was detected, and the starting material was decomposed (Table 1, entries 5–6). Gratifyingly, AlCl_3_ gave the product in 40% yield within 2 h (Table 1, entry 7). AlCl_3_ was then selected as the optimal catalyst for further evaluation of the solvents (entries 8–12), which disclosed that DCM was most suitable in terms of yield and time, giving product **3a** in 88% yield within 2 h.

**Figure 2:**
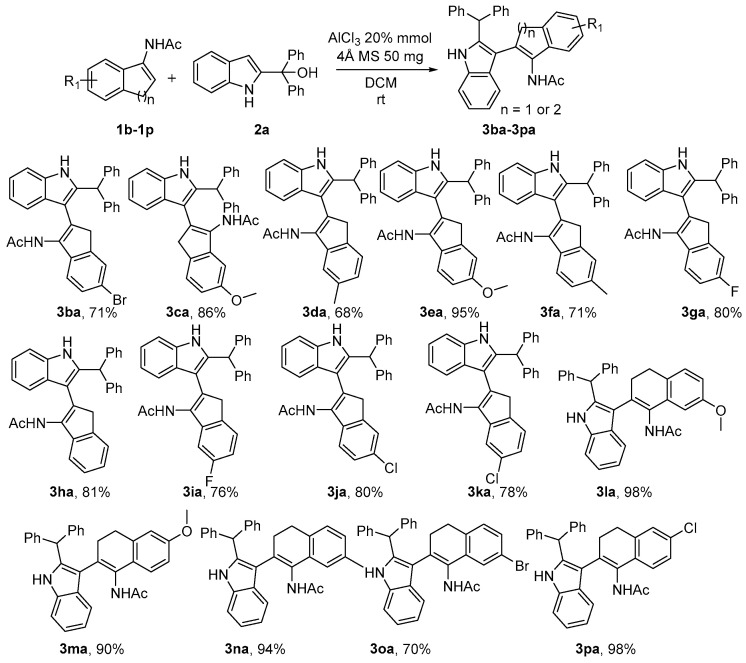
Scope of enamines.

Having established the optimal reaction conditions, we thereby examined the substrate scope of this protocol by employing a variety of enamides 1, and the results are summarized in Figure 2. Various cyclic enamides tested could smoothly furnish the corresponding C3 alkylation product in good to excellent yields (68–98%) under the optimal reaction conditions (Figure 2, **3ba**–**3pa**). The alkylation can be extended to a variety of five-membered (Figure 2, **3ba**–**3ka**) and six-membered enamides (Figure 2, **3la**–**3pa**) bearing either electron-rich (Figure 2, **3ca**–**3fa**, **3la**–**3na**) or electron-deficient (Figure 2, **3ga**–**3ka**, **3oa**–**3pa**) substituents on the phenyl ring, giving the corresponding C3 alkylation products in good yields. It is worth noting that when changing the acyl group with a benzoyl group on enamines, no reaction was observed probably due to the hindrance and the electron-deficient effect.

**Figure 3:**
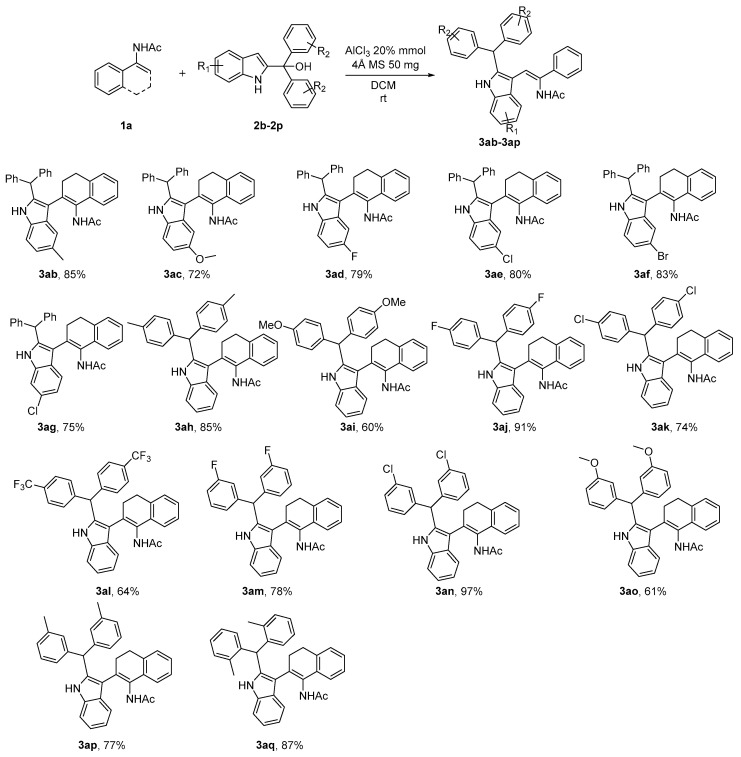
Scope of 2-indolemethanols.

To further examine the generality of the reaction, we next investigated the scope of different 2-indolylmethanols with six-membered enamides under the established conditions. First, the substituent effect on the indole ring was examined. The electronic effect on the indole ring seems to have no obvious effect on the reaction efficiency (Figure 3, **3ab** and **3ac** vs. **3ad**–**3af**). Then, 2-indolylmethanols derived from different Grignard reagents were applied in this reaction. The substrates with electron-donating groups such as methyl and methoxyl at the meta- and para-position of the benzene resulted in relatively lower yields compared to the electron-withdrawing ones (Figure 3, **3ah**, **3ai**, **3ao** and **3ap** vs. **3aj**, **3ak**, **3al**, **3am** and **3an**). These results showed that electron-donating substituents on the phenyl ring may reduce the electrophilicity of the in-site formed cation. It is worth noting that the ortho-position-substituted substrate was also compatible and afforded the **3aq** in 87% yield.

**Figure 4:**
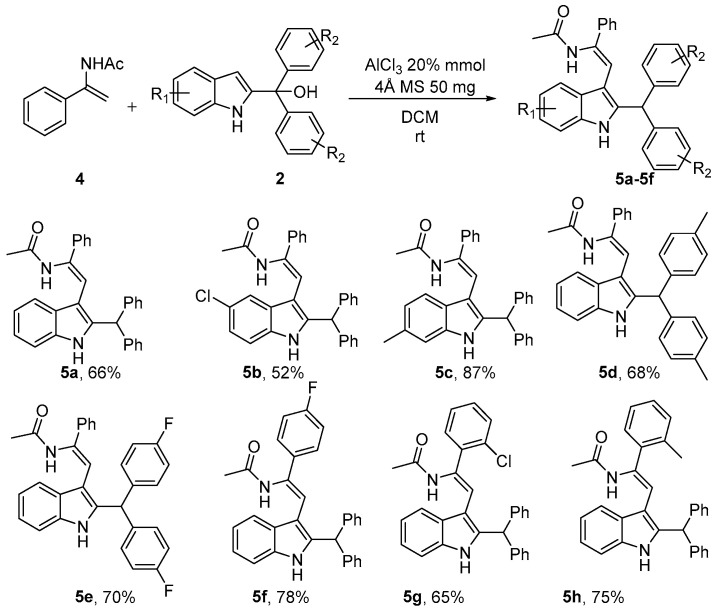
Scope of 2-indolemethanols with acyclic enamide 4.

To obtain the structure-diverse indole-containing compounds and further expand the scope of this reaction, acyclic enamides were used to couple with the 2-indolylmethanols. As shown in Figure 4, a series of highly functionalized enamides-containing indoles were obtained in 52–87% yield.

**Scheme 1:**
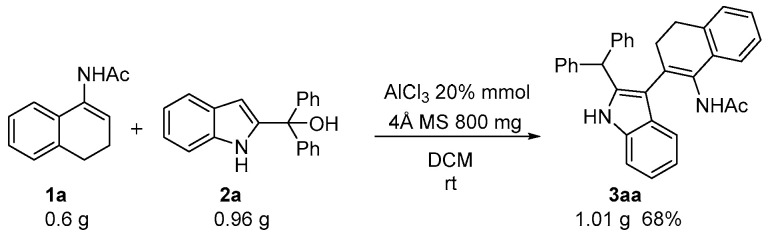
Gram-scale experiment for the synthesis of **3aa**.

To demonstrate the practical utility of this reaction, a gram-scale synthesis of **3aa** was performed with 3.2 mmol of **1a** with 3.2 mmol of **2a**, affording 1.01 g of **3aa** in 68% yield, without significant losses of yield compared with the small-scale reaction (Scheme 1).

**Scheme 2:**
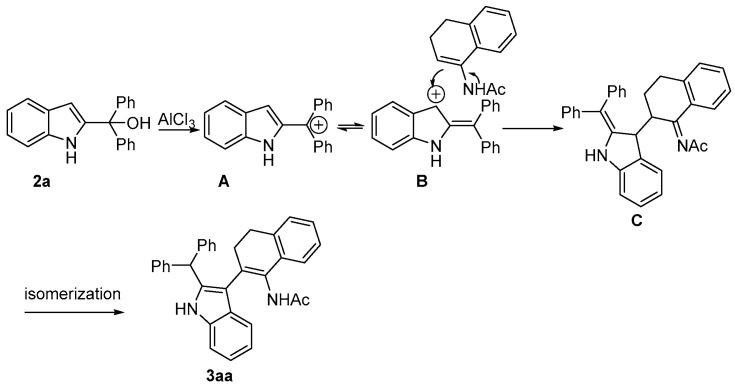
Proposed mechanism.

Based on the literature information [6,7,8,9], a plausible mechanism was proposed. As depicted in Scheme 2. Cations A and B were generated in the presence of Lewis acid. Next, enamide attacked the C3 position of the indole unit. The subsequent isomerization processes of the imine and indole gave the final product **3aa**.

## 3. Experimental Section

### 3.1. General Procedures

Unless otherwise specified, all reactions were carried out under nitrogen atmosphere in anhydrous conditions. All chemicals that are commercially available were used without further purification unless otherwise noted. All the solvents were purified according to the standard procedures. Analytical thin-layer chromatography (TLC) was performed on silica gel plates (GF-254) using UV light (254 and 365 nm). Flash chromatography was conducted on silica gel (200–300 mesh). NMR spectra were recorded at ambient temperature in CDCl3 and DMSO on a Bruker AMX 500 (1H NMR at 500 MHz and 13C NMR at 125 MHz) or on an AVANCE III (1H NMR at 400 MHz and 13C NMR at 100 MHz) spectrometer. Chemical shifts were reported in parts per million (ppm) downfield from an internal standard, tetramethylsilane (0 ppm). High-resolution mass spectra were obtained on an Agilent 6200 Q-TOF MS and Waters G2-S QTOF.

### 3.2. Typical Procedure for Synthesis of ***3aa***

To a dried test tube with a magnetic stirring bar under N_2_ atmosphere at room temperature, **1a** (37.4 mg, 0.2 mmol, 1.00 eq.) and **2a** (93.6 mg, 0.2 mmol, 1.00 eq.) were added, followed by the addition of AlCl_3_ (0.04 mmol, 5.4 mg) and 4Å MS (50 mg). Then DCM (2.0 mL) was introduced by a syringe and the mixture was stirred at room temperature for 2 h. After completion of TLC analysis, the solvent was removed by rotary evaporation, and the residue was purified by column chromatography on silica gel (petroleum ether/ethyl acetate = 4:1) to afford the product **3aa** as a green solid.

*2-(2-benzhydryl-1H-indol-3-yl)-3,4-dihydronaphthalen-1-amine* (**3aa**). The product **3aa** was obtained by column chromatography on silica gel (eluent: petroleum ether/ethyl acetate = 4:1) as a green solid (82.4 mg, yield: 88%); mp 289–290 °C; ^1^H NMR (500 MHz, CDCl_3_) *δ* 7.92 (*s*, 1H), 7.41 (*d*, *J* = 7.8 Hz, 1H), 7.35 (*t*, *J* = 7.4 Hz, 2H), 7.31–7.21 (*m*, 8H), 7.19–7.14 (*m*, 3H), 7.13–7.03 (*m*, 4H), 6.37 (*s*, 1H), 5.71 (*s*, 1H), 2.93–2.82 (*m*, 1H), 2.77–2.67 (*m*, 1H), 2.66–2.56 (*m*, 1H), 2.42–2.32 (*m*, 1H), 1.61 (*s*, 3H); ^13^C NMR (125 MHz, CDCl_3_) *δ* 168.9, 142.4, 141.6, 136.5, 136.0, 135.8, 131.8, 131.6, 129.2, 129.1, 128.8, 127.5, 127.3, 127.2, 127.2, 127.0, 126.9, 126.5, 126.3, 123.5, 122.2, 120.4, 119.6, 113.0, 111.2, 48.7, 29.7, 28.2, 23.3; HRMS (ESI) *m*/*z*: [M + H]^+^ calculated for C_33_H_29_ON_2_, 469.2274; found, 469.2275.
*N-(2-(2-benzhydryl-1H-indol-3-yl)-1H-inden-3-yl)acetamide* (**3ba**). The product **3ba** was obtained by column chromatography on silica gel (eluent: petroleum ether/ethyl acetate = 2:1) as a white solid (73.6 mg, yield: 81%); mp 306–307 °C; ^1^H NMR (500 MHz, DMSO-d6) *δ* 11.04 (*s*, 1H), 9.34 (*s*, 1H), 7.49 (*d*, *J* = 7.9 Hz, 1H), 7.43 (*d*, *J* = 7.3 Hz, 1H), 7.36 (*d*, *J* = 8.1 Hz, 1H), 7.31 (*t*, *J* = 7.5 Hz, 4H), 7.25–7.20 (*m*, 3H), 7.20–7.14 (*m*, 6H), 7.10–7.04 (*m*, 1H), 7.00–6.95 (*m*, 1H), 5.72 (*s*, 1H), 3.72 (*s*, 2H), 1.48 (*s*, 3H); ^13^C NMR (125 MHz, DMSO-d6) *δ* 169.0, 142.8, 142.6, 141.6, 137.8, 136.7, 134.3, 130.0, 128.9, 128.3, 127.2, 126.4, 125.7, 124.1, 123.4, 121.1, 120.3, 119.2, 119.0, 111.4, 108.3, 47.8, 40.1, 22.0; HRMS (ESI) *m*/*z*: [M + H]^+^ calculated for C_32_H_26_BrON_2_, 533.1223; found, 533.1224.
*N-(2-(2-benzhydryl-1H-indol-3-yl)-5-methoxy-1H-inden-3-yl)acetamide* (**3ca**). The product **3ca** was obtained by column chromatography on silica gel (eluent: petroleum ether/ethyl acetate = 3:1) as a gray solid (83.4 mg, yield: 86%); mp 314–315 °C; ^1^H NMR (500 MHz, CDCl_3_) *δ* 8.05 (*s*, 1H), 7.49 (*d*, *J* = 7.8 Hz, 1H), 7.35–7.26 (*m*, 8H), 7.20 (*t*, *J* = 7.5 Hz, 1H), 7.18–7.10 (*m*, 5H), 7.02 (*d*, *J* = 2.4 Hz, 1H), 6.82 (*dd*, *J* = 8.2, 2.3 Hz, 1H), 6.66 (*s*, 1H), 5.74 (*s*, 1H), 3.84 (*s*, 3H), 3.64 (*s*, 2H), 1.57 (*s*, 3H); ^13^C NMR (125 MHz, CDCl_3_) *δ* 169.1, 158.9, 142.8, 142.2, 137.4, 136.1, 135.6, 134.6, 129.3, 129.0, 128.9, 128.0, 127.2, 124.0, 122.4, 120.5, 119.8, 112.0, 111.3, 108.6, 106.8, 55.8, 48.5, 40.0, 23.0; HRMS (ESI) *m*/*z*: [M + H]^+^ calculated for C_33_H_29_O_2_N_2_, 485.2224; found, 485.2223.
*N-(2-(2-benzhydryl-1H-indol-3-yl)-5-methyl-1H-inden-3-yl)acetamide* (**3da**). The product **3da** was obtained by column chromatography on silica gel (eluent: petroleum ether/ethyl acetate = 3:1) as a white solid (63.7 mg, yield: 68%); mp 306–307 °C; ^1^H NMR (600 MHz, DMSO-*d*_6_) *δ* 11.04 (*s*, 1H), 9.32 (*s*, 1H), 7.49 (*d*, *J* = 8.0 Hz, 1H), 7.37 (*d*, *J* = 8.1 Hz, 1H), 7.34–7.28 (*m*, 5H), 7.23 (*t*, *J* = 7.3 Hz, 2H), 7.19 (*d*, *J* = 7.5 Hz, 4H), 7.10–7.05 (*m*, 1H), 7.01–6.96 (*m*, 3H), 5.73 (*s*, 1H), 3.66 (*s*, 2H), 2.35 (*s*, 3H), 1.51 (*s*, 3H); ^13^C NMR (150 MHz, DMSO-*d*_6_) *δ* 168.9, 143.0, 142.6, 138.7, 137.7, 136.6, 134.6, 134.2, 130.4, 128.9, 128.3, 127.3, 126.4, 124.9, 123.1, 121.1, 120.7, 119.2, 119.0, 111.4, 108.4, 47.8, 22.1, 21.3; HRMS (ESI) *m*/*z*: [M + H]^+^ calculated for C_33_H_29_ON_2_, 469.2274; found, 469.2275.
*N-(2-(2-benzhydryl-1H-indol-3-yl)-6-methoxy-1H-inden-3-yl)acetamide* (**3ea**). The product **3ea** was obtained by column chromatography on silica gel (eluent: petroleum ether/ethyl acetate = 3:1) as a brown solid (92.1 mg, yield: 95%); mp 306–307 °C; ^1^H NMR (500 MHz, CDCl_3_) *δ* 8.01 (*s*, 1H), 7.48 (*d*, *J* = 7.9 Hz, 1H), 7.36 (*d*, *J* = 8.5 Hz, 1H), 7.32 (*t*, *J* = 7.4 Hz, 5H), 7.27 (*d*, *J* = 7.3 Hz, 2H), 7.19 (*t*, *J* = 7.4 Hz, 1H), 7.17–7.09 (*m*, 5H), 7.01 (*d*, *J* = 1.8 Hz, 1H), 6.88 (*dd*, *J* = 8.4, 2.1 Hz, 1H), 6.66 (*s*, 1H), 5.74 (*s*, 1H), 3.84 (*s*, 3H), 3.65 (*s*, 2H), 1.57 (*s*, 3H); ^13^C NMR (125 MHz, CDCl_3_) *δ* 169.0, 158.2, 144.3, 142.3, 137.3, 136.1, 135.4, 134.5, 129.0, 128.9, 128.1, 127.2, 125.0, 122.4, 122.3, 120.5, 119.8, 112.2, 111.2, 110.0, 108.6, 55.8, 48.5, 40.6, 23.0; HRMS (ESI) *m*/*z*: [M + H]^+^ calculated for C_33_H_29_O_2_N_2_, 485.2224; found, 485.2223.
*N-(2-(2-benzhydryl-1H-indol-3-yl)-6-methyl-1H-inden-3-yl)acetamide* (**3fa**). The product **3fa** was obtained by column chromatography on silica gel (eluent: petroleum ether/ethyl acetate = 3:1) as a brown solid (66.2 mg, yield: 71%); mp 296–297 °C; ^1^H NMR (400 MHz, CDCl_3_) *δ* 7.97 (*s*, 1H), 7.48 (*d*, *J* = 7.9 Hz, 1H), 7.35–7.27 (*m*, 8H), 7.24 (*s*, 1H), 7.19 (*t*, *J* = 7.5 Hz, 1H), 7.16–7.10 (*m*, 6H), 6.64 (*s*, 1H), 5.73 (*s*, 1H), 3.65 (*s*, 2H), 2.41 (*s*, 3H), 1.58 (*s*, 3H); ^13^C NMR (100 MHz, CDCl_3_) *δ* 169.0, 142.7, 142.3, 138.8, 137.4, 136.1, 135.7, 134.9, 129.1, 128.9, 128.1, 127.2, 127.2, 126.4, 124.5, 122.4, 121.4, 120.5, 119.9, 111.2, 108.7, 48.5, 40.5, 23.1, 21.6; HRMS (ESI) *m*/*z*: [M + H]^+^ calculated for C_33_H_29_ON_2_, 469.2274; found, 469.2273.
*N-(2-(2-benzhydryl-1H-indol-3-yl)-6-fluoro-1H-inden-3-yl)acetamide* (**3ga**). The product **3ga** was obtained by column chromatography on silica gel (eluent: petroleum ether/ethyl acetate = 3:1) as a white solid (75.4 mg, yield: 80%); mp 356–357 °C; ^1^H NMR (600 MHz, DMSO-*d*_6_) δ 11.06 (*s*, 1H), 9.38 (*s*, 1H), 7.50 (*dd*, *J* = 7.7, 3.0 Hz, 1H), 7.38 (*dd*, *J* = 7.7, 4.7 Hz, 1H), 7.34–7.28 (*m*, 5H), 7.26–7.18 (*m*, 6H), 7.17–7.13 (*m*, 1H), 7.12–7.06 (*m*, 2H), 7.02–6.96 (*m*, 1H), 5.72 (*s*, 1H), 3.75 (*s*, 2H), 1.50 (*s*, 3H); ^13^C NMR (150 MHz, DMSO-*d*_6_) δ 169.0, 161.2, 159.6, 143.9 (*d*, *J* = 8.8 Hz), 142.6, 139.0, 137.8, 136.7, 133.6, 129.7 (*d*, *J* = 3.5 Hz), 128.9, 128.3, 127.2, 126.4, 121.2, 121.2, 119.1 (*d*, *J* = 17.0 Hz), 112.5 (*d*, *J* = 22.6 Hz), 111.4, 110.9 (*d*, *J* = 23.0 Hz), 108.1, 47.8, 40.1, 22.0; ^19^F NMR (471 MHz, DMSO-*d*_6_) *δ* −119.15–−119.20 (*m*, 1F); HRMS (ESI) *m*/*z*: [M + H]^+^ calculated for C_32_H_26_ON_2_F, 473.2024; found, 473.2024.
*N-(2-(2-benzhydryl-1H-indol-3-yl)-6-chloro-1H-inden-3-yl)acetamide* (**3ha**). The product **3ha** was obtained by column chromatography on silica gel (eluent: petroleum ether/ethyl acetate = 3:1) as a white solid (78.2 mg, yield: 80%); mp 334–335 °C; ^1^H NMR (500 MHz, DMSO-*d*_6_) *δ* 11.09 (*s*, 1H), 9.41 (*s*, 1H), 7.51–7.46 (*m*, 2H), 7.37 (*d*, *J* = 8.1 Hz, 1H), 7.34–7.28 (*m*, 5H), 7.25–7.21 (*m*, 2H), 7.18 (*d*, *J* = 7.4 Hz, 4H), 7.14 (*d*, *J* = 8.2 Hz, 1H), 7.11–7.05 (*m*, 1H), 7.02–6.96 (*m*, 1H), 5.70 (*s*, 1H), 3.77 (*s*, 2H), 1.48 (*s*, 3H); ^13^C NMR (125 MHz, DMSO-*d*_6_) *δ* 169.1, 143.8, 142.5, 141.7, 138.0, 136.7, 133.6, 130.8, 129.0, 128.9, 128.4, 127.1, 126.4, 125.8, 123.6, 121.6, 121.2, 119.2, 119.2, 111.5, 108.0, 47.9, 40.0, 22.0; HRMS (ESI) *m*/*z*: [M + H]^+^ calculated for C_32_H_27_ON_2_, 455.2118; found, 455.2116.
*N-(2-(2-benzhydryl-1H-indol-3-yl)-6-bromo-1H-inden-3-yl)acetamide* (**3ia**). The product **3ia** was obtained by column chromatography on silica gel (eluent: petroleum ether/ethyl acetate = 3:1) as a yellow solid (75.2 mg, yield: 71%); mp 296–297 °C; ^1^H NMR (600 MHz, DMSO-*d*_6_) *δ* 11.09 (*s*, 1H), 9.41 (*s*, 1H), 7.63 (*s*, 1H), 7.49 (*dd*, *J* = 7.9, 2.3 Hz, 1H), 7.45 (*d*, *J* = 8.1 Hz, 1H), 7.38 (*dd*, *J* = 7.8, 4.0 Hz, 1H), 7.31 (*t*, *J* = 7.4 Hz, 4H), 7.23 (*t*, *J* = 7.3 Hz, 2H), 7.20–7.17 (*m*, 4H), 7.12–7.06 (*m*, 2H), 7.03–6.92 (*m*, 1H), 5.71 (*s*, 1H), 3.76 (*s*, 2H), 1.50 (*s*, 3H); ^13^C NMR (150 MHz, DMSO-*d*_6_) *δ* 169.1, 144.1, 142.5, 142.0, 138.0, 136.7, 133.6, 130.8, 128.9, 128.5, 128.3, 127.1, 126.4, 126.4, 122.0, 121.2, 119.2, 117.4, 111.5, 107.9, 47.8, 40.0, 22.0; HRMS (ESI) *m*/*z*: [M + H]^+^ calculated for C_32_H_26_ON_2_F, 473.2024; found, 473.2023.
*N-(2-(2-benzhydryl-1H-indol-3-yl)-5-fluoro-1H-inden-3-yl)acetamide* (**3ja**). The product **3ja** was obtained by column chromatography on silica gel (eluent: petroleum ether/ethyl acetate = 3:1) as a brown solid (71.2 mg, yield: 76%); mp 331–332 °C; ^1^H NMR (600 MHz, DMSO-*d*_6_) *δ* 11.08 (*s*, 1H), 9.40 (*s*, 1H), 7.49 (*d*, *J* = 8.0 Hz, 1H), 7.42 (*dd*, *J* = 8.1, 5.2 Hz, 1H), 7.37 (*d*, *J* = 8.1 Hz, 1H), 7.31 (*t*, *J* = 7.6 Hz, 4H), 7.23 (*t*, *J* = 7.3 Hz, 2H), 7.19 (*d*, *J* = 7.5 Hz, 4H), 7.10–7.05 (*m*, 1H), 7.01–6.95 (*m*, 2H), 6.91 (*dd*, *J* = 9.5, 2.4 Hz, 1H), 5.71 (*s*, 1H), 3.72 (*s*, 2H), 1.50 (*s*, 3H); ^13^C NMR (150 MHz, DMSO-*d*_6_) *δ* 169.1, 162.2, 160.6, 144.9 (*d*, *J* = 9.4 Hz), 142.5, 137.9, 137.3 (*d*, *J* = 1.5 Hz), 136.6, 133.7 (*d*, *J* = 2.9 Hz), 132.6, 128.9, 128.3, 127.1, 126.4, 124.4 (*d*, *J* = 9.0 Hz), 121.2, 119.2 (*d*, *J* = 8.5 Hz), 111.4, 110.6 (*d*, *J* = 22.8 Hz), 108.0, 107.1 (*d*, *J* = 23.9 Hz), 47.8, 40.1, 22.0; ^19^F NMR (471 MHz, DMSO-*d*_6_) *δ* −117.67–−117.72 (*m*, 1F); HRMS (ESI) *m*/*z*: [M + H]^+^ calculated for C_32_H_26_ON_2_Cl, 489.1728; found, 489.1727.
*N-(2-(2-benzhydryl-1H-indol-3-yl)-5-chloro-1H-inden-3-yl)acetamide* (**3ka**). The product **3ka** was obtained by column chromatography on silica gel (eluent: petroleum ether/ethyl acetate = 3:1) as a white solid (75.9 mg, yield: 78%); mp 327–328 °C; ^1^H NMR (500 MHz, DMSO-*d*_6_) *δ* 11.11 (*s*, 1H), 9.45 (*s*, 1H), 7.50 (*d*, *J* = 8.0 Hz, 1H), 7.44 (*d*, *J* = 8.0 Hz, 1H), 7.39 (*d*, *J* = 8.1 Hz, 1H), 7.33–7.29 (*m*, 4H), 7.25–7.18 (*m*, 7H), 7.17 (*d*, *J* = 2.0 Hz, 1H), 7.12–7.07 (*m*, 1H), 7.03–6.97 (*m*, 1H), 5.72 (*s*, 1H), 3.76 (*s*, 2H), 1.52 (*s*, 3H); ^13^C NMR (125 MHz, DMSO-*d*_6_) *δ* 169.3, 144.8, 142.5, 140.4, 138.1, 136.7, 133.3, 132.3, 130.8, 128.9, 128.4, 127.1, 126.5, 124.8, 123.8, 121.3, 120.1, 119.2, 111.5, 108.0, 47.9, 39.8, 22.0; HRMS (ESI) *m*/*z*: [M + H]^+^ calculated for C_32_H_26_ON_2_Cl, 489.1728; found, 489.1727.
*N-(2-(2-benzhydryl-1H-indol-3-yl)-7-methoxy-3,4-dihydronaphthalen-1-yl)acetamide* (**3la**). The product **3la** was obtained by column chromatography on silica gel (eluent: petroleum ether/ethyl acetate = 3:1) as a white solid (97.5 mg, yield: 98%); mp 324–325 °C; ^1^H NMR (500 MHz, DMSO-*d*_6_) *δ* 10.91 (*s*, 1H), 8.93 (*s*, 1H), 7.38–7.27 (*m*, 6H), 7.29–7.22 (*m*, 3H), 7.22–7.18 (*m*, 3H), 7.11 (*d*, *J* = 8.2 Hz, 1H), 7.08–7.02 (*m*, 1H), 6.97–6.92 (*m*, 1H), 6.74 (*dd*, *J* = 8.2, 2.6 Hz, 1H), 6.58 (*d*, *J* = 2.6 Hz, 1H), 5.72 (*s*, 1H), 3.71 (*s*, 3H), 2.78–2.57 (*m*, 3H), 2.23–2.15 (*m*, 1H), 1.46 (*s*, 3H); ^13^C NMR (125 MHz, DMSO-*d*_6_) *δ* 168.8, 157.9, 143.5, 142.1, 136.8, 136.6, 134.2, 129.9, 129.1, 128.8, 128.7, 128.5, 128.1, 127.9, 127.7, 126.4, 126.3, 126.3, 120.8, 119.5, 118.7, 112.9, 111.4, 111.0, 109.6, 55.0, 47.8, 30.2, 26.9, 22.2; HRMS (ESI) *m*/*z*: [M + H]^+^ calculated for C_34_H_31_O_2_N_2_F, 499.2380; found, 499.2376.
*N-(2-(2-benzhydryl-1H-indol-3-yl)-6-methoxy-3,4-dihydronaphthalen-1-yl)acetamide* (**3ma**). The product **3ma** was obtained by column chromatography on silica gel (eluent: petroleum ether/ethyl acetate = 3:1) as a brown solid (89.7 mg, yield: 90%); mp 321–322 °C; ^1^H NMR (500 MHz, DMSO-*d*_6_) *δ* 10.86 (*s*, 1H), 8.87 (*s*, 1H), 7.37–7.23 (*m*, 9H), 7.20 (*t*, *J* = 8.7 Hz, 3H), 7.04 (*t*, *J* = 7.6 Hz, 1H), 6.97–6.91 (*m*, 2H), 6.81 (*d*, *J* = 2.3 Hz, 1H), 6.73 (*dd*, *J* = 8.5, 2.5 Hz, 1H), 5.72 (*s*, 1H), 3.76 (*s*, 3H), 2.76–2.67 (*m*, 3H), 2.23–2.11 (*m*, 1H), 1.44 (*s*, 3H); ^13^C NMR (125 MHz, DMSO-*d*_6_) *δ* 168.8, 158.0, 143.6, 142.2, 137.4, 136.6, 136.5, 129.7, 129.1, 128.8, 128.5, 128.1, 126.5, 126.4, 126.3, 126.0, 125.1, 124.5, 120.8, 119.5, 118.6, 113.0, 112.9, 111.4, 111.1, 55.1, 47.8, 29.7, 28.2, 22.2; HRMS (ESI) *m*/*z*: [M + H]^+^ calculated for C_34_H_31_O_2_N_2_, 499.2380; found, 499.2377.
*N-(2-(2-benzhydryl-1H-indol-3-yl)-7-methyl-3,4-dihydronaphthalen-1-yl)acetamide* (**3na**). The product **3na** was obtained by column chromatography on silica gel (eluent: petroleum ether/ethyl acetate = 3:1) as a black solid (90.5 mg, yield: 94%); mp 316–317 °C; ^1^H NMR (500 MHz, DMSO-*d*_6_) *δ* 10.88 (*s*, 1H), 8.91 (*s*, 1H), 7.36–7.31 (*m*, 4H), 7.32–7.26 (*m*, 2H), 7.27–7.22 (*m*, 3H), 7.22–7.17 (*m*, 3H), 7.10–7.01 (*m*, 2H), 6.98–6.91 (*m*, 2H), 6.84 (*s*, 1H), 5.72 (*s*, 1H), 2.72–2.64 (*m*, 3H), 2.25 (*s*, 3H), 2.21–2.12 (*m*, 1H), 1.47 (*s*, 3H); ^13^C NMR (125 MHz, DMSO-*d*_6_) *δ* 168.8, 143.5, 142.1, 136.7, 136.6, 134.7, 133.0, 132.7, 130.0, 129.1, 128.8, 128.5, 128.1, 128.1, 127.0, 126.9, 126.4, 126.4, 126.3, 123.6, 120.8, 119.5, 118.7, 112.9, 111.4, 47.8, 30.0, 27.4, 22.2, 21.1; HRMS (ESI) *m*/*z*: [M + H]^+^ calculated for C_34_H_31_ON_2_, 483.2431; found, 483.2425.
*N-(2-(2-benzhydryl-1H-indol-3-yl)-7-bromo-3,4-dihydronaphthalen-1-yl)acetamide* (**3oa**). The product **3oa** was obtained by column chromatography on silica gel (eluent: petroleum ether/ethyl acetate = 3:1) as a yellow solid (76.5 mg, yield: 70%); mp 301–302 °C; ^1^H NMR (500 MHz, DMSO-*d*_6_) *δ* 10.95 (s, 1H), 9.03 (*s*, 1H), 7.38–7.31 (*m*, 5H), 7.29 (*d*, *J* = 7.4 Hz, 2H), 7.25 (*t*, *J* = 8.0 Hz, 3H), 7.20 (*t*, *J* = 7.3 Hz, 3H), 7.16 (*d*, *J* = 8.0 Hz, 1H), 7.10 (*d*, *J* = 2.0 Hz, 1H), 7.09–7.03 (*m*, 1H), 6.98–6.93 (*m*, 1H), 5.68 (*s*, 1H), 2.78–2.60 (*m*, 3H), 2.27–2.17 (*m*, 1H), 1.46 (*s*, 3H); ^13^C NMR (125 MHz, DMSO-*d*_6_) *δ* 169.0, 143.4, 142.0, 136.9, 136.6, 135.4, 134.9, 130.0, 129.2, 129.1, 128.8, 128.8, 128.5, 128.2, 126.5, 126.4, 126.2, 125.4, 121.0, 119.5, 119.2, 118.8, 112.4, 111.5, 47.9, 29.5, 27.1, 22.1; HRMS (ESI) *m*/*z*: [M + H]^+^ calculated for C_33_H_28_ON_2_Br, 547.1380; found, 547.1379.
*N-(2-(2-benzhydryl-1H-indol-3-yl)-6-chloro-3,4-dihydronaphthalen-1-yl)acetamide* (**3pa**). The product **3pa** was obtained by column chromatography on silica gel (eluent: petroleum ether/ethyl acetate = 3:1) as a yellow solid (98.6 mg, yield: 98%); mp 301–302 °C. ^1^H NMR (500 MHz, DMSO-*d*_6_) *δ* 10.92 (s, 1H), 9.00 (s, 1H), 7.36–7.31 (m, 4H), 7.31–7.25 (m, 3H), 7.24 (d, *J* = 10.5 Hz, 2H), 7.24–7.15 (m, 5H), 7.08–7.01 (m, 1H), 6.98 (d, *J* = 8.3 Hz, 1H), 6.97–6.91 (m, 1H), 5.67 (s, 1H), 2.79–2.68 (m, 3H), 2.24–2.15 (m, 1H), 1.43 (s, 3H); ^13^C NMR (125 MHz, DMSO-*d*_6_) *δ* 169.0, 143.5, 142.0, 138.0, 136.9, 136.6, 131.9, 130.5, 129.2, 129.1, 128.8, 128.6, 128.5, 128.2, 126.8, 126.5, 126.3, 126.3, 125.8, 124.9, 120.9, 119.5, 118.8, 112.5, 111.5, 47.9, 29.4, 27.4, 22.1; HRMS (ESI) *m*/*z*: [M + H]^+^ calculated for C_33_H_28_ON_2_Cl, 503.1885; found, 503.1883.
*N-(2-(2-benzhydryl-5-methyl-1H-indol-3-yl)-3,4-dihydronaphthalen-1-yl)acetamide* (**3ab**). The product **3ab** was obtained by column chromatography on silica gel (eluent: petroleum ether/ethyl acetate = 3:1) as a yellow solid (81.9 mg, yield: 85%); mp 303–304 °C; ^1^H NMR (500 MHz, DMSO-*d*_6_) *δ* 10.73 (*s*, 1H), 8.88 (*s*, 1H), 7.34–7.27 (*m*, 4H), 7.24–7.18 (*m*, 5H), 7.18–7.10 (*m*, 6H), 7.00 (*dd*, *J* = 7.2, 1.6 Hz, 1H), 6.86 (*dd*, *J* = 8.3, 1.2 Hz, 1H), 5.68 (*s*, 1H), 2.76–2.68 (*m*, 3H), 2.31 (*s*, 3H), 2.23–2.12 (*m*, 1H), 1.45 (*s*, 3H); ^13^C NMR (125 MHz, DMSO-*d*_6_) *δ* 168.8, 143.6, 142.2, 136.7, 135.6, 134.9, 133.0, 129.9, 129.1, 128.8, 128.4, 128.1, 128.1, 127.1, 127.0, 126.5, 126.4, 126.3, 126.2, 126.0, 123.0, 122.2, 119.2, 112.3, 111.1, 47.8, 29.7, 27.7, 22.1, 21.2; HRMS (ESI) *m*/*z*: [M + H]^+^ calculated for C_34_H_31_ON_2_, 483.2431; found, 483.2430.
*N-(2-(2-benzhydryl-5-methoxy-1H-indol-3-yl)-3,4-dihydronaphthalen-1-yl)acetamide* (**3ac**). The product **3ac** was obtained by column chromatography on silica gel (eluent: petroleum ether/ethyl acetate = 5:1) as a yellow solid (71.2 mg, yield: 72%); mp 313–314 °C; ^1^H NMR (500 MHz, DMSO-*d*_6_) *δ* 10.70 (*s*, 1H), 8.90 (*s*, 1H), 7.31 (q, *J* = 7.8 Hz, 4H), 7.25–7.20 (*m*, 4H), 7.18 (*d*, *J* = 7.9 Hz, 4H), 7.16–7.12 (*m*, 2H), 7.03–7.00 (*m*, 1H), 6.80 (*d*, *J* = 2.4 Hz, 1H), 6.68 (*dd*, *J* = 8.7, 2.4 Hz, 1H), 5.69 (*s*, 1H), 3.69 (*s*, 3H), 2.92–2.80 (*m*, 1H), 2.77–2.63 (*m*, 3H), 1.52 (*s*, 3H); ^13^C NMR (125 MHz, DMSO-*d*_6_) *δ* 169.0, 153.3, 143.4, 142.3, 137.4, 135.7, 133.1, 131.5, 129.9, 129.1, 128.8, 128.5, 128.2, 127.9, 127.1, 126.6, 126.5, 126.4, 126.3, 126.0, 123.1, 112.8, 112.1, 111.0, 101.3, 55.4, 48.0, 29.6, 27.8, 22.4; HRMS (ESI) *m*/*z*: [M + H]^+^ calculated for C_34_H_31_O_2_N_2_, 499.2380; found, 499.2378.
*N-(2-(2-benzhydryl-5-fluoro-1H-indol-3-yl)-3,4-dihydronaphthalen-1-yl)acetamide* (**3ad**). The product **3ad** was obtained by column chromatography on silica gel (eluent: petroleum ether/ethyl acetate = 5:1) as a yellow solid (76.7 mg, yield: 79%); mp 313–314 °C; ^1^H NMR (500 MHz, DMSO-*d*_6_) *δ* 10.96 (*s*, 1H), 8.96 (*s*, 1H), 7.37–7.29 (*m*, 5H), 7.28–7.13 (*m*, 9H), 7.09–7.01 (*m*, 2H), 6.93–6.84 (*m*, 1H), 5.72 (*s*, 1H), 2.78–2.57 (*m*, 3H), 2.25–2.06 (*m*, 1H), 1.52 (*s*, 3H); ^13^C NMR (125 MHz, DMSO-*d*_6_) *δ* 168.8, 157.8, 156.0, 143.1, 142.0, 138.9, 135.7, 133.0 (*d*, *J* = 13.3 Hz), 130.2, 129.1, 128.8, 128.5, 128.2, 127.5, 127.1, 126.6, 126.5, 126.5, 126.4, 126.0, 123.1, 113.2 (*d*, *J* = 4.4 Hz), 112.2 (*d*, *J* = 9.6 Hz), 108.7 (*d*, *J* = 25.8 Hz), 104.2 (*d*, *J* = 23.4 Hz), 47.9, 29.4, 27.7, 22.2; ^19^F NMR (471 MHz, CDCl_3_) *δ* −124.89–−124.94 (*m*, 1F); HRMS (ESI) *m*/*z*: [M + H]^+^ calculated for C_33_H_28_ON_2_F, 487.2180; found, 487.2177.
*N-(2-(2-benzhydryl-5-chloro-1H-indol-3-yl)-3,4-dihydronaphthalen-1-yl)acetamide* (**3ae**). The product **3ae** was obtained by column chromatography on silica gel (eluent: petroleum ether/ethyl acetate = 5:1) as a yellow solid (79.8 mg, yield: 80%); mp 314–315 °C; ^1^H NMR (500 MHz, DMSO-*d*_6_) *δ* 11.07 (s, 1H), 8.97 (*s*, 1H), 7.40–7.29 (*m*, 6H), 7.28–7.21 (*m*, 4H), 7.20–7.12 (*m*, 5H), 5.74 (*s*, 1H), 2.81–2.58 (*m*, 3H), 2.28–2.12 (*m*, 1H), 1.55 (*s*, 3H); ^13^C NMR (125 MHz, DMSO-*d*_6_) *δ* 168.8, 143.0, 141.9, 138.6, 135.7, 134.9, 132.9, 130.5, 129.1, 128.8, 128.5, 128.2, 127.4, 127.2, 127.1, 126.6, 126.4, 126.0, 123.4, 123.1, 120.7, 118.6, 112.9, 112.8, 47.9, 29.5, 27.7, 22.3; HRMS (ESI) *m*/*z*: [M + H]^+^ calculated for C_33_H_28_ON_2_Cl, 503.1885; found, 503.1886.
*N-(2-(2-benzhydryl-5-bromo-1H-indol-3-yl)-3,4-dihydronaphthalen-1-yl)acetamide* (**3af**). The product **3af** was obtained by column chromatography on silica gel (eluent: petroleum ether/ethyl acetate = 5:1) as a yellow solid (90.5 mg, yield: 83%); mp 326–327 °C; ^1^H NMR (500 MHz, DMSO-*d*_6_) *δ* 11.06 (s, 1H), 8.95 (*s*, 1H), 7.46 (*d*, *J* = 1.4 Hz, 1H), 7.37–7.28 (*m*, 5H), 7.27 (*d*, *J* = 7.2 Hz, 1H), 7.25–7.19 (*m*, 3H), 7.19–7.13 (*m*, 6H), 7.05 (*d*, *J* = 7.5 Hz, 1H), 5.72 (*s*, 1H), 2.78–2.56 (*m*, 3H), 2.25–2.15 (*m*, 1H), 1.54 (*s*, 3H); ^13^C NMR (125 MHz, DMSO-*d*_6_) *δ* 168.8, 142.9, 141.9, 138.4, 135.7, 135.1, 132.9, 130.5, 129.0, 128.7, 128.6, 128.2, 128.1, 127.1, 126.6, 126.4, 126.0, 123.2, 123.1, 121.5, 113.4, 112.7, 111.4, 47.9, 29.5, 27.6, 22.3; HRMS (ESI) *m*/*z*: [M + H]^+^ calculated for C_33_H_28_ON_2_Br, 547.1380; found, 547.1381.
*N-(2-(2-benzhydryl-6-chloro-1H-indol-3-yl)-3,4-dihydronaphthalen-1-yl)acetamide* (**3ag**). The product **3ag** was obtained by column chromatography on silica gel (eluent: petroleum ether/ethyl acetate = 4:1) as a yellow solid (75.4 mg, yield: 75%); mp 316–317 °C; ^1^H NMR (500 MHz, DMSO-*d*_6_) *δ* 11.01 (*s*, 1H), 8.96 (*s*, 1H), 7.36–7.29 (*m*, 6H), 7.26 (*t*, *J* = 7.3 Hz, 1H), 7.24–7.20 (*m*, 3H), 7.19–7.13 (*m*, 5H), 7.03 (*dd*, *J* = 7.2, 1.5 Hz, 1H), 6.95 (*dd*, *J* = 8.4, 2.0 Hz, 1H), 5.71 (*s*, 1H), 2.76–2.62 (*m*, 3H), 2.21–2.12 (*m*, 1H), 1.49 (*s*, 3H); ^13^C NMR (125 MHz, DMSO-*d*_6_) *δ* 168.8, 143.1, 141.8, 137.9, 136.9, 135.7, 132.9, 130.3, 129.0, 128.7, 128.6, 128.2, 127.3, 127.1, 126.6, 126.4, 126.1, 125.5, 125.1, 123.1, 120.8, 118.9, 113.1, 110.9, 47.8, 29.5, 27.7, 22.2; HRMS (ESI) *m*/*z*: [M + H]^+^ calculated for C_33_H_28_ON_2_Cl, 503.1885; found, 503.1886.
*N-(2-(2-(di-p-tolylmethyl)-1H-indol-3-yl)-3,4-dihydronaphthalen-1-yl)acetamide* (**3ah**). The product **3ah** was obtained by column chromatography on silica gel (eluent: petroleum ether/ethyl acetate = 5:1) as a yellow solid (64.5 mg, yield: 65%); mp 327–328 °C; ^1^H NMR (500 MHz, DMSO-*d*_6_) *δ* 10.87 (*s*, 1H), 8.91 (*s*, 1H), 7.34 (*dd*, *J* = 8.0, 3.6 Hz, 2H), 7.24–7.15 (*m*, 3H), 7.16–7.12 (*m*, 2H), 7.09–6.98 (*m*, 7H), 6.98–6.90 (*m*, 2H), 5.62 (*s*, 1H), 2.79–2.72 (*m*, 3H), 2.26 (*s*, 3H), 2.23 (*s*, 3H), 2.18–2.13 (*m*, 1H), 1.44 (*s*, 3H); ^13^C NMR (125 MHz, DMSO-*d*_6_) *δ* 168.8, 140.6, 139.3, 137.3, 136.5, 135.6, 135.3, 135.2, 133.0, 129.9, 129.0, 128.6, 128.6, 128.0, 127.0, 126.3, 126.3, 126.0, 123.1, 120.7, 119.4, 118.6, 112.5, 111.3, 47.1, 29.7, 27.7, 22.2, 20.6; HRMS (ESI) *m*/*z*: [M + H]^+^ calculated for C_35_H_33_ON_2_, 497.2587; found, 497.2586.
*N-(2-(2-(bis(4-methoxyphenyl)methyl)-1H-indol-3-yl)-3,4-dihydronaphthalen-1-yl)acetamide* (**3ai**). The product **3ai** was obtained by column chromatography on silica gel (eluent: petroleum ether/ethyl acetate = 5:1) as a yellow solid (63.2 mg, yield: 60%); mp 311–312 °C;^1^H NMR (500 MHz, DMSO-*d*_6_) *δ* 10.81 (*s*, 1H), 8.91 (*s*, 1H), 7.34 (*dd*, *J* = 7.9, 3.7 Hz, 2H), 7.21–7.12 (*m*, 5H), 7.09 (*d*, *J* = 8.6 Hz, 2H), 7.06–7.01 (*m*, 2H), 6.93 (*t*, *J* = 7.5 Hz, 1H), 6.88 (*dd*, *J* = 17.5, 8.7 Hz, 4H), 5.60 (*s*, 1H), 3.74 (*s*, 3H), 3.70 (*s*, 3H), 2.76–2.66 (*m*, 3H), 2.25–2.16 (*m*, 1H), 1.51 (*s*, 3H);^13^C NMR (125 MHz, DMSO-*d*_6_) *δ* 168.8, 157.8, 137.7, 136.5, 135.8, 135.6, 134.4, 133.1, 130.1, 129.8, 129.7, 128.0, 127.0, 126.4, 126.3, 126.0, 123.1, 120.7, 119.4, 118.6, 113.8, 113.5, 112.3, 111.3, 55.1, 55.0, 46.2, 29.7, 27.8, 22.3; HRMS (ESI) *m*/*z*: [M + H]^+^ calculated for C_35_H_33_O_3_N_2_, 529.2486; found, 529.2483.
*N-(2-(2-(bis(4-fluorophenyl)methyl)-1H-indol-3-yl)-3,4-dihydronaphthalen-1-yl)acetamide* (**3aj**). The product **3aj** was obtained by column chromatography on silica gel (eluent: petroleum ether/ethyl acetate = 7:1) as a yellow solid (91.2 mg, yield: 91%); mp 333–334 °C; ^1^H NMR (500 MHz, DMSO-*d*_6_) δ 11.04 (*s*, 1H), 9.04 (*s*, 1H), 7.38 (q, *J* = 7.0 Hz, 4H), 7.28–6.84 (*m*, 12H), 5.77 (*s*, 1H), 2.76 (*s*, 3H), 2.24 (*s*, 1H), 1.49 (*s*, 3H); ^13^C NMR (125 MHz, DMSO-*d*_6_) δ 168.9, 163.2 (*d*, *J* = 6.8 Hz), 161.3 (*d*, *J* = 5.8 Hz), 145.6 (*d*, *J* = 6.8 Hz), 144.4 (*d*, *J* = 6.9 Hz), 136.6, 135.7, 135.3, 132.9, 130.6 (*d*, *J* = 8.3 Hz), 130.2, 130.1, 130.1, 127.6, 127.1, 126.5, 126.1 (*d*, *J* = 13.7 Hz), 125.2 (*d*, *J* = 2.6 Hz), 125.0 (*d*, *J* = 2.4 Hz), 123.1, 121.2, 119.7, 118.9, 115.9 (*d*, *J* = 22.1 Hz), 115.3 (*d*, *J* = 21.6 Hz), 113.7 (*d*, *J* = 20.8 Hz), 113.4, 113.3, 111.4, 47.2, 29.7, 27.8, 22.1; ^19^F NMR (471 MHz, DMSO-*d*_6_) *δ* −112.64–−112.70 (*m*, 1F), −113.50–−113.56 (*m*, 1F); HRMS (ESI) *m*/*z*: [M + H]^+^ calculated for C_33_H_27_ON_2_F_2_, 505.2086; found, 505.2084.
*N-(2-(2-(bis(4-chlorophenyl)methyl)-1H-indol-3-yl)-3,4-dihydronaphthalen-1-yl)acetamide* (**3ak**). The product **3ak** was obtained by column chromatography on silica gel (eluent: petroleum ether/ethyl acetate = 7:1) as a yellow solid (79.5 mg, yield: 74%); mp 315–316 °C; ^1^H NMR (600 MHz, DMSO-*d*_6_) *δ* 10.91 (*s*, 1H), 8.99 (*s*, 1H), 7.41 (*dd*, *J* = 16.9, 8.5 Hz, 4H), 7.38–7.32 (*m*, 2H), 7.24 (*d*, *J* = 8.5 Hz, 2H), 7.21–7.13 (*m*, 5H), 7.08–7.01 (*m*, 2H), 6.96 (*t*, *J* = 7.4 Hz, 1H), 5.74 (*s*, 1H), 2.77–2.66 (*m*, 3H), 2.24–2.16 (*m*, 1H), 1.53 (*s*, 3H); ^13^C NMR (150 MHz, DMSO-*d*_6_) *δ* 168.9, 141.9, 140.7, 136.6, 135.7, 135.6, 133.0, 131.3, 131.3, 130.9, 130.5, 130.1, 128.6, 128.2, 127.8, 127.1, 126.5, 126.3, 126.0, 123.0, 121.1, 119.6, 118.8, 113.2, 111.4, 46.6, 29.6, 27.7, 22.2; HRMS (ESI) *m*/*z*: [M + H]^+^ calculated for C_33_H_27_ON_2_Cl_2_, 537.1495; found, 537.1496.
*N-(2-(2-(bis(4-(trifluoromethyl)phenyl)methyl)-1H-indol-3-yl)-3,4-dihydronaphthalen-1-yl)acetamide* (**3al**). The product **3al** was obtained by column chromatography on silica gel (eluent: petroleum ether/ethyl acetate = 5:1) as a yellow solid (77.3 mg, yield: 64%); mp 313–314 °C; ^1^H NMR (500 MHz, DMSO-*d*_6_) *δ* 11.05 (*s*, 1H), 9.04 (*s*, 1H), 7.74 (*t*, *J* = 8.4 Hz, 4H), 7.47 (*d*, *J* = 8.2 Hz, 2H), 7.43–7.28 (*m*, 4H), 7.22–7.12 (*m*, 3H), 7.11–7.05 (*m*, 1H), 7.04–7.01 (*m*, 1H), 6.99–6.94 (*m*, 1H), 5.93 (*s*, 1H), 2.79–2.70 (*m*, 3H), 2.31–2.13 (*m*, 1H), 1.46 (*s*, 3H); ^13^C NMR (125 MHz, DMSO-*d*_6_) *δ* 169.0, 147.3, 146.1, 136.7, 135.7, 134.8, 132.9, 130.2, 129.9, 129.6, 127.8, 127.6, 127.5, 127.4, 127.3, 127.1, 126.6, 126.2, 126.1, 125.7 (q, *J* = 3.7 Hz), 125.5, 125.4, 125.2 (q, *J* = 3.8 Hz), 123.3, 123.2, 123.1, 121.3, 119.7, 119.0, 113.8, 111.5, 47.5, 29.6, 27.7, 22.1; ^19^F NMR (471 MHz, DMSO-*d*_6_) *δ* −60.82 (*s*, 3F), −60.86 (*s*, 3F); HRMS (ESI) *m*/*z*: [M + H]^+^ calculated for C_35_H_27_ON_2_F_6_, 605.2022; found, 605.2015.
*N-(2-(2-(bis(3-fluorophenyl)methyl)-1H-indol-3-yl)-3,4-dihydronaphthalen-1-yl)acetamide* (**3am**). The product **3am** was obtained by column chromatography on silica gel (eluent: petroleum ether/ethyl acetate = 7:1) as a yellow solid (78.6 mg, yield: 78%); mp 325–326 °C; ^1^H NMR (500 MHz, DMSO-*d*_6_) *δ* 11.03 (*s*, 1H), 9.03 (*s*, 1H), 7.43–7.34 (*m*, 4H), 7.21–7.18 (*m*, 1H), 7.17–7.14 (*m*, 2H), 7.12 (*s*, 1H), 7.11–7.02 (*m*, 5H), 7.01–6.94 (*m*, 3H), 5.76 (*s*, 1H), 2.81–2.65 (*m*, 3H), 2.34–2.12 (*m*, 1H), 1.48 (*s*, 3H);^13^C NMR (125 MHz, DMSO-*d*_6_) *δ* 168.9, 163.2 (*d*, *J* = 6.7 Hz), 161.3 (*d*, *J* = 5.8 Hz), 145.6 (*d*, *J* = 6.8 Hz), 144.4 (*d*, *J* = 7.0 Hz), 136.6, 135.7, 135.3, 132.9, 130.7 (*d*, *J* = 8.3 Hz), 130.2, 130.1, 130.1, 127.7, 127.1, 126.5, 126.1 (*d*, *J* = 12.1 Hz), 125.2 (*d*, *J* = 2.2 Hz), 125.0 (*d*, *J* = 2.2 Hz), 123.1, 121.2, 119.7, 118.9, 115.9 (*d*, *J* = 22.1 Hz), 115.3 (*d*, *J* = 21.6 Hz), 113.7 (*d*, *J* = 20.9 Hz), 113.5, 113.3, 111.4, 47.2, 29.7, 27.8, 22.1; ^19^F NMR (471 MHz, DMSO-*d*_6_) *δ* −112.65–−112.71 (*m*, 1F), −113.51–−113.56 (*m*, 1F); HRMS (ESI) *m*/*z*: [M + H]^+^ calculated for C_33_H_27_ON_2_F_2_, 505.2086; found, 505.2084.
*N-(2-(2-(bis(3-chlorophenyl)methyl)-1H-indol-3-yl)-3,4-dihydronaphthalen-1-yl)acetamide* (**3an**). The product **3an** was obtained by column chromatography on silica gel (eluent: petroleum ether/ethyl acetate = 6:1) as a yellow solid (104.2 mg, yield: 97%); mp 313–314 °C; ^1^H NMR (500 MHz, DMSO-*d*_6_) *δ* 11.09 (*s*, 1H), 9.10 (*s*, 1H), 7.41–7.34 (*m*, 5H), 7.34–7.28 (*m*, 2H), 7.27–7.21 (*m*, 1H), 7.22–7.19 (*m*, 2H), 7.19–7.13 (*m*, 2H), 7.12–7.04 (*m*, 2H), 7.06–7.00 (*m*, 1H), 7.01–6.94 (*m*, 1H), 5.76 (*s*, 1H), 2.82–2.73 (*m*, 3H), 2.24–2.14 (*m*, 1H), 1.49 (*s*, 3H); ^13^C NMR (125 MHz, DMSO-*d*_6_) *δ* 168.9, 145.1, 143.9, 136.6, 135.6, 135.1, 133.2, 133.1, 132.9, 130.6, 130.1, 128.7, 128.3, 127.8, 127.7, 127.5, 127.1, 126.8, 126.6, 126.5, 126.2, 126.1, 123.1, 121.3, 119.7, 118.9, 113.5, 111.5, 47.1, 29.6, 27.7, 22.1; HRMS (ESI) *m*/*z*: [M + H]^+^ calculated for C_33_H_27_ON_2_Cl_2_, 537.1495; found, 537.1494.
*N-(2-(2-(bis(3-methoxyphenyl)methyl)-1H-indol-3-yl)-3,4-dihydronaphthalen-1-yl)acetamide* (**3ao**). The product **3ao** was obtained by column chromatography on silica gel (eluent: petroleum ether/ethyl acetate = 5:1) as a yellow solid (64.4 mg, yield: 61%); mp 313–314 °C; ^1^H NMR (500 MHz, DMSO-*d*_6_) *δ* 10.91 (*s*, 1H), 8.94 (*s*, 1H), 7.37–7.32 (*m*, 2H), 7.28–7.22 (*m*, 1H), 7.24–7.17 (*m*, 2H), 7.18–7.11 (*m*, 2H), 7.08–7.01 (*m*, 1H), 7.04–6.98 (*m*, 1H), 6.97–6.91 (*m*, 1H), 6.87–6.79 (*m*, 2H), 6.81–6.73 (*m*, 3H), 6.75 (*d*, *J* = 8.2 Hz, 1H), 5.63 (*s*, 1H), 3.70 (*s*, 3H), 3.67 (*s*, 3H), 2.80–2.67 (*m*, 3H), 2.26–2.15 (*m*, 1H), 1.44 (*s*, 3H); ^13^C NMR (125 MHz, DMSO-*d*_6_) *δ* 168.8, 159.3, 159.1, 145.0, 143.4, 136.6, 136.5, 135.6, 133.0, 130.0, 129.5, 129.1, 127.9, 127.1, 126.4, 126.3, 126.0, 123.1, 121.6, 121.1, 120.9, 119.5, 118.7, 115.7, 114.8, 112.8, 111.4, 111.0, 55.0, 47.8, 29.7, 27.8, 22.1; HRMS (ESI) *m*/*z*: [M + H]^+^ calculated for C_35_H_33_O_3_N_2_, 529.2486; found, 529.2481.
*N-(2-(2-(di-m-tolylmethyl)-1H-indol-3-yl)-3,4-dihydronaphthalen-1-yl)acetamide* (**3ap**). The product **3ap** was obtained by column chromatography on silica gel (eluent: petroleum ether/ethyl acetate = 5:1) as a yellow solid (70.5 mg, yield: 71%); mp 321–322 °C; ^1^H NMR (500 MHz, DMSO-*d*_6_) *δ* 10.81 (*s*, 1H), 8.90 (*s*, 1H), 7.33 (*dd*, *J* = 8.0, 3.9 Hz, 2H), 7.20–7.16 (*m*, 1H), 7.17–7.08 (*m*, 8H), 7.06 (*d*, *J* = 8.5 Hz, 2H), 7.04–7.00 (*m*, 2H), 6.95–6.90 (*m*, 1H), 5.62 (*s*, 1H), 2.76–2.66 (*m*, 3H), 2.29 (*s*, 3H), 2.25 (*s*, 3H), 2.23–2.16 (*m*, 1H), 1.48 (*s*, 3H); ^13^C NMR (125 MHz, DMSO-*d*_6_) *δ* 168.8, 140.6, 139.3, 137.3, 136.5, 135.6, 135.3, 135.2, 133.0, 129.9, 129.0, 128.6, 128.0, 127.0, 126.3, 126.3, 126.0, 123.1, 120.7, 119.4, 118.6, 112.5, 111.4, 47.1, 29.7, 27.8, 22.2, 20.6; HRMS (ESI) *m*/*z*: [M + H]^+^ calculated for C_35_H_33_ON_2_, 497.2587; found, 497.2587.
*N-(2-(2-(di-o-tolylmethyl)-1H-indol-3-yl)-3,4-dihydronaphthalen-1-yl)acetamide* (**3aq**). The product **3aq** was obtained by column chromatography on silica gel (eluent: petroleum ether/ethyl acetate = 5:1) as a yellow solid (86.3 mg, yield: 87%); mp 321–322 °C; ^1^H NMR (600 MHz, DMSO-*d*_6_) *δ* 10.52 (*s*, 1H), 8.54 (*s*, 1H), 7.39–7.33 (*m*, 2H), 7.22–7.13 (*m*, 5H), 7.12–7.07 (*m*, 5H), 7.04 (*t*, *J* = 7.5 Hz, 1H), 6.93 (*t*, *J* = 7.4 Hz, 1H), 6.91–6.87 (*m*, 1H), 6.86–6.83 (*m*, 1H), 5.67 (*s*, 1H), 2.86–2.77 (*m*, 1H), 2.72–2.65 (*m*, 1H), 2.64–2.55 (*m*, 1H), 2.27–2.23 (*m*, 1H), 2.22 (*s*, 3H), 1.95 (*s*, 3H); ^13^C NMR (150 MHz, DMSO-*d*_6_) *δ* 168.7, 141.9, 140.3, 137.3, 136.5, 135.7, 135.6, 134.5, 132.7, 130.5, 130.4, 130.0, 128.4, 128.0, 127.3, 127.0, 126.6, 126.5, 126.3, 126.2, 126.0, 125.8, 125.7, 123.2, 120.7, 119.7, 118.6, 112.6, 111.4, 42.7, 29.7, 27.6, 21.4, 19.0; HRMS (ESI) *m*/*z*: [M + Na]^+^ calculated for C_35_H_32_N_2_ONa, 519.2407; found, 519.2415.
*N-(2-(2-benzhydryl-1H-indol-3-yl)-1-phenylvinyl)acetamide* (**5a**). The product **5a** was obtained by column chromatography on silica gel (eluent: petroleum ether/ethyl acetate = 5:1) as a white solid (58.6 mg, yield: 66%); mp 321–322 °C; ^1^H NMR (500 MHz, DMSO-*d*_6_) *δ* 11.01 (*s*, 1H), 9.22 (*s*, 1H), 7.37–7.26 (*m*, 10H), 7.25–7.16 (*m*, 7H), 7.06–7.00 (*m*, 1H), 6.98–6.92 (*m*, 1H), 6.55 (*s*, 1H), 5.82 (*s*, 1H), 1.67 (*s*, 3H); ^13^C NMR (125 MHz, DMSO-*d*_6_) *δ* 168.8, 142.1, 139.5, 139.0, 136.4, 133.7, 129.0, 128.4, 128.0, 127.0, 126.5, 125.9, 125.5, 121.0, 120.0, 118.9, 114.2, 111.4, 109.5, 48.0, 22.8; HRMS (ESI) *m*/*z*: [M + Na]+ calculated for C_31_H_26_N_2_ONa, 465.1938; found, 465.1950.
*N-(2-(2-benzhydryl-5-chloro-1H-indol-3-yl)-1-phenylvinyl)acetamide* (**5b**). The product **5b** was obtained by column chromatography on silica gel (eluent: petroleum ether/ethyl acetate = 5:1) as a white solid (49.6 mg, yield: 52%); mp 321–322 °C; ^1^H NMR (500 MHz, DMSO-*d*_6_) *δ* 11.19 (*s*, 1H), 9.30 (*s*, 1H), 7.37–7.28 (*m*, 10H), 7.26–7.21 (*m*, 7H), 7.06 (*dd*, *J* = 8.6, 2.1 Hz, 1H), 6.55 (*s*, 1H), 5.86 (*s*, 1H), 1.75 (*s*, 3H); ^13^C NMR (125 MHz, DMSO-*d*_6_) *δ* 168.8, 141.7, 141.4, 138.8, 134.8, 133.8, 129.0, 128.4, 128.0, 127.1, 126.8, 126.6, 125.5, 123.6, 120.7, 119.5, 113.5, 112.9, 109.4, 47.9, 22.7; HRMS (ESI) *m*/*z*: [M + Na]^+^ calculated for C_31_H_25_N_2_ONaCl, 499.1548; found, 499.1560.
*N-(2-(2-benzhydryl-6-methyl-1H-indol-3-yl)-1-phenylvinyl)acetamide* (**5c**). The product **5c** was obtained by column chromatography on silica gel (eluent: petroleum ether/ethyl acetate = 5:1) as a white solid (79.1 mg, yield: 87%); mp 321–322 °C; ^1^H NMR (500 MHz, DMSO-*d*_6_) *δ* 10.88 (s, 1H), 9.20 (*s*, 1H), 7.37–7.27 (*m*, 8H), 7.25–7.19 (*m*, 8H), 7.13 (*s*, 1H), 6.90–6.86 (*m*, 1H), 6.55 (*s*, 1H), 5.81 (*s*, 1H), 2.31 (*s*, 3H), 1.70 (*s*, 3H); ^13^C NMR (125 MHz, DMSO-*d*_6_) *δ* 168.8, 142.1, 139.6, 139.1, 134.7, 133.3, 129.0, 128.3, 128.0, 127.2, 126.9, 126.5, 126.0, 125.4, 122.3, 120.0, 114.4, 111.1, 109.1, 48.0, 22.8, 21.2; HRMS (ESI) *m*/*z*: [M + Na]^+^ calculated for C_32_H_28_N_2_ONa, 479.2094; found, 479.2101.
*N-(2-(2-(di-p-tolylmethyl)-1H-indol-3-yl)-1-phenylvinyl)acetamide* (**5d**). The product **5d** was obtained by column chromatography on silica gel (eluent: petroleum ether/ethyl acetate = 5:1) as a white solid (63.6 mg, yield: 68%); mp 321–322 °C; ^1^H NMR (500 MHz, DMSO-*d*_6_) *δ* 10.91 (*s*, 1H), 9.20 (*s*, 1H), 7.36–7.26 (*m*, 6H), 7.23 (*t*, *J* = 7.0 Hz, 1H), 7.09 (*s*, 8H), 7.05–7.01 (*m*, 1H), 6.97–6.92 (*m*, 1H), 6.51 (*s*, 1H), 5.72 (*s*, 1H), 2.26 (*s*, 6H), 1.71 (*s*, 3H); ^13^C NMR (125 MHz, DMSO-*d*_6_) *δ* 168.8, 140.0, 139.2, 139.0, 136.3, 135.5, 133.4, 128.9, 128.0, 126.9, 125.9, 125.4, 120.8, 119.9, 118.8, 114.3, 111.4, 109.2, 47.3, 22.9, 20.6; HRMS (ESI) *m*/*z*: [M + Na]^+^ calculated for C_33_H_30_N_2_ONa, 493.2251; found, 493.2257.
*N-(2-(2-(bis(4-fluorophenyl)methyl)-1H-indol-3-yl)-1-phenylvinyl)acetamide* (**5e**). The product **5e** was obtained by column chromatography on silica gel (eluent: petroleum ether/ethyl acetate = 5:1) as a white solid (66.7 mg, yield: 70%); mp 321–322 °C; ^1^H NMR (500 MHz, DMSO-*d*_6_) *δ* 11.08 (*s*, 1H), 9.26 (*s*, 1H), 7.41–7.28 (*m*, 8H), 7.25–7.21 (*m*, 1H), 7.09–7.01 (*m*, 7H), 7.00–6.93 (*m*, 1H), 6.59 (*s*, 1H), 5.91 (*s*, 1H), 1.67 (*s*, 3H); ^13^C NMR (125 MHz, DMSO-*d*_6_) *δ* 168.7, 163.2, 161.3, 144.4 (*d*, *J* = 7.0 Hz), 138.9, 137.9, 136.5, 134.1, 130.4 (*d*, *J* = 8.4 Hz), 128.0, 127.1, 125.8, 125.6, 125.2 (*d*, *J* = 2.3 Hz), 121.3, 120.1, 119.1, 115.7 (*d*, *J* = 21.9 Hz), 113.8, 113.7, 113.5, 111.5, 110.1, 47.2, 22.8; HRMS (ESI) *m*/*z*: [M + Na]^+^ calculated for C_31_H_24_N_2_ONaF_2_, 501.1749; found, 501.1758.
*N-(2-(2-benzhydryl-1H-indol-3-yl)-1-(4-fluorophenyl)vinyl)acetamide* (**5f**). The product **5f** was obtained by column chromatography on silica gel (eluent: petroleum ether/ethyl acetate = 5:1) as a white solid (72.0 mg, yield: 78%); mp 321–322 °C; ^1^H NMR (600 MHz, DMSO-*d*_6_) *δ* 11.02 (*s*, 1H), 9.25 (*s*, 1H), 7.39–7.36 (*m*, 2H), 7.35–7.28 (*m*, 6H), 7.26–7.21 (*m*, 6H), 7.15–7.10 (*m*, 2H), 7.07–7.03 (*m*, 1H), 6.98–6.94 (*m*, 1H), 6.52 (*s*, 1H), 5.83 (*s*, 1H), 1.69 (*s*, 3H);^13^C NMR (150 MHz, DMSO-*d*_6_) *δ* 168.9, 160.6, 142.1, 139.5, 136.4, 135.5, 132.7, 129.0, 128.3, 127.3 (*d*, *J* = 8.0 Hz), 126.5, 125.8, 121.0, 119.9, 118.9, 114.7 (*d*, *J* = 21.4 Hz), 114.0, 111.4, 109.4, 48.0, 22.8; HRMS (ESI) *m*/*z*: [M + Na]^+^ calculated for C_31_H_25_N_2_ONaF, 483.1844; found, 483.1843.
*N-(2-(2-benzhydryl-1H-indol-3-yl)-1-(2-chlorophenyl)vinyl)acetamide* (**5g**). The product **5g** was obtained by column chromatography on silica gel (eluent: petroleum ether/ethyl acetate = 5:1) as a white solid (61.8 mg, yield: 65%); mp 344–345 °C; ^1^H NMR (500 MHz, DMSO-*d*_6_) δ 11.05 (*s*, 1H), 9.30 (*s*, 1H), 7.40–7.27 (*m*, 11H), 7.27–7.20 (*m*, 7H), 7.10–7.03 (*m*, 1H), 7.00–6.96 (*m*, 1H), 6.18 (*s*, 1H), 5.78 (*s*, 1H), 1.53 (*s*, 3H); ^13^C NMR (125 MHz, DMSO-*d*_6_) δ 168.2, 142.1, 138.8, 138.4, 136.4, 132.6, 131.1, 130.9, 129.5, 129.0, 128.4, 126.7, 126.5, 126.3, 121.1, 119.6, 118.9, 116.1, 111.4, 108.6, 48.1, 22.3; HRMS (ESI) *m*/*z*: [M + H]^+^ calculated for C_31_H_26_ClN_2_O, 477.1728; found, 477.1729.
*N-(2-(2-benzhydryl-1H-indol-3-yl)-1-(o-tolyl)vinyl)acetamide* (**5h**). The product **5h** was obtained by column chromatography on silica gel (eluent: petroleum ether/ethyl acetate = 5:1) as a white solid (68.3 mg, yield: 75%); mp 301–302 °C; ^1^H NMR (500 MHz, DMSO-*d*_6_) *δ* 10.97 (*s*, 1H), 9.23 (*s*, 1H), 7.38–7.28 (*m*, 6H), 7.27–7.20 (*m*, 7H), 7.17–7.12 (*m*, 3H), 7.09–7.03 (*m*, 1H), 7.00–6.94 (*m*, 1H), 6.02 (*s*, 1H), 5.78 (*s*, 1H), 2.28 (*s*, 3H), 1.53 (*s*, 3H); ^13^C NMR (125 MHz, DMSO-*d*_6_) *δ* 168.0, 142.2, 139.9, 138.4, 136.4, 135.1, 134.7, 130.0, 129.0, 128.8, 128.3, 126.8, 126.5, 126.4, 125.3, 120.9, 119.6, 118.8, 114.9, 111.4, 108.9, 48.1, 22.4, 20.3; HRMS (ESI) *m*/*z*: [M + H]^+^ calculated for C_32_H_29_N_2_O, 457.2274; found, 457.2271.

## 4. Conclusions

In summary, we have developed the regioselective C3 alkylation of 2-indolylmethanols with cyclic and acyclic enamides under mild reaction conditions. A variety of hybrids of indoles and enamides (40 examples) were obtained in good to excellent yields (up to 98%) with excellent regioselectivities. This protocol represents the first example of C3 alkylation of 2-indolylmethanols with enamides. The potential application of these compounds is under investigation in our laboratory.

## Figures and Tables

**Table 1:**
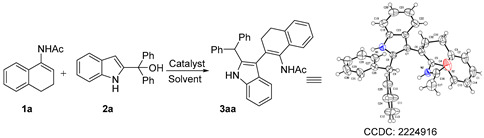
Optimization of the reaction conditions ^a^.

Entry	Catalyst	Solvent	Time	Yield (%) ^b^
1	CPA	Toluene	2	35
2 ^c^	AcOH	Toluene	72	NR
3 ^c^	TFA	Toluene	72	NR
4	TsOH	Toluene	8	38
5	Sc(OTf)_3_	Toluene	3	NR
6	Cu(OTf)_2_	Toluene	3	NR
7	AlCl_3_	Toluene	2	40
8	AlCl_3_	THF	36	NR
9	AlCl_3_	MeCN	3	86
10	AlCl_3_	CHCl_3_	2	80
11	AlCl_3_	DCM	8	70
12	AlCl_3_	DCM	2	88

^a^ Unless otherwise noted, the reactions were performed with **1a** (0.20 mmol), **2a** (0.20 mmol), catalyst (0.04 mmol) and 4Å MS (50 mg) in 2.0 mL solvent. ^b^ Isolated yield of the product. ^c^ The reaction was run at 70 °C.

## Data Availability

The details of the data supporting the report results in this research were included in the paper and Supplementary Materials.

## Supplementary Materials

The following supporting information can be downloaded at https://www.mdpi.com/article/10.3390/molecules28083341/s1.
