# Supplementary material for: Regioselective Reaction of 2-Indolylmethanols with Enamides
Source: Molecules. 2023 Apr 10;28(8):3341. doi: 10.3390/molecules28083341 (PMC10140953; doi:10.3390/molecules28083341)
Supplement: Supplementary file 1 [file molecules-28-03341-s001.zip › molecules-2345454-supplementary.pdf]

Contents

A. General information 2
B. Screening of the CPA 2
C. X-Ray Crystallographic Data of 3aa..3
D. Copies of NMR Spectrum...5
E. Copies of HRMS spectrum..49

A. General information

Unless otherwise specified, all reactions were carried out under a nitrogen atmosphere in anhydrous conditions. All chemicals which are commercially available were used without further purification unless otherwise noted. All the solvents were purified according to the standard procedures. Analytical thin-layer chromatography (TLC) was performed on silica gel plates (GF-254) using UV-light (254 and 365 nm). Flash chromatography was conducted on silica gel (200-300 mesh). NMR spectra were recorded at ambient temperature in $CDCl_3$ and DMSO on a Bruker AMX 500 (^1H NMR at 500 MHz and ^{13}C NMR at 125 MHz) or on a AVANCE III (^1H NMR at 400 MHz and ^{13}C NMR at 100 MHz) spectrometer. Chemical shifts were reported in parts per million (ppm) downfield from an internal standard, tetramethylsilane (0 ppm). High resolution mass spectra were obtained on a Agilent 6200 Q-TOF MS and Waters G2-S QTOF. The substrates were prepared by know methods.[1,2]

B. Screening of the CPA

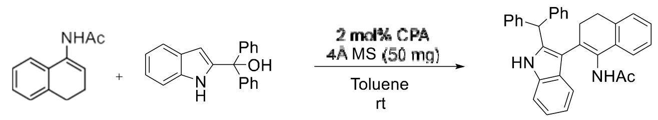

1a **2a** **3aa**

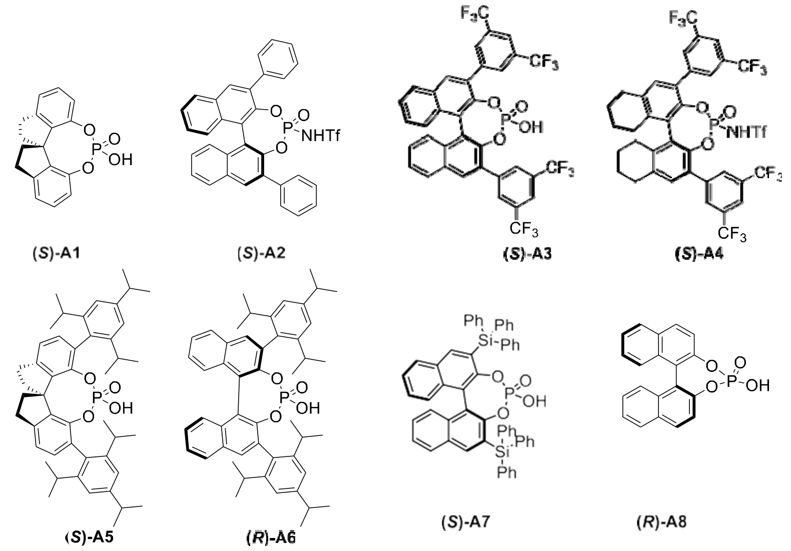

Entry	Acid	Time (h)	Yield (%)[b]	Ee (%)
1	A1	36	15	0
2[c]	A2	72	NR	--
3[c]	A3	72	NR	--
4	A4	36	17	0
5[c]	A5	72	NR	--
6[c]	A6	72	NR	--
7[c]	A7	72	NR	--
8	A8	2	35	0

[a]Unless otherwise noted, the reactions were performed with **1a** (0.20 mmol), **2a** (0.20

mmol) in toluene (2 ml) at room temperature. [b] Isolated yield of the product. [c] The reaction was run at 70 °C.

C. X-Ray Crystallographic Data of 3aa

Crystallization procedure of 3aa: Single crystals of **3aa** were obtained by slow evaporation of a solution containing **3aa** in the mixture of DCM and *n*-Hexane at room temperature. A suitable crystal was selected and the crystal data and structure refinement results for compound **3aa** are listed in the **Table S1**.

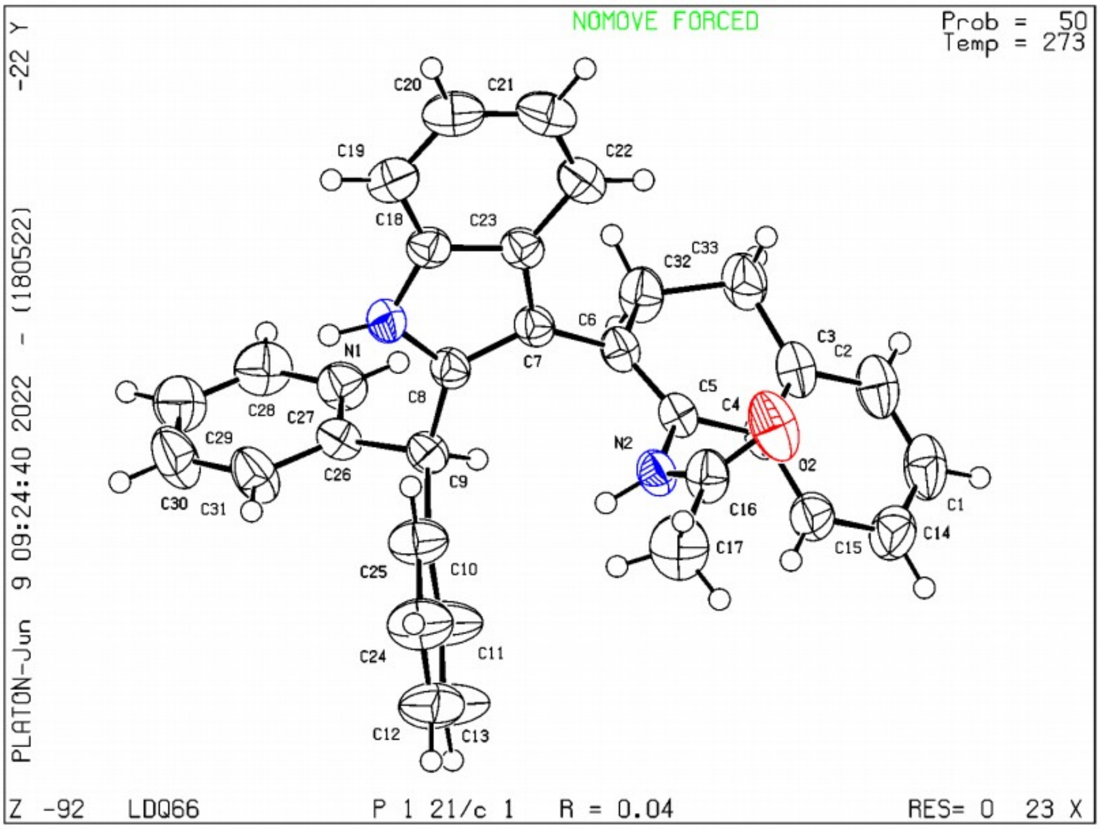

Figure S1. ORTEP view of the compound **3aa** with thermal ellipsoids drawn

Table S1. Crystal data and structure refinement for **3aa.**

Bond precision: C-C = 0.0019 A Wavelength=1.54178

Cell:	a=8.9311 (1)	b=15.3156 (2)	c=19.5342 (3)
	alpha=90	beta=102.695(1)	gamma=90

Temperature: 273 K

	Calculated	Reported
Volume	2606.67(6)	2606.67(6)
Space group	P 21/c	P 1 21/c 1
Hall group	-P 2ybc	-P 2ybc
Moiety formula:	C_{33} H_{28} N_2O	?
Sum formula:	C_{33} H_{28} N_2O	C_{33} H_{28} N_2O
Mr:	468.57	468.57

Dx, g cm^{-3}:	1.194	1.194
Z:	4	4
Mu (mm^{-1}):	0.558	0.558
F000:	992.0	992.0
F000’:	994.68	
h, k, lmax:	11,19,24	11,19,24
Nref:	5662	5651
Tmin, Tmax:	0.921,0.943	0.660,0.940
Tmin’:	0.886	

Correction method = # Reported T Limits: Tmin = 0.660 Tmax = 0.940
AbsCorr = MULTI - SCAN

Data completeness = 0.998 Theta (max) = 79.370

R (reflections) = 0.0446 (5033) wR_2(reflections) = 0.1186 (5651)
S = 1.034 Npar= 330

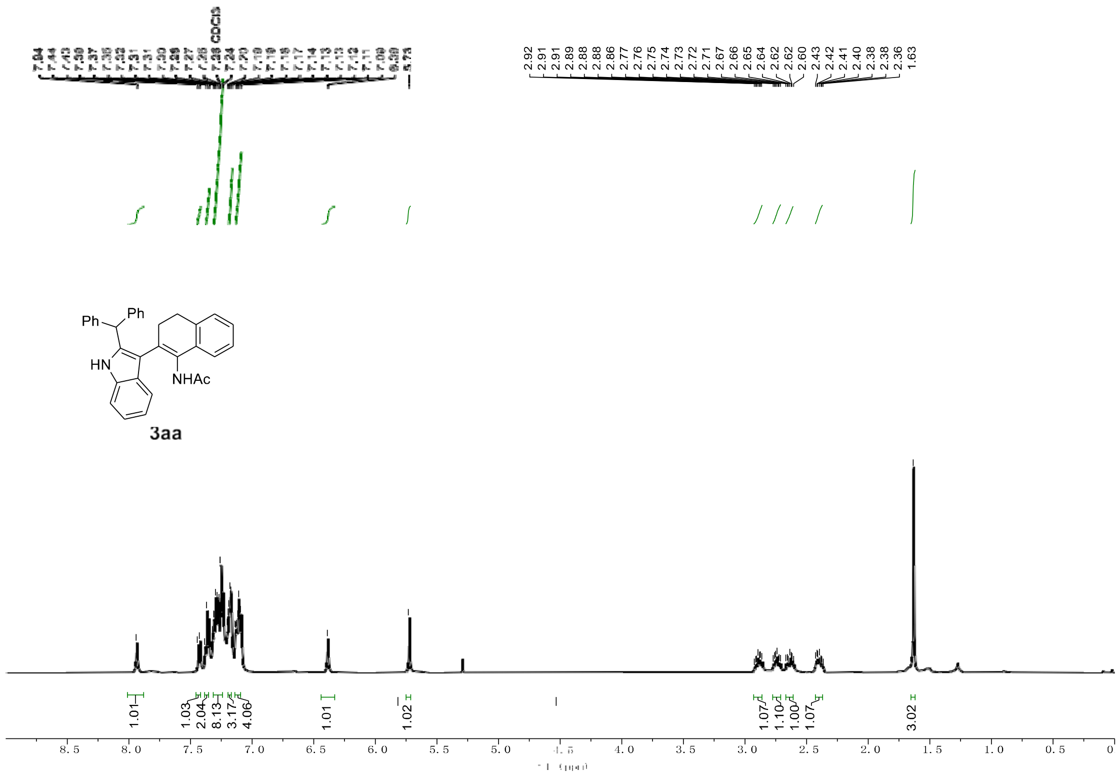
2.92
2.91
2.89
2.88
2.86
2.77
2.76
2.75
2.74
2.73
2.72
2.71
2.67
2.66
2.65
2.64
2.62
2.62
2.60
2.43
2.42
2.41
2.40
2.38
2.38
2.36
1.63
Ph
Ph
HN
NHAc
3aa
1.01
1.03
2.04
8.13
3.17
4.06
1.01
1.02
1.07
1.10
1.00
1.07
3.02
8.5
8.0
7.5
7.0
6.5
6.0
5.5
5.0
4.0
3.5
3.0
2.5
2.0
1.5
1.0
0.5
0

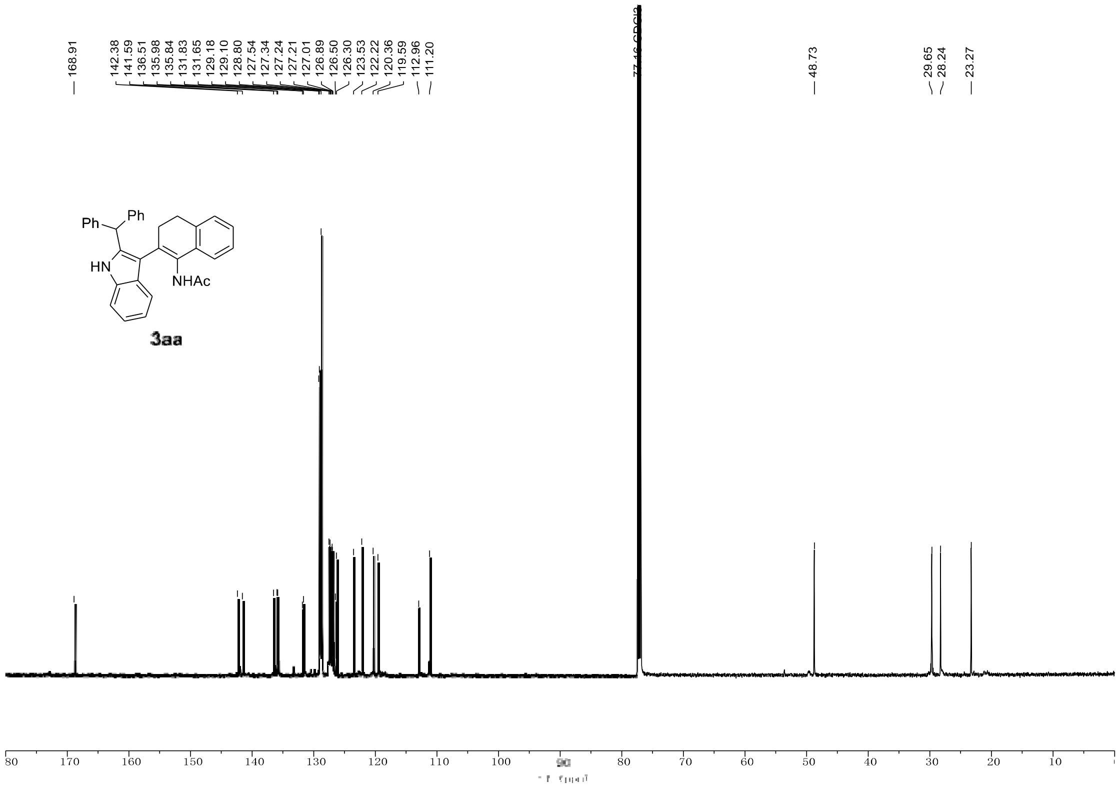
168.91
142.38
141.59
136.51
135.98
135.84
131.83
131.65
129.18
129.10
128.80
127.54
127.34
127.24
127.21
127.01
126.89
126.50
126.30
123.53
122.22
120.36
119.59
112.96
111.20
48.73
29.65
28.24
23.27
Ph
Ph
HN
NHAc
3aa
170
160
150
140
130
120
110
100
80
70
60
50
40
30
20
10

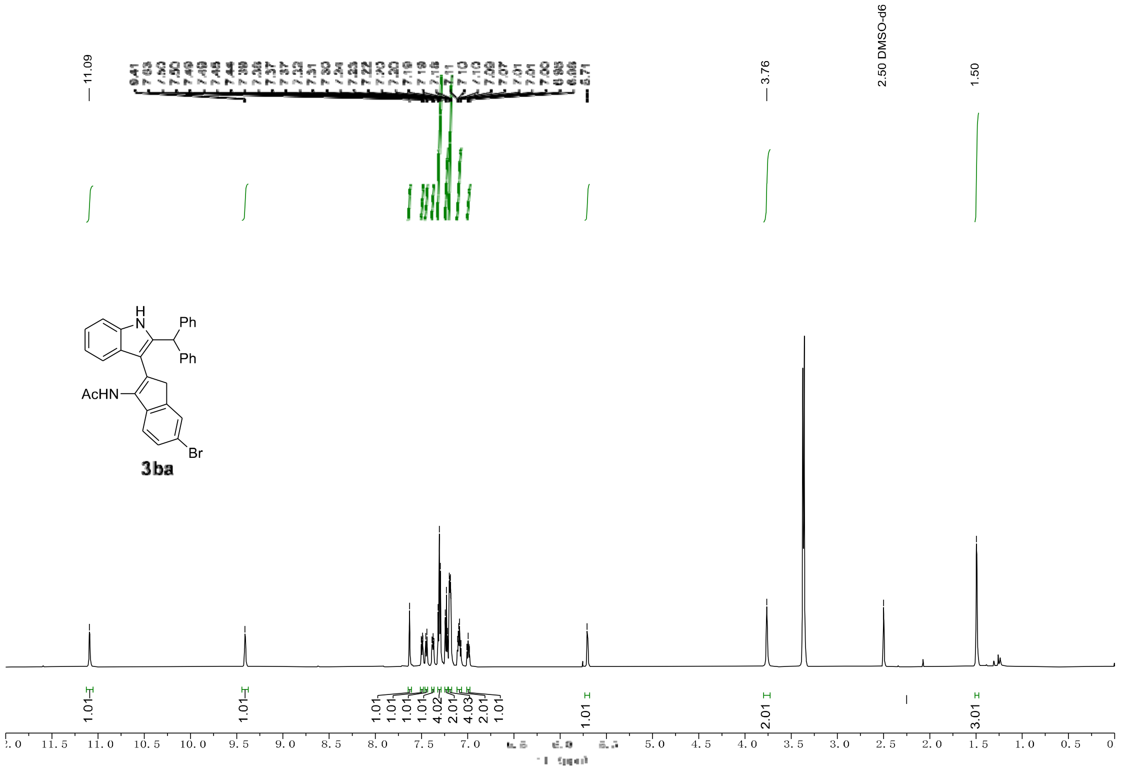
11.09
3.76
2.50 DMSO-d6
1.50
AcHN
Ph
Ph
Br
3ba
1.01
1.01
1.01
1.01
1.01
1.01
4.02
2.01
4.03
2.01
1.01
1.01
2.01
3.01

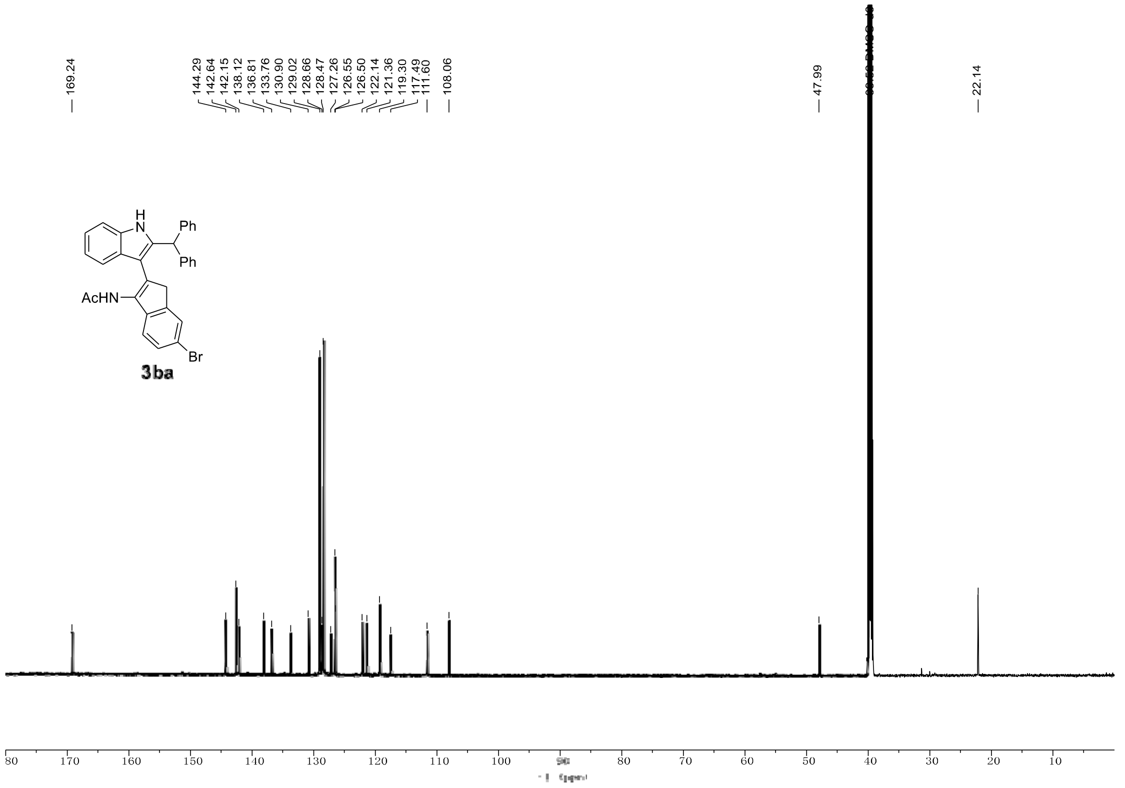
169.24
144.29
142.64
142.15
138.12
136.81
133.76
130.90
129.02
128.66
128.47
127.26
126.55
126.50
122.14
121.36
119.30
117.49
111.60
108.06
47.99
22.14
AcHN
Ph
Ph
Br
3ba

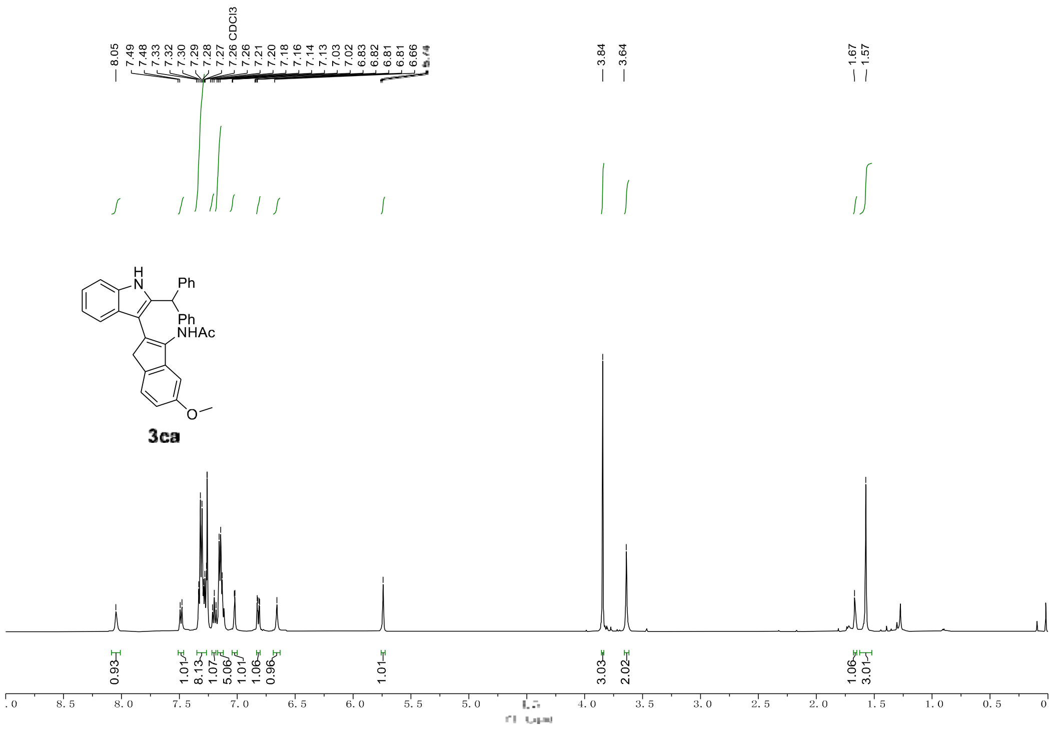
8.05
7.49
7.48
7.33
7.32
7.30
7.29
7.28
7.27
7.26 CDCl3
7.26
7.21
7.20
7.18
7.16
7.14
7.13
7.03
7.02
6.83
6.82
6.81
6.81
6.66
5.74
3.84
3.64
1.67
1.57
Ph
Ph
NHAc
3ca
0.93
1.01
8.13
1.07
5.06
1.01
1.06
0.96
1.01
3.03
2.02
1.06
3.01

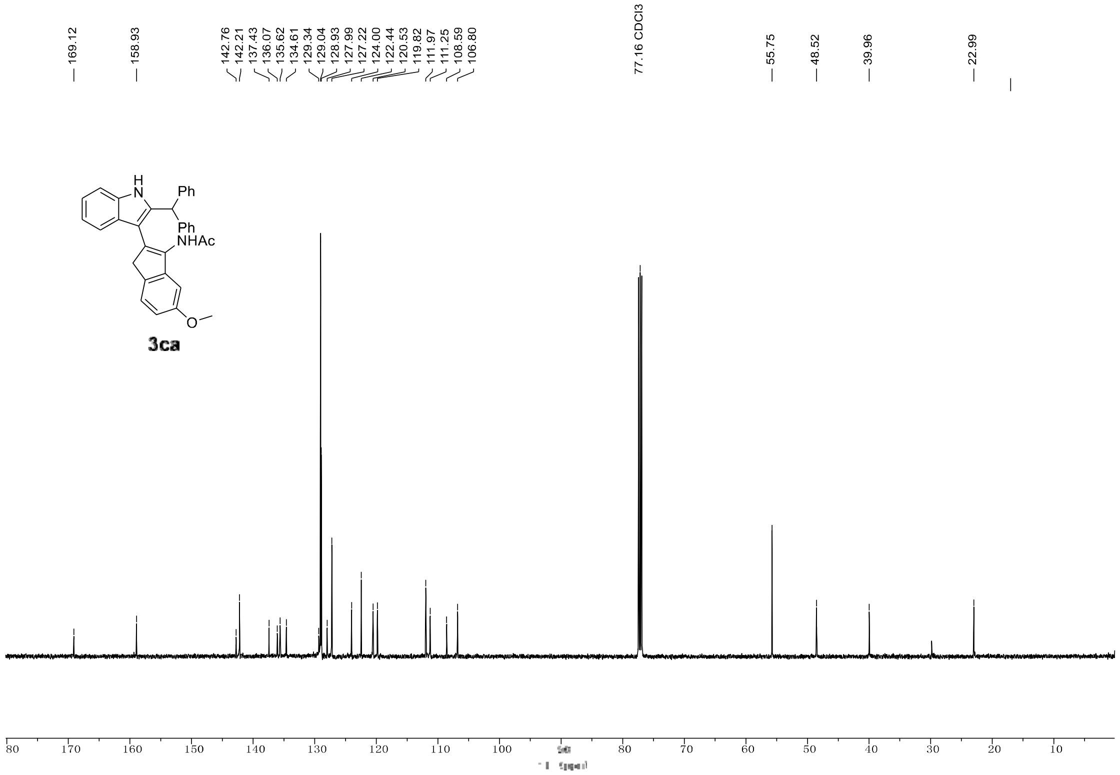
169.12
158.93
142.76
142.21
137.43
136.07
135.62
134.61
129.34
129.04
128.93
127.99
127.22
124.00
122.44
120.53
119.82
111.97
111.25
108.59
106.80
77.16 CDCl3
55.75
48.52
39.96
22.99
Ph
Ph
NHAc
3ca

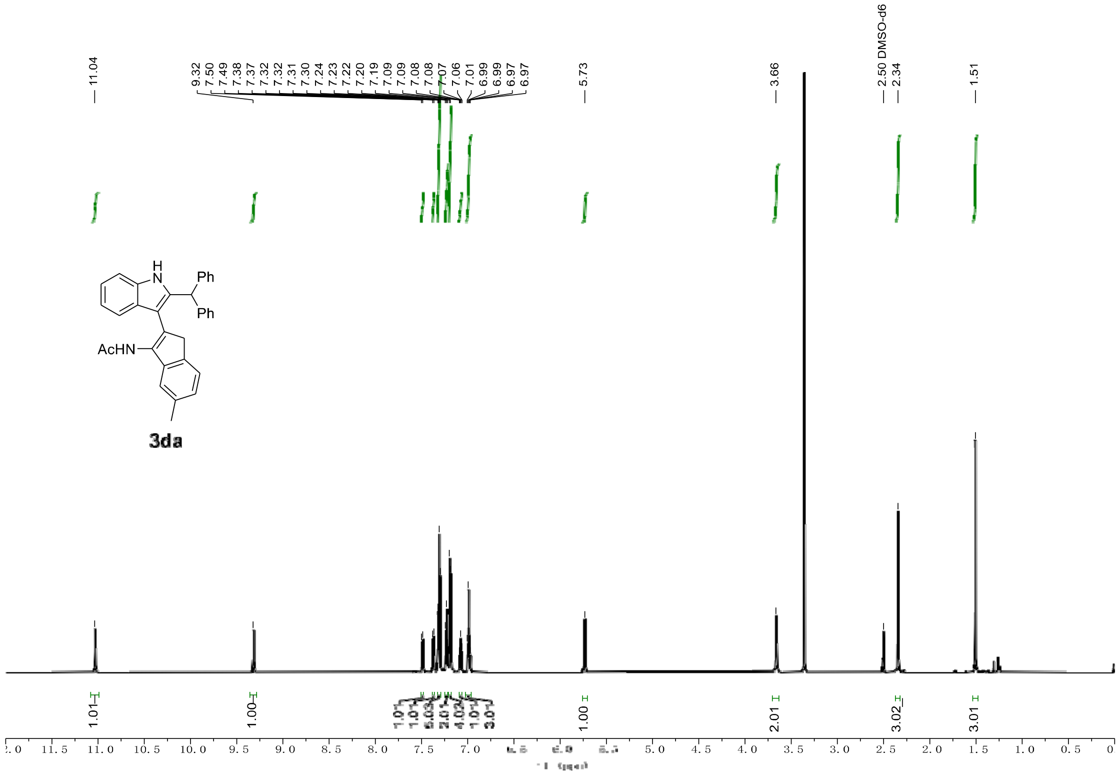
11.04
9.32
7.50
7.49
7.38
7.37
7.32
7.32
7.31
7.30
7.24
7.23
7.22
7.20
7.19
7.09
7.09
7.08
7.08
7.07
7.06
7.01
6.99
6.99
6.97
6.97
5.73
3.66
2.50 DMSO-d6
2.34
1.51
Ph
Ph
AcHN
H
N
3da
1.01
1.00
1.00
2.01
3.02
3.01

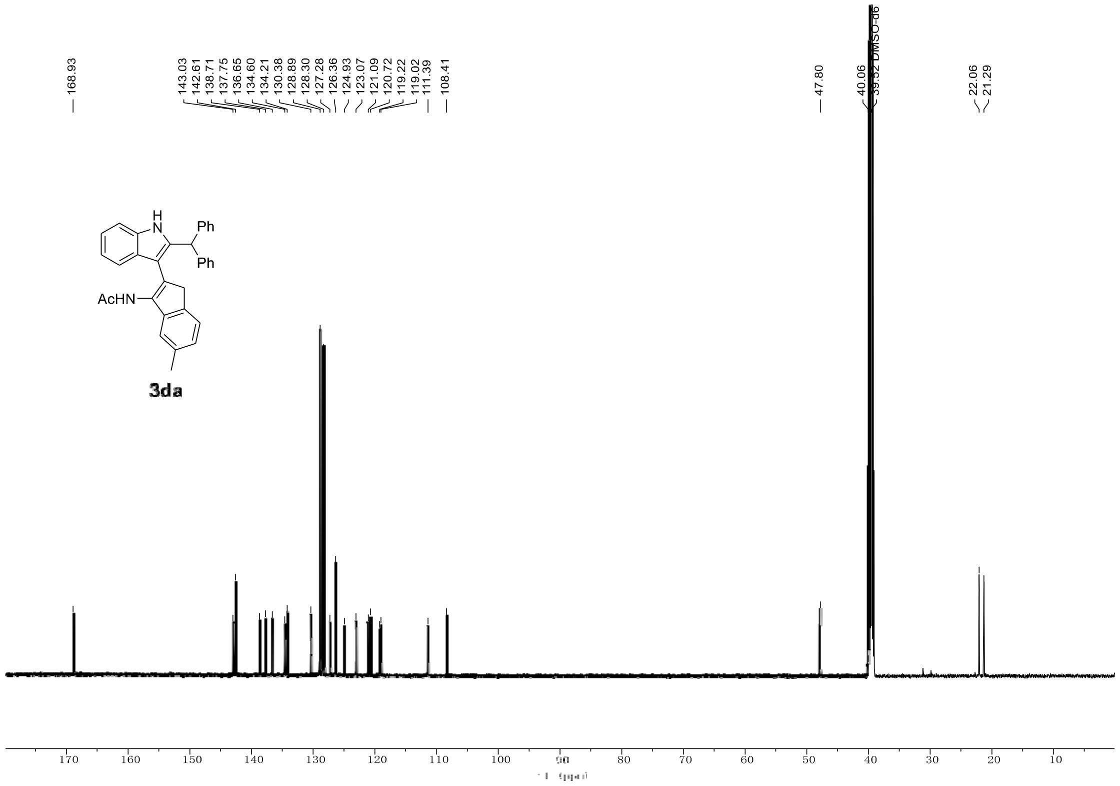
168.93
143.03
142.61
138.71
137.75
136.65
134.60
134.21
130.38
128.89
128.30
127.28
126.36
124.93
123.07
121.09
120.72
119.22
119.02
111.39
108.41
47.80
40.06
39.32 DMSO-d6
22.06
21.29
Ph
Ph
AcHN
H
N
3da

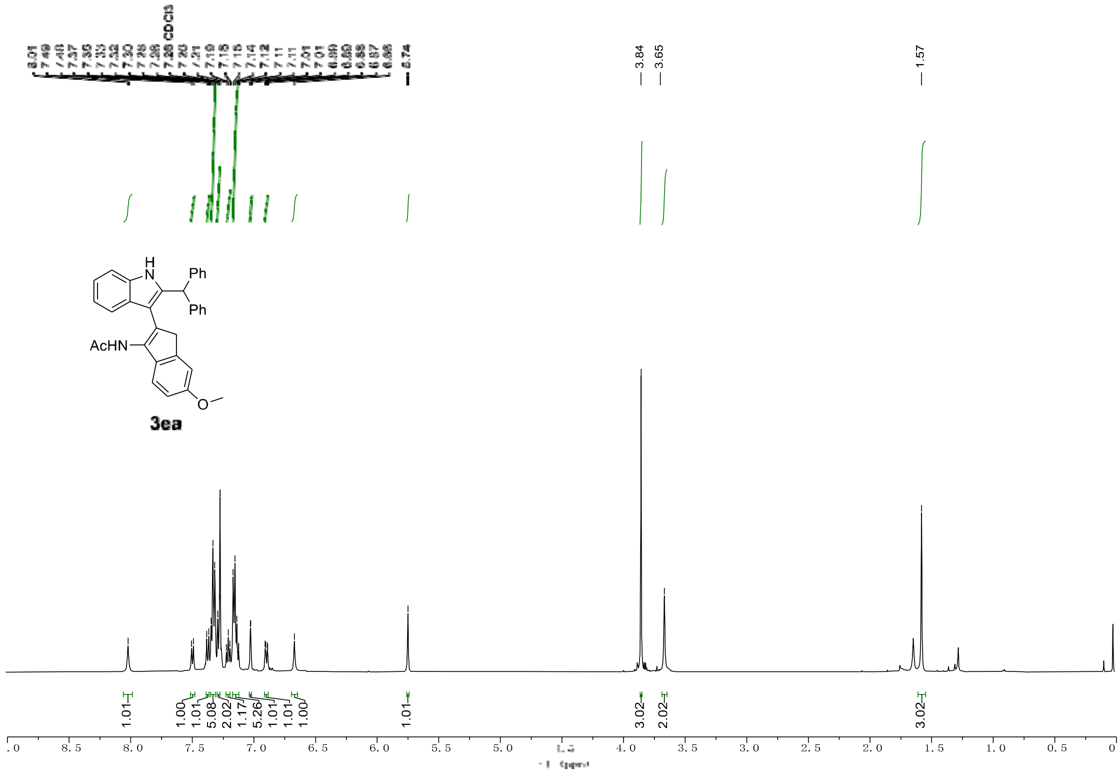

CDCl3
3.84
3.65
1.57
Ph
Ph
AcHN
O
3ea
1.01
1.00
1.01
5.08
2.02
1.17
5.26
1.01
1.01
1.00
1.01
3.02
2.02
3.02

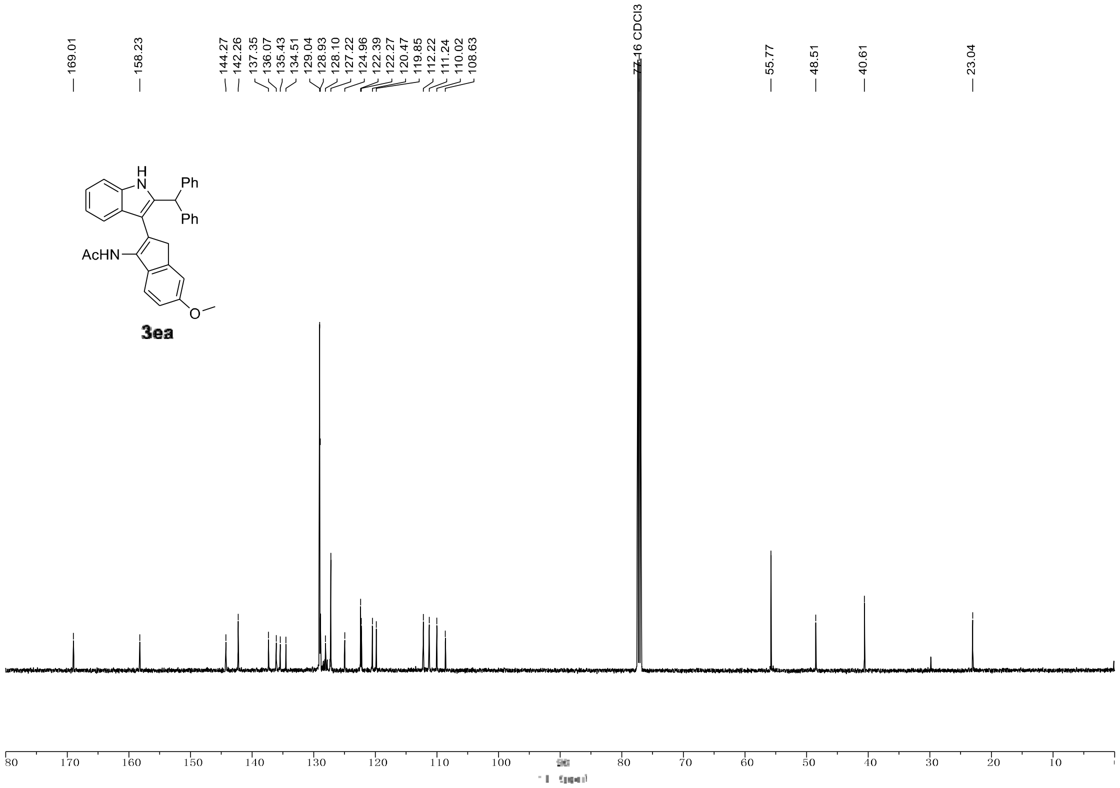

169.01
158.23
144.27
142.26
137.35
136.07
135.43
134.51
129.04
128.93
128.10
127.22
124.96
122.39
122.27
120.47
119.85
112.22
111.24
110.02
108.63
77.16 CDCl3
55.77
48.51
40.61
23.04
Ph
Ph
AcHN
O
3ea
170
160
150
140
130
120
110
100
80
70
60
50
40
30
20
10

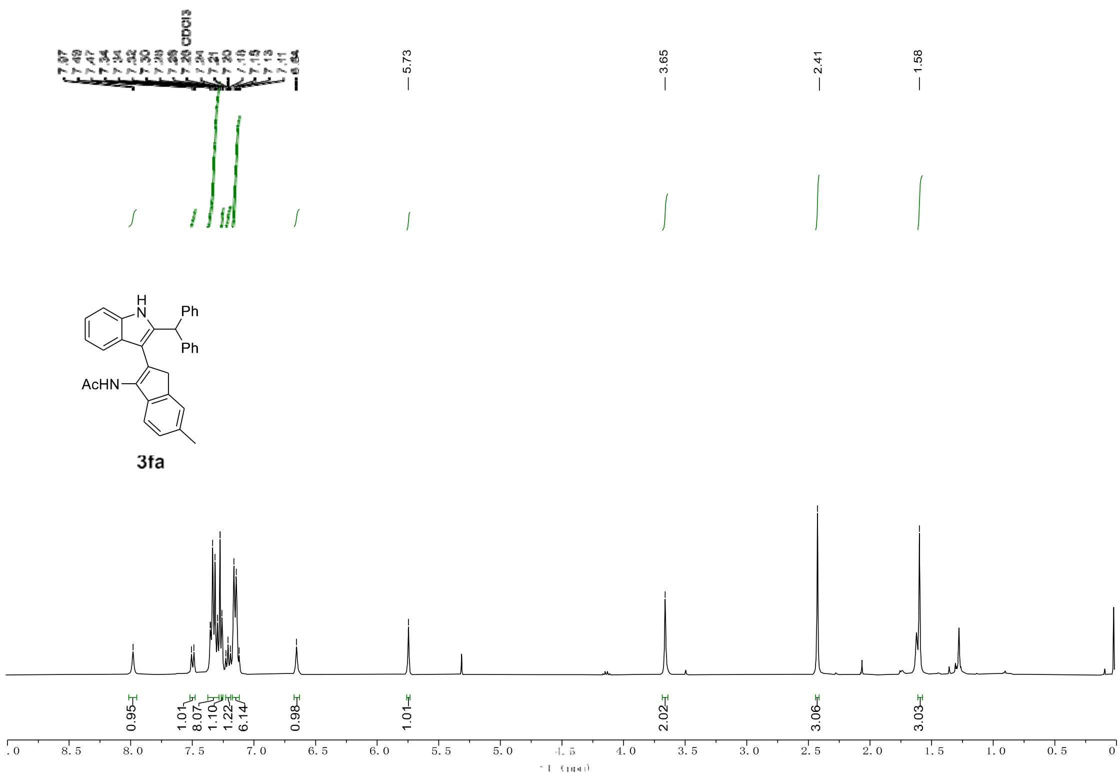

CDCl3
5.73
3.65
2.41
1.58
H
N
Ph
Ph
AcHN
3fa
0.95
1.01
8.07
1.10
1.22
6.14
0.98
1.01
2.02
3.06
3.03
8.5
8.0
7.5
7.0
6.5
6.0
5.5
5.0
4.0
3.5
3.0
2.5
2.0
1.5
1.0
0.5
0

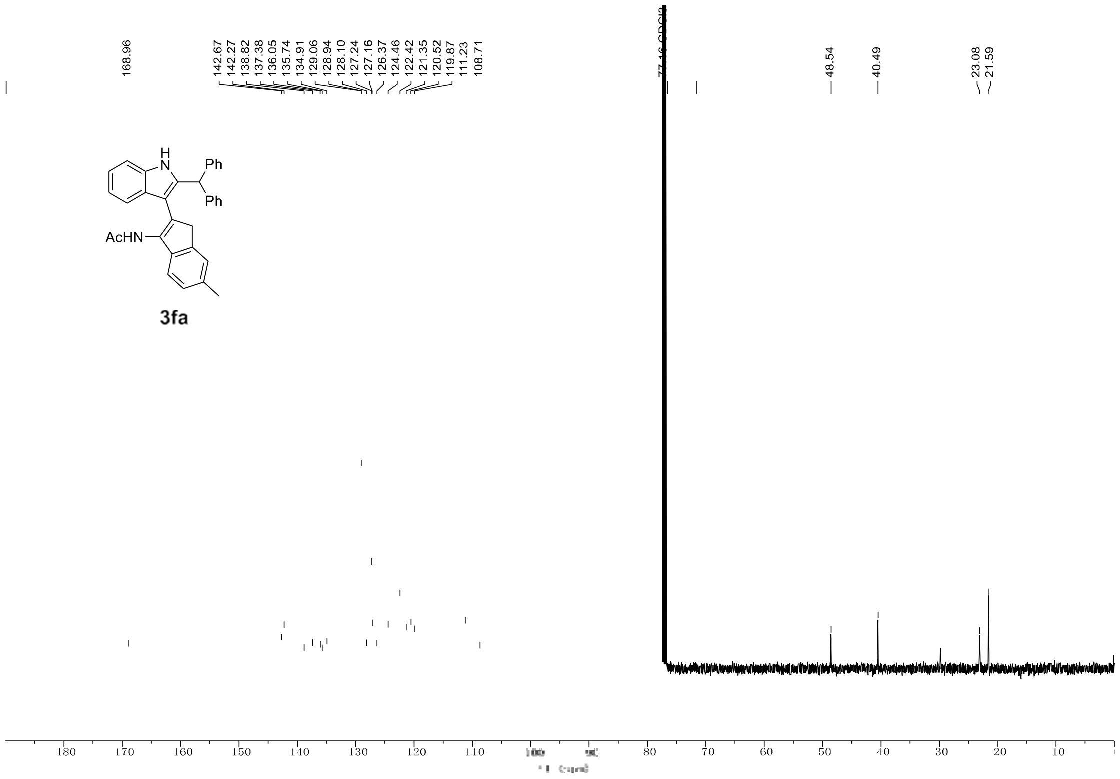

168.96
142.67
142.27
138.82
137.38
136.05
135.74
134.91
129.06
128.94
128.10
127.24
127.16
126.37
124.46
122.42
121.35
120.52
119.87
111.23
108.71
48.54
40.49
23.08
21.59
H
N
Ph
Ph
AcHN
3fa
180
170
160
150
140
130
120
110
80
70
60
50
40
30
20
10

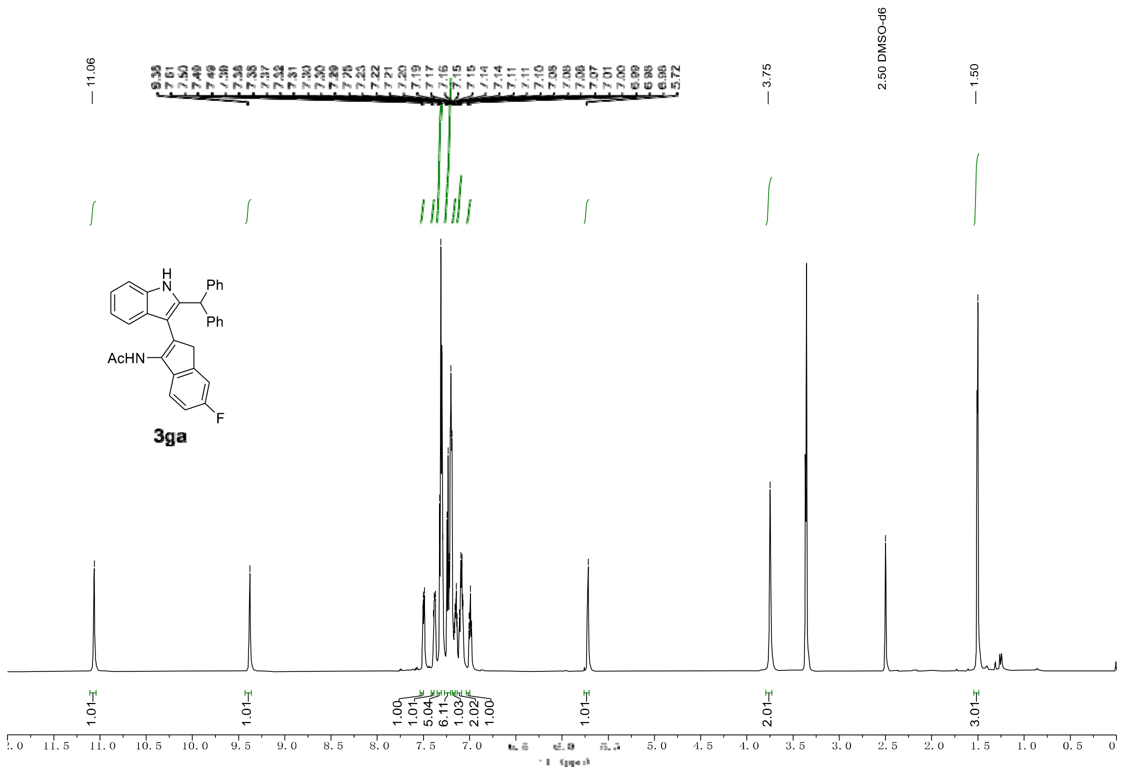

11.06
3.75
2.50 DMSO-d6
1.50
AcHN
Ph
Ph
F
3ga
1.01
1.01
1.00
1.01
5.04
6.11
1.03
2.02
1.00
1.01
2.01
3.01
f1 (ppm)

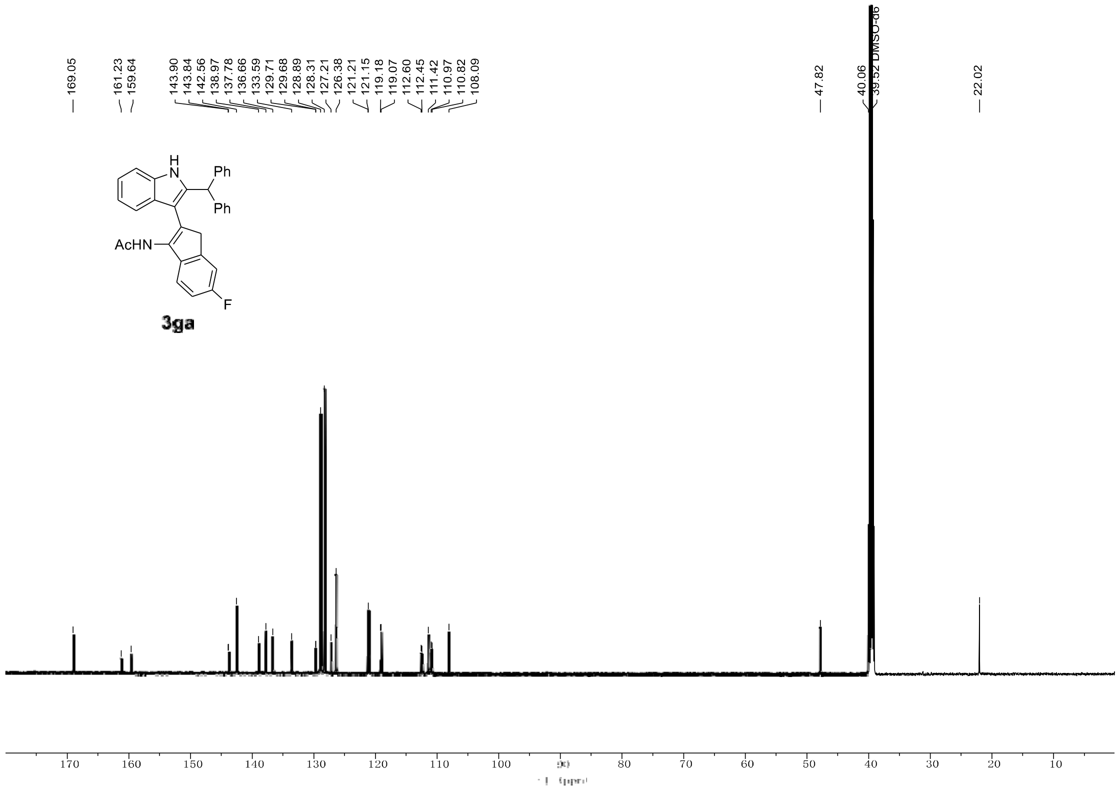

169.05
161.23
159.64
143.90
143.84
142.56
138.97
137.78
136.66
133.59
129.71
129.68
128.89
128.31
127.21
126.38
121.21
121.15
119.18
119.07
112.60
112.45
111.42
110.97
110.82
108.09
47.82
40.06
39.52 DMSO-d6
22.02
AcHN
Ph
Ph
F
3ga
f1 (ppm)

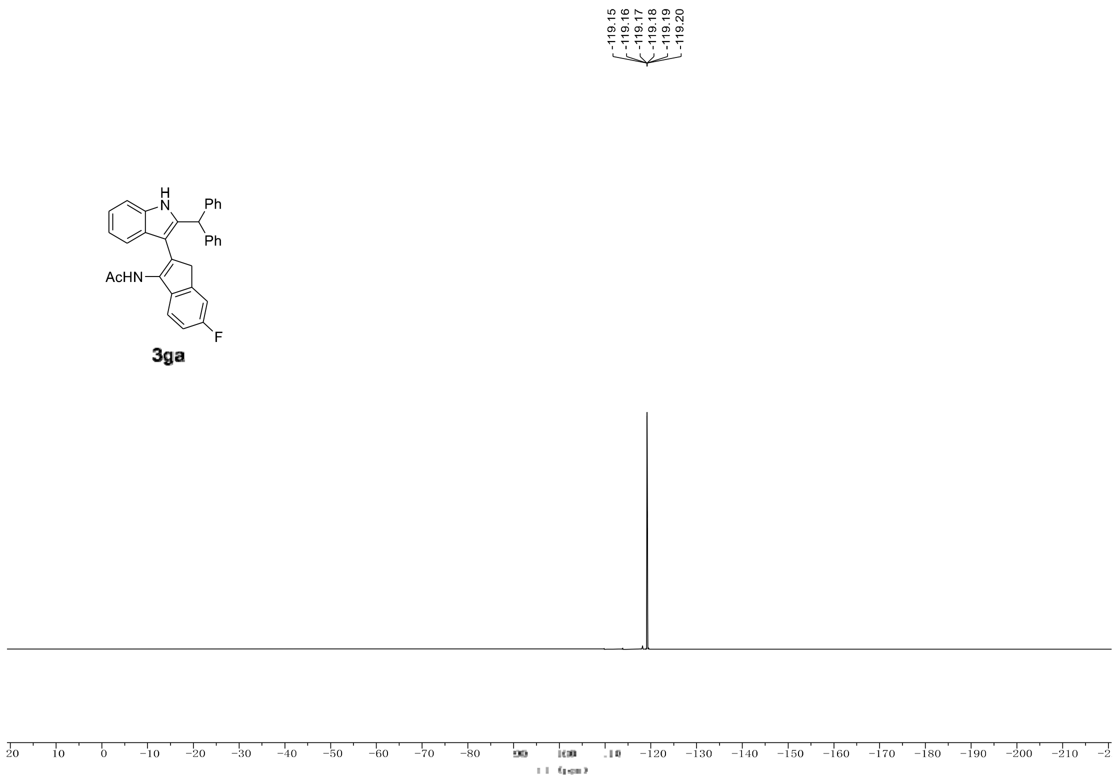

-119.15
-119.16
-119.17
-119.18
-119.19
-119.20
Ph
Ph
AcHN
F
3ga

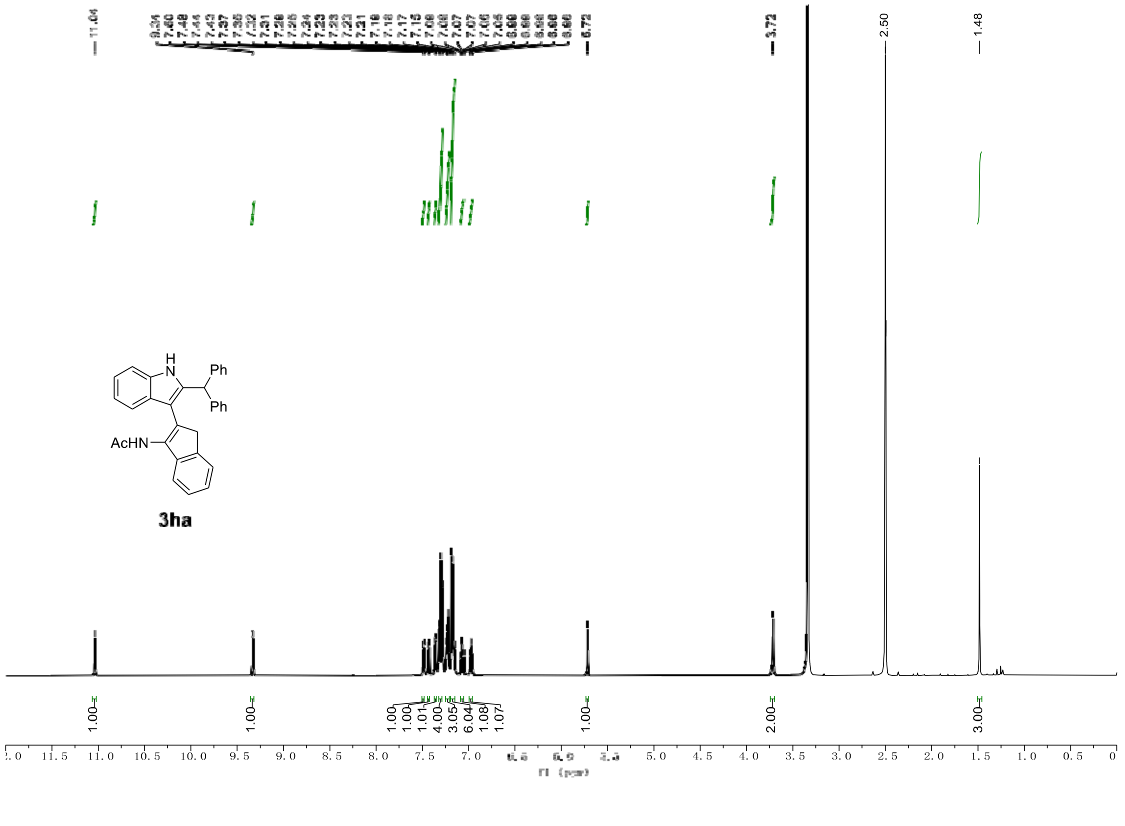

11.04
2.50
1.48
Ph
Ph
AcHN
3ha
1.00
1.00
1.00
1.01
4.00
3.05
6.04
1.08
1.07
1.00
2.00
3.00

11.5 11.0 10.5 10.0 9.5 9.0 8.5 8.0 7.5 7.0 6.5 6.0 5.5 5.0 4.5 4.0 3.5 3.0 2.5 2.0 1.5 1.0 0.5 0
f1 (ppm)

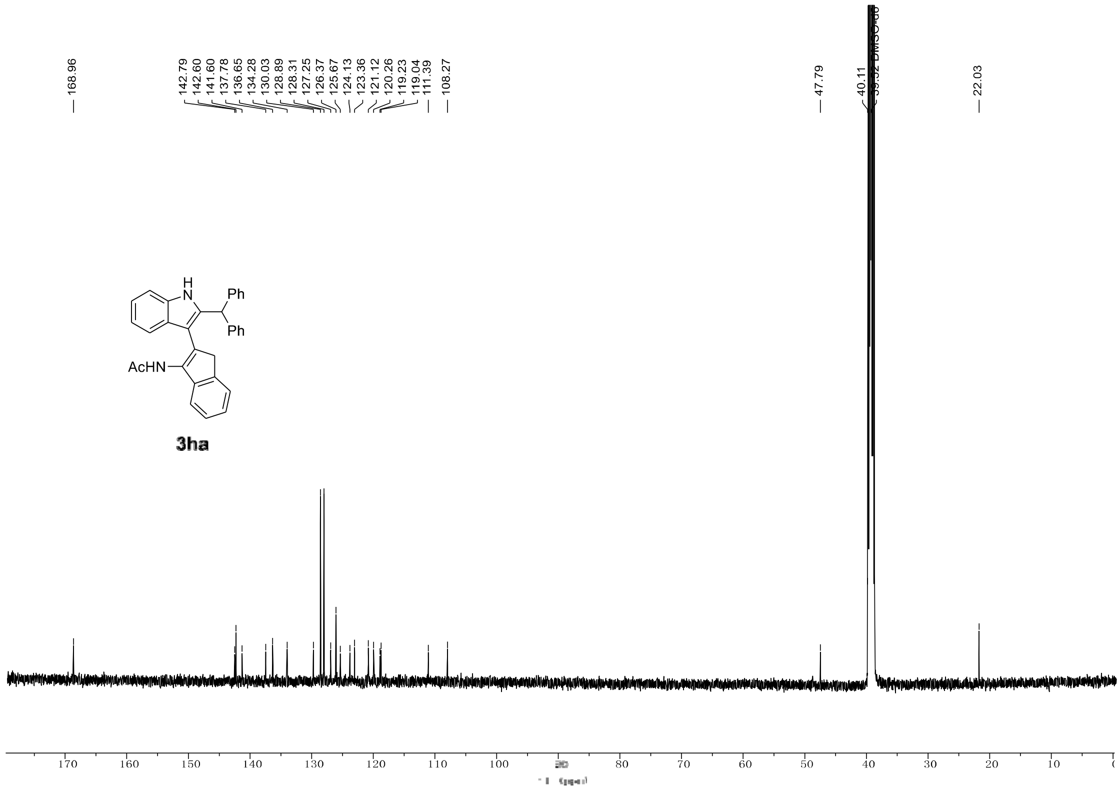
168.96
142.79
142.60
141.60
137.78
136.65
134.28
130.03
128.89
128.31
127.25
126.37
125.67
124.13
123.36
121.12
120.26
119.23
119.04
111.39
108.27
47.79
40.11
22.03
AcHN
Ph
Ph
3ha
170
160
150
140
130
120
110
100
80
70
60
50
40
30
20
10

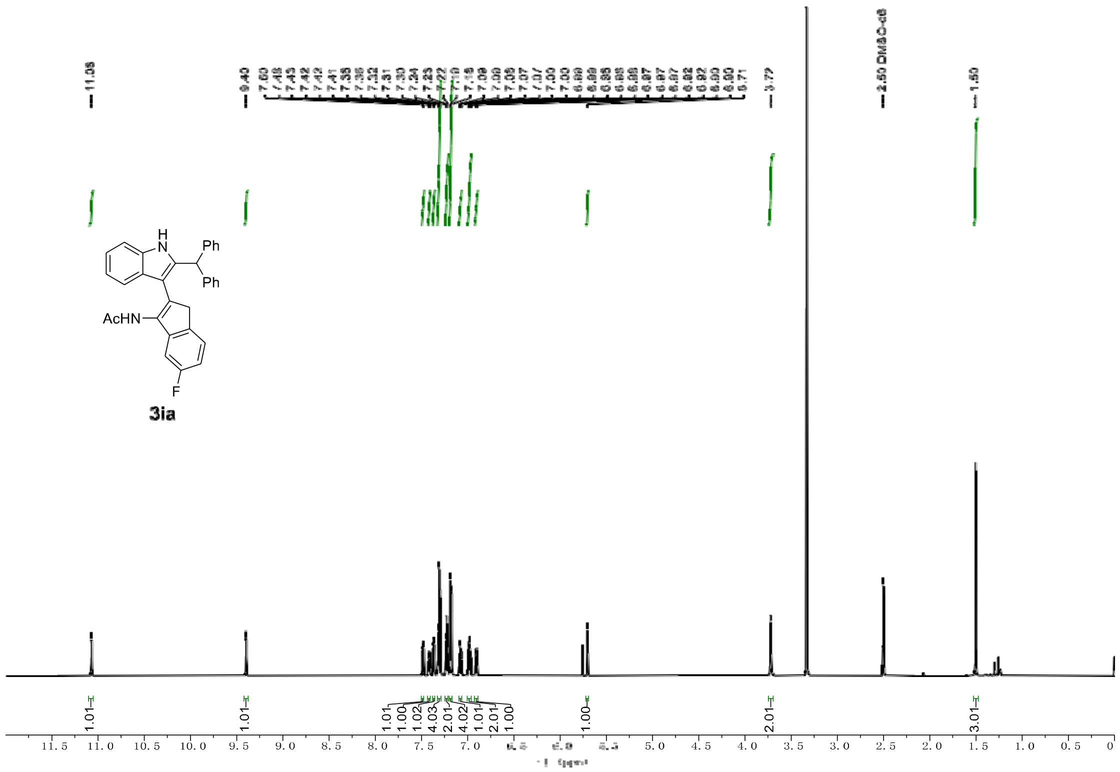
AcHN
Ph
Ph
F
3ia
1.01
1.01
1.01
1.00
1.02
4.03
2.01
4.02
1.01
2.01
1.00
1.00
2.01
3.01
11.5
11.0
10.5
10.0
9.5
9.0
8.5
8.0
7.5
7.0
5.0
4.5
4.0
3.5
3.0
2.5
2.0
1.5
1.0
0.5
0

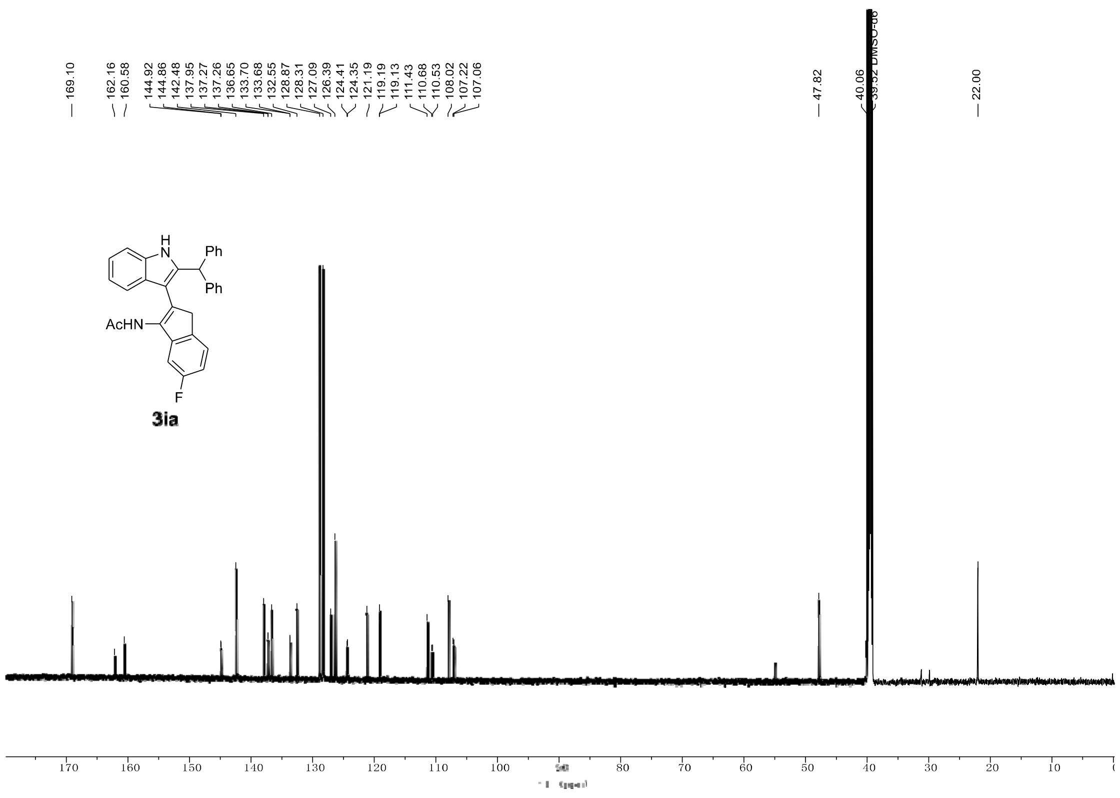
169.10
162.16
160.58
144.92
144.86
142.48
137.95
137.27
137.26
136.65
133.70
133.68
132.55
128.87
128.31
127.09
126.39
124.41
124.35
121.19
119.19
119.13
111.43
110.68
110.53
108.02
107.22
107.06
47.82
40.06
39.52 DMSO-d6
22.00
H
N
Ph
Ph
AcHN
F
3ia
170 160 150 140 130 120 110 100 80 70 60 50 40 30 20 10

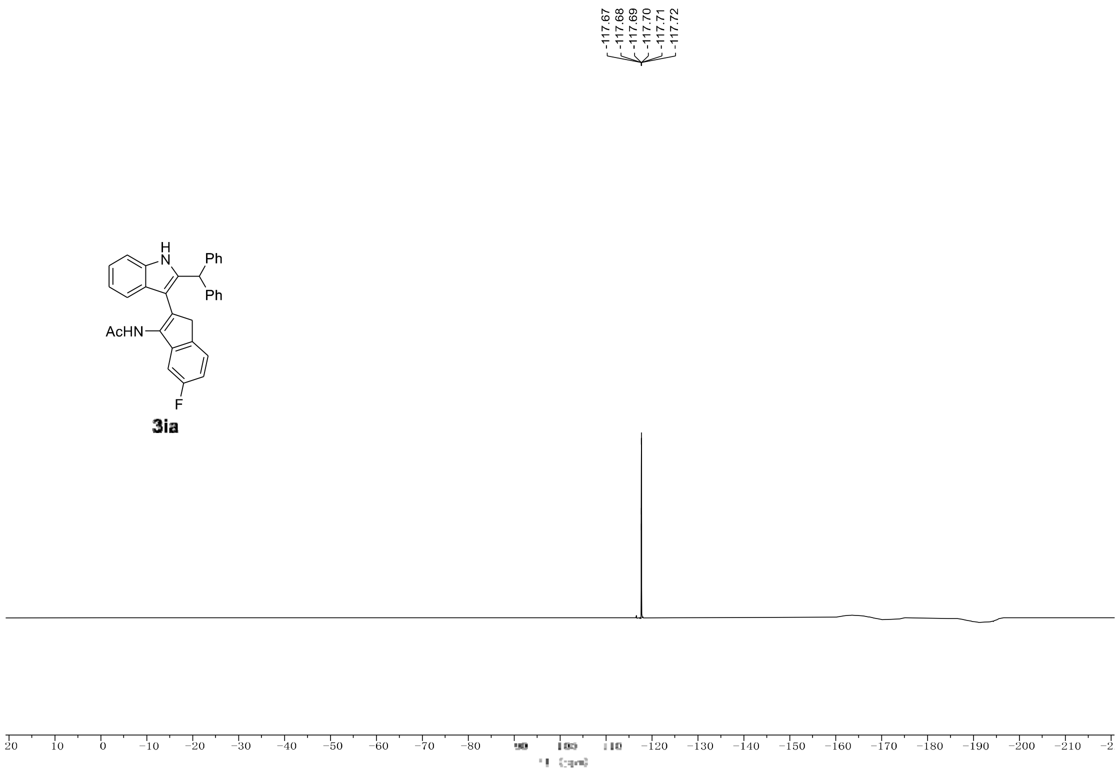
-117.67
-117.68
-117.69
-117.70
-117.71
-117.72
H
N
Ph
Ph
AcHN
F
3ia
20 10 0 −10 −20 −30 −40 −50 −60 −70 −80 −120 −130 −140 −150 −160 −170 −180 −190 −200 −210

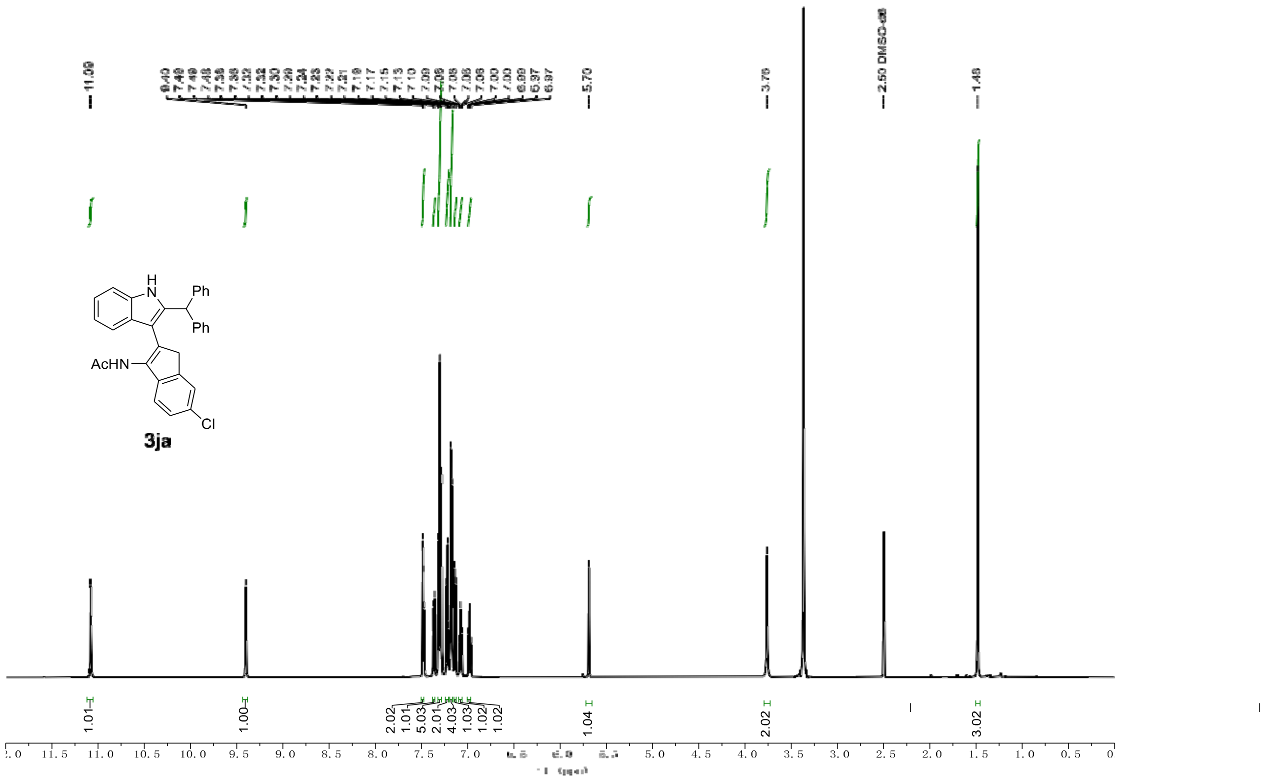

11.09
9.40
7.49
7.49
7.48
7.36
7.32
7.32
7.30
7.29
7.24
7.23
7.22
7.21
7.19
7.17
7.15
7.13
7.10
7.09
7.06
7.05
7.05
7.05
7.00
7.00
6.99
6.97
6.97
5.70
3.76
2.50 DMSO-d6
1.48
1.01
1.00
2.02
1.01
5.03
2.01
4.03
1.03
1.02
1.02
1.04
2.02
3.02
AcHN
Ph
Ph
Cl
3ja
f1 (ppm)

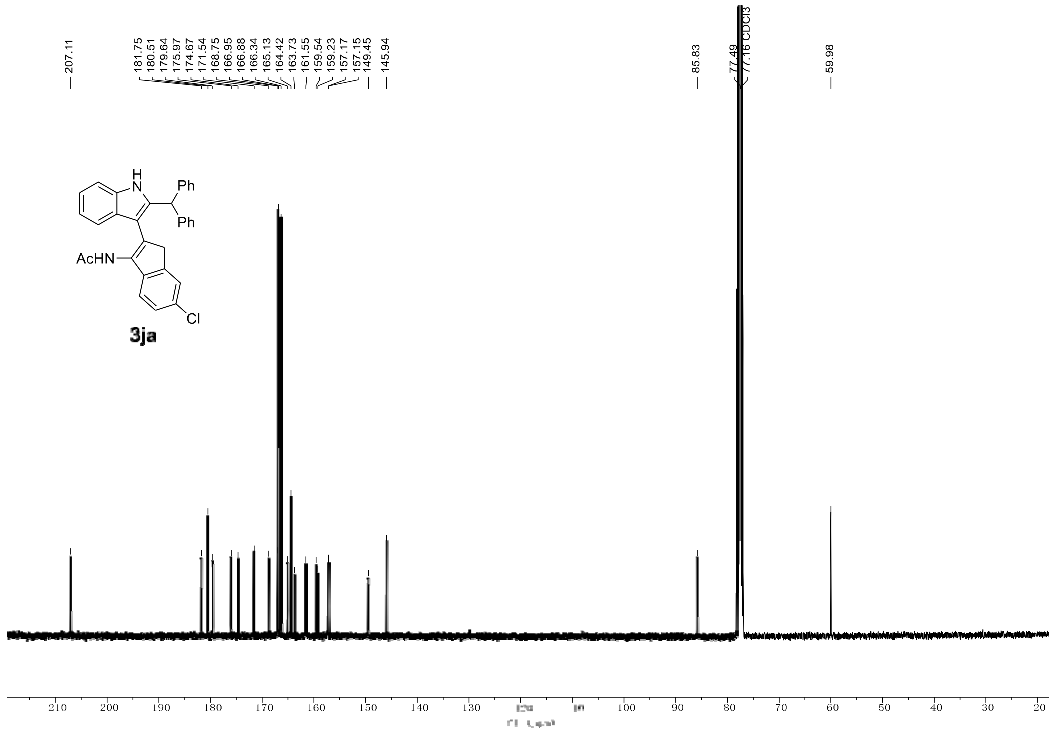

207.11
181.75
180.51
179.64
175.97
174.67
171.54
168.75
166.95
166.88
166.34
165.13
164.42
163.73
161.55
159.54
159.23
157.17
157.15
149.45
145.94
85.83
77.49
77.16 CDCl3
59.98
AcHN
Ph
Ph
Cl
3ja
f1 (ppm)

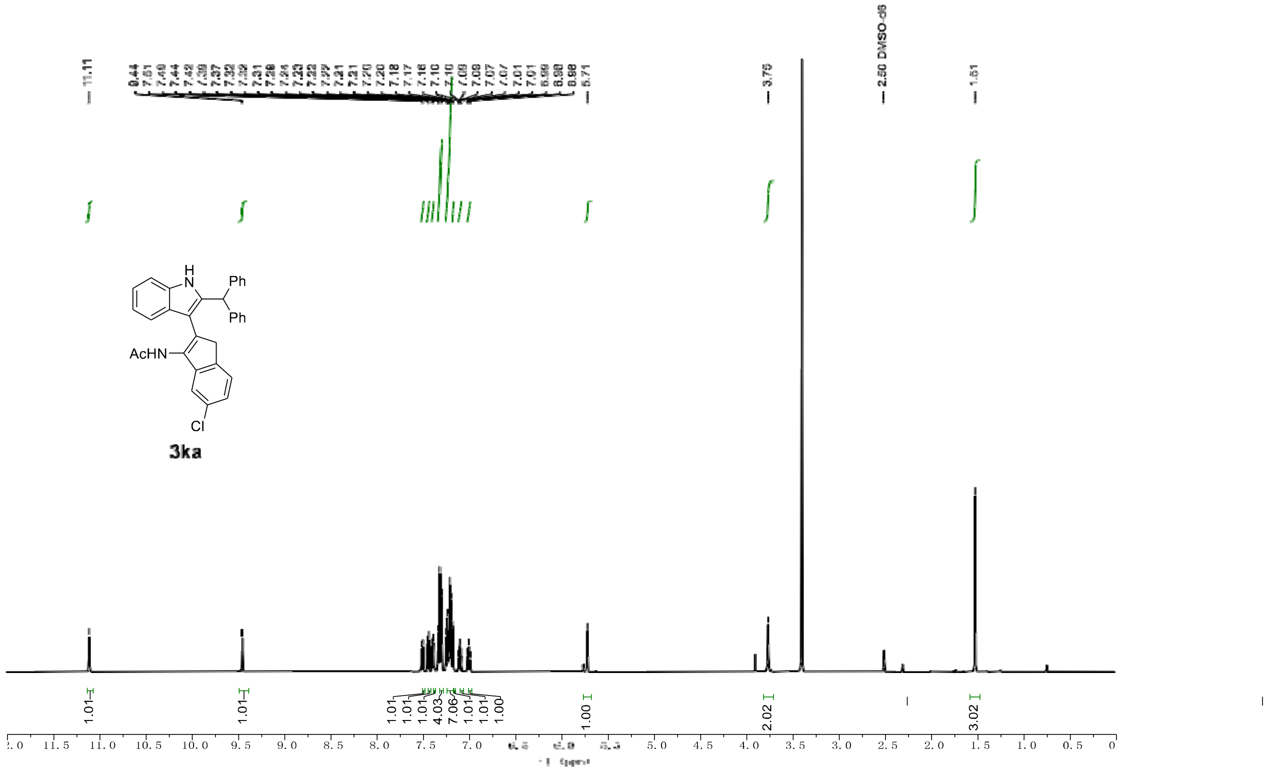
11.11
9.44
7.51
7.49
7.44
7.42
7.39
7.37
7.32
7.32
7.31
7.26
7.24
7.23
7.22
7.22
7.21
7.21
7.20
7.20
7.18
7.17
7.16
7.10
7.10
7.09
7.08
7.07
7.07
7.01
7.01
6.99
6.98
6.98
5.71
3.76
2.50 DMSO-d6
1.51
Ph
Ph
AcHN
Cl
3ka
1.01
1.01
1.01
1.01
1.01
4.03
7.06
1.01
1.01
1.00
1.00
2.02
3.02
f1 (ppm)

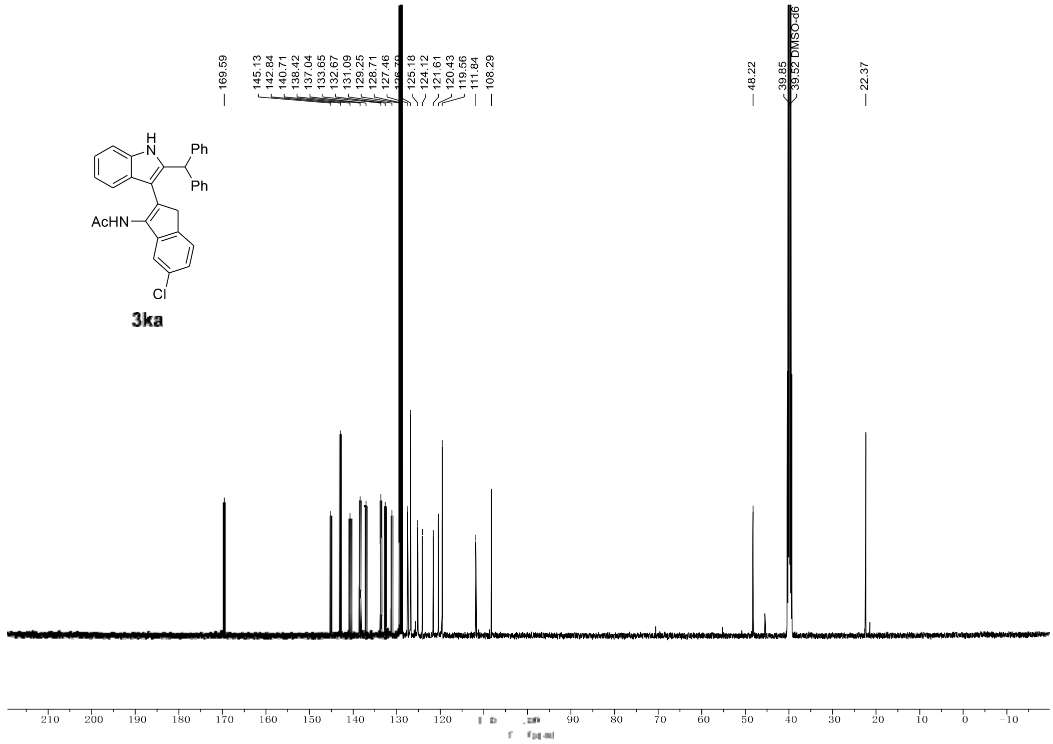
169.59
145.13
142.84
140.71
138.42
137.04
133.65
132.67
131.09
129.25
128.71
127.46
126.70
125.18
124.12
121.61
120.43
119.56
111.84
108.29
48.22
39.85
39.52 DMSO-d6
22.37
Ph
Ph
AcHN
Cl
3ka
f1 (ppm)

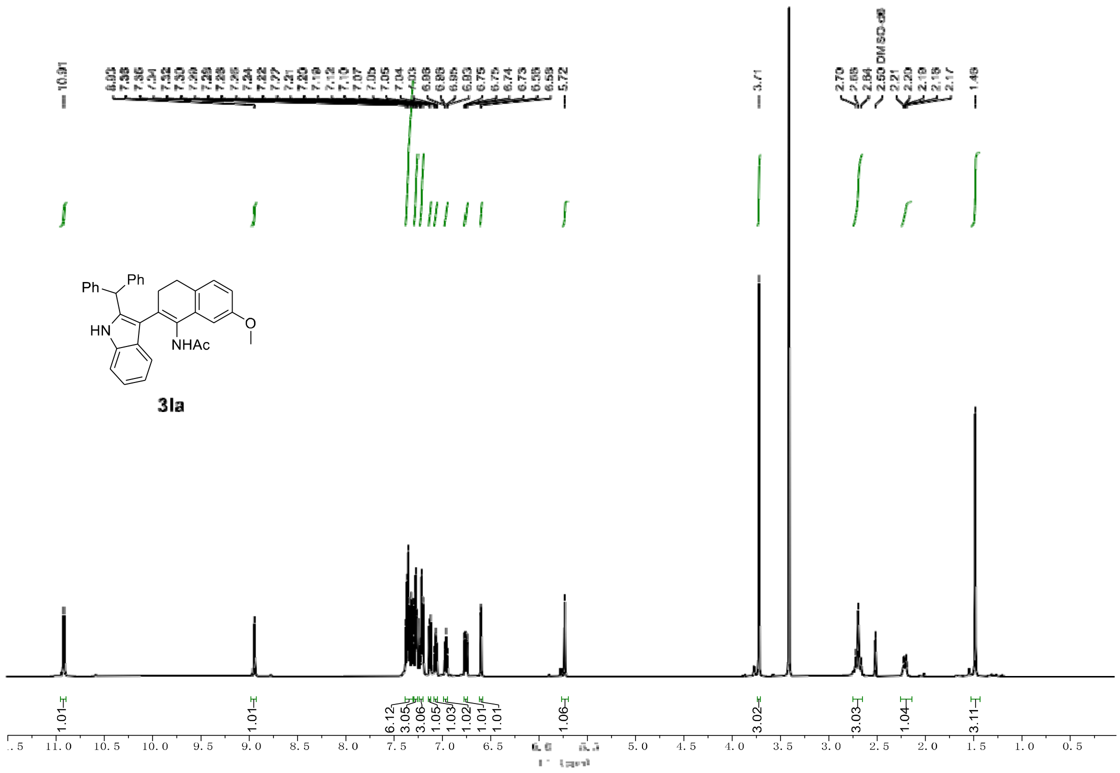

3la

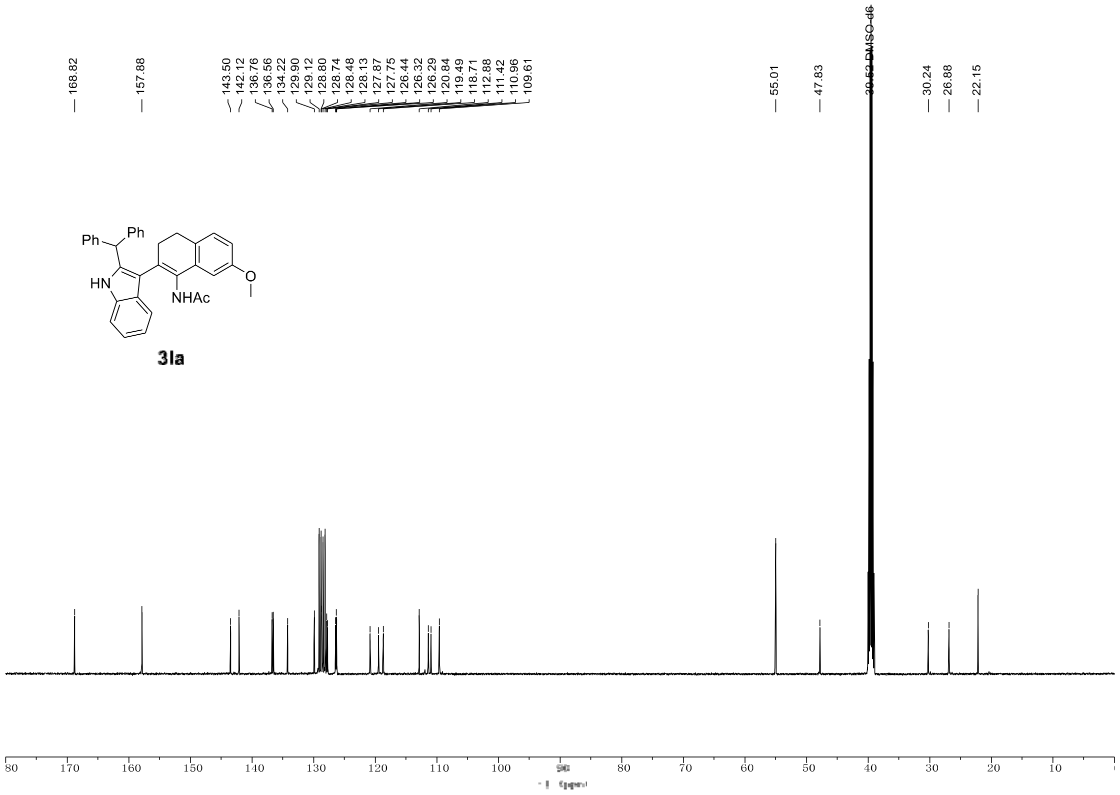

3la

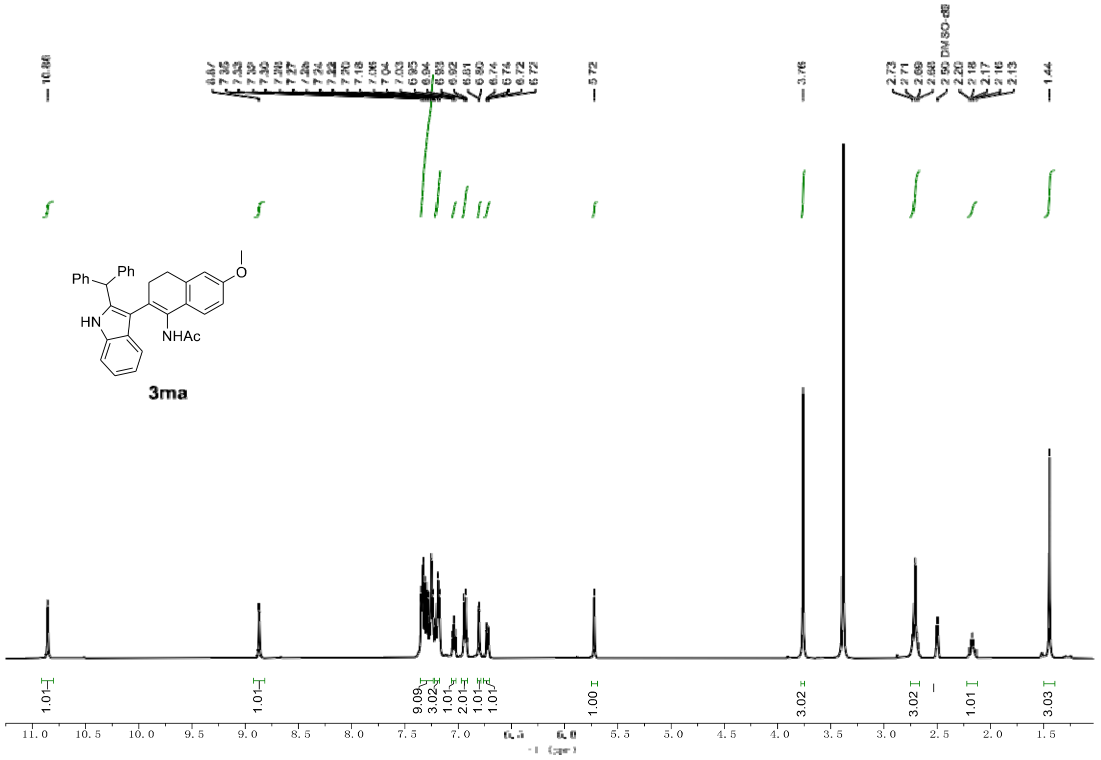

Ph
Ph
HN
NHAc
O
3ma

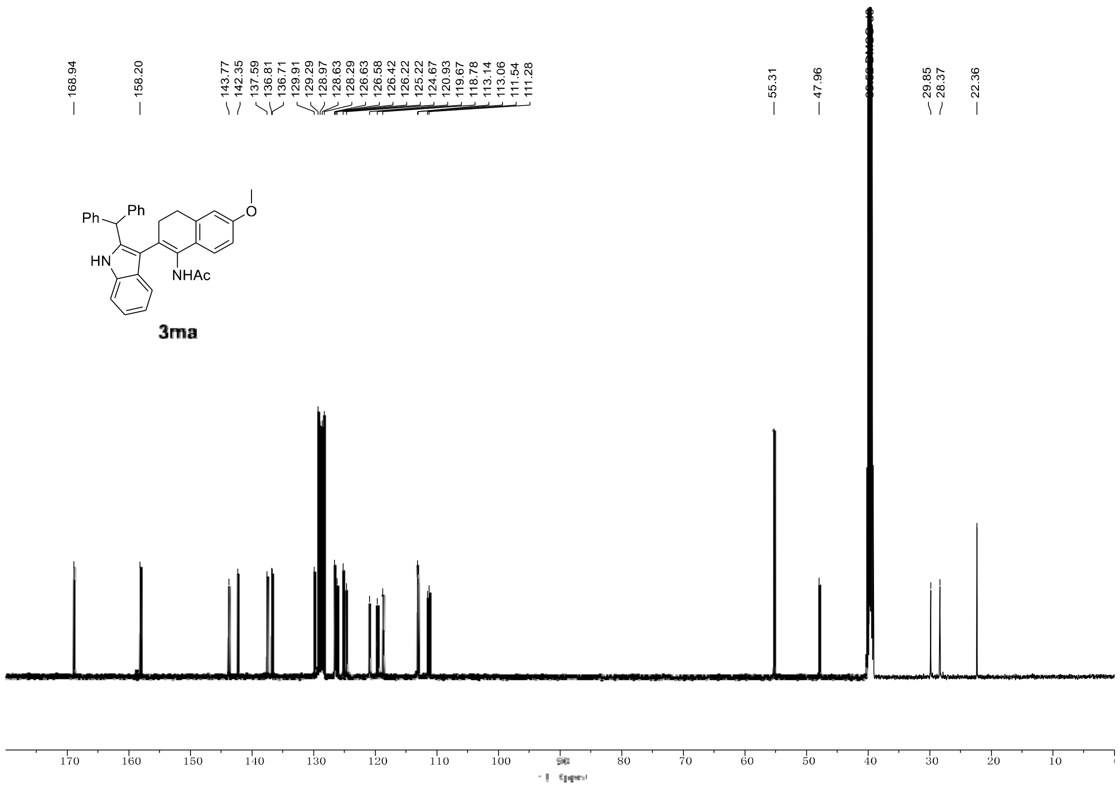

Ph
Ph
HN
NHAc
O
3ma

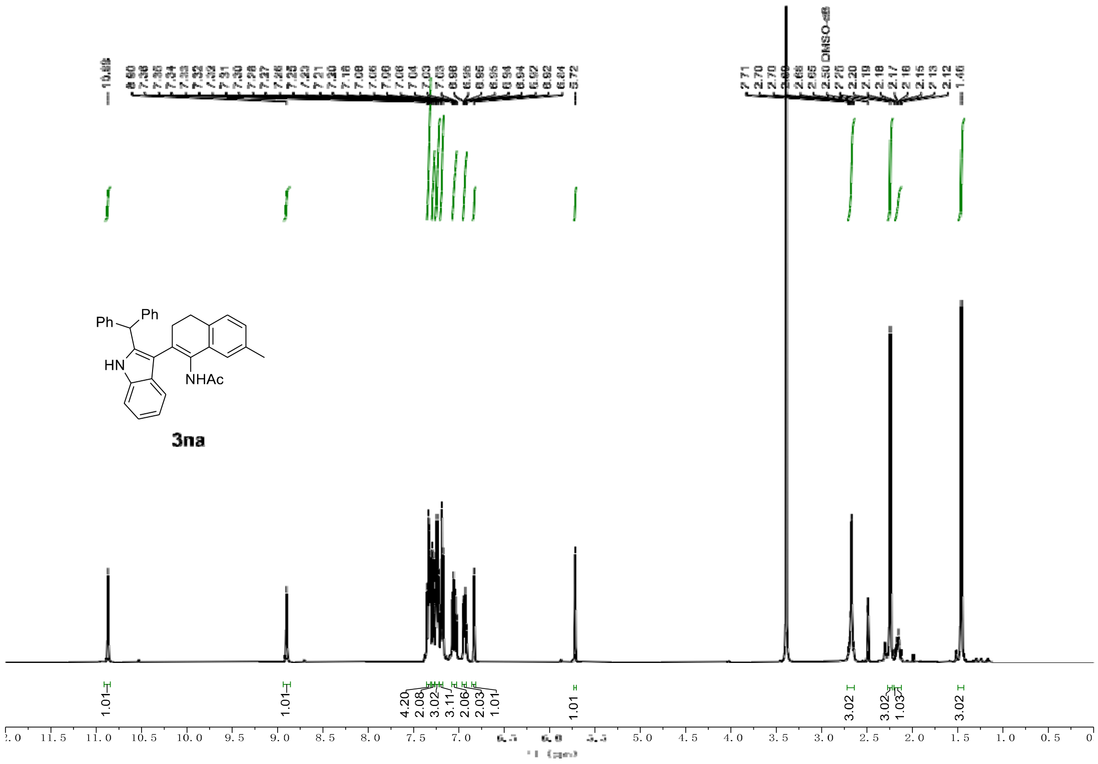

DMSO-d6
Ph
Ph
HN
NHAc
3na
1.01
1.01
4.20
2.08
3.02
3.11
2.06
2.03
1.01
1.01
3.02
3.02
1.03
3.02

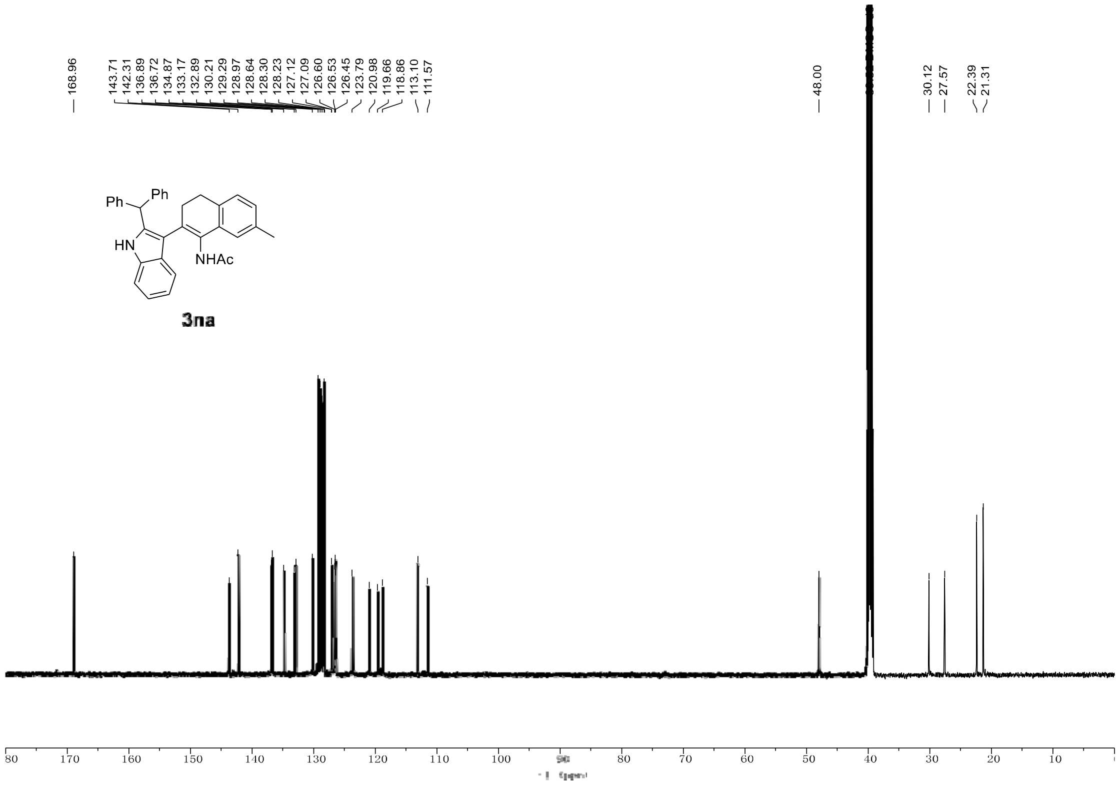

168.96
143.71
142.31
136.89
136.72
134.87
133.17
132.89
130.21
129.29
128.97
128.64
128.30
128.23
127.12
127.09
126.60
126.53
126.45
123.79
120.98
119.66
118.86
113.10
111.57
48.00
30.12
27.57
22.39
21.31
Ph
Ph
HN
NHAc
3na

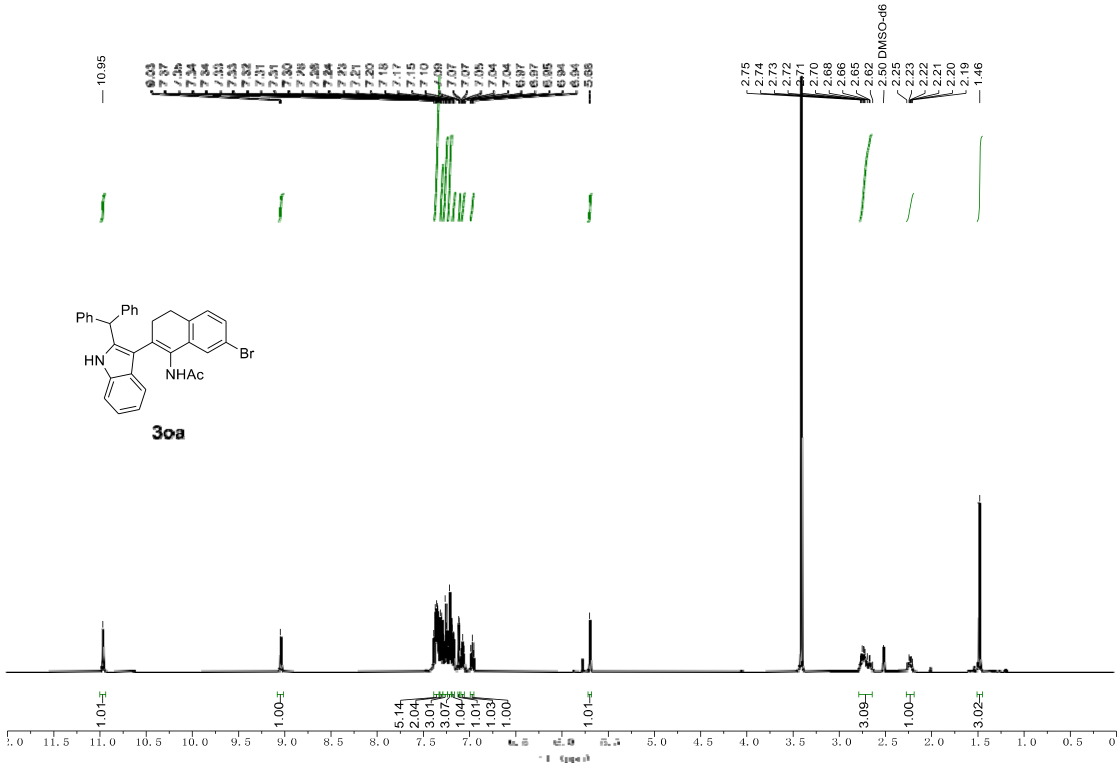
10.95
2.75
2.74
2.73
2.72
2.71
2.70
2.68
2.66
2.65
2.62
2.50 DMSO-d6
2.25
2.23
2.22
2.21
2.20
2.19
1.46
Ph
Ph
HN
NHAc
Br
3oa
1.01
1.00
5.14
2.04
3.01
3.07
1.04
1.01
1.03
1.00
1.01
3.09
1.00
3.02

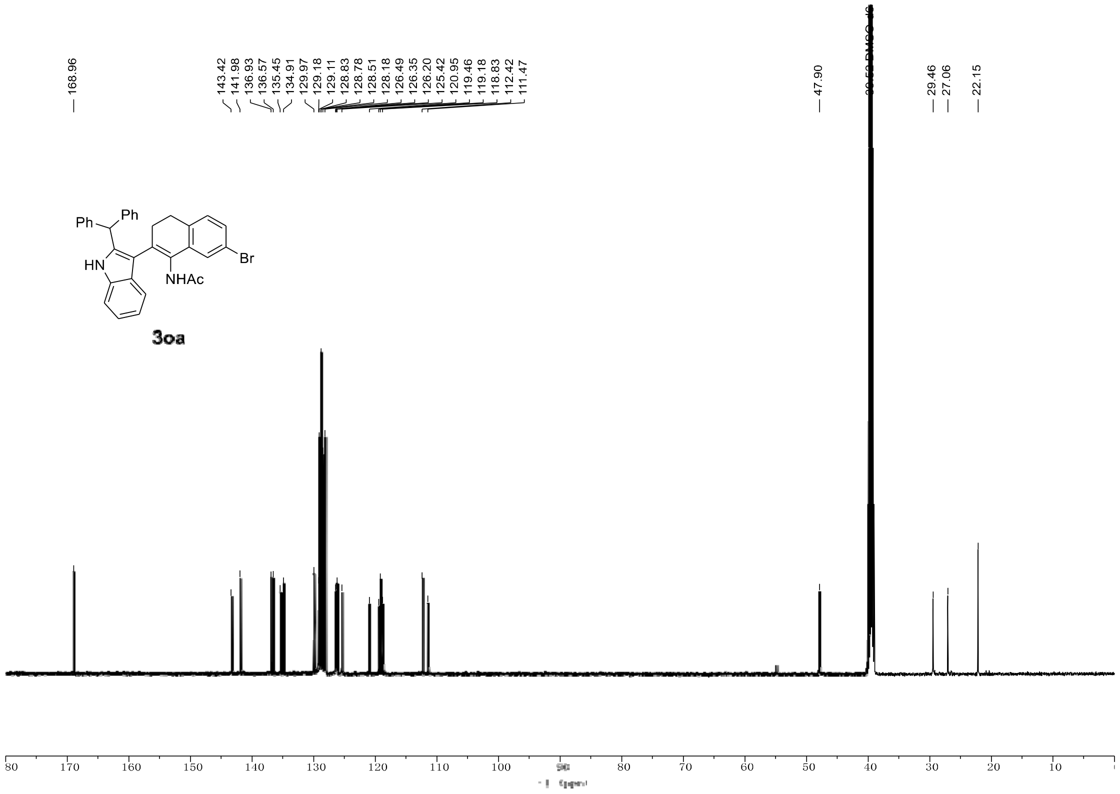
168.96
143.42
141.98
136.93
136.57
135.45
134.91
129.97
129.18
129.11
128.83
128.78
128.51
128.18
126.49
126.35
126.20
125.42
120.95
119.46
119.18
118.83
112.42
111.47
47.90
29.46
27.06
22.15
Ph
Ph
HN
NHAc
Br
3oa

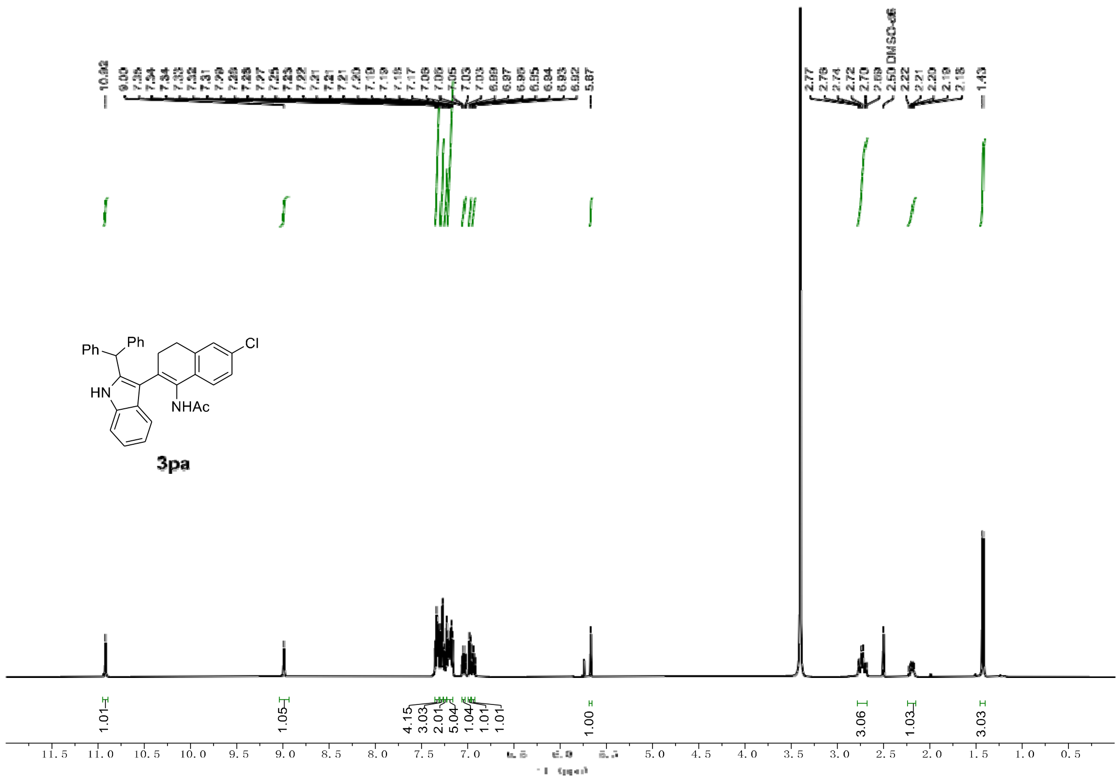

Ph
Ph
Cl
HN
NHAc
3pa
10.92
9.00
2.50 DMSO-d6
1.43
1.01
1.05
4.15
3.03
2.01
5.04
1.04
1.01
1.01
1.00
3.06
1.03
3.03
f1 (ppm)

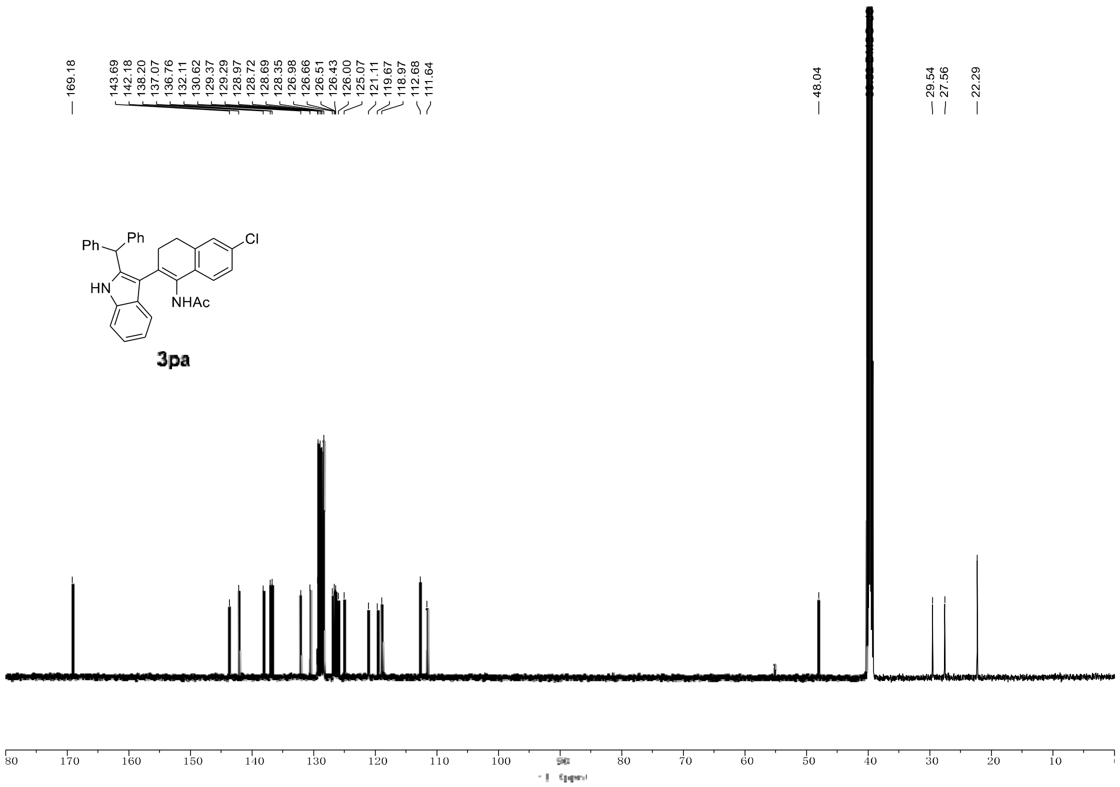

169.18
143.69
142.18
138.20
137.07
136.76
132.11
130.62
129.37
129.29
128.97
128.72
128.69
128.35
126.98
126.66
126.51
126.43
126.00
125.07
121.11
119.67
118.97
112.68
111.64
48.04
29.54
27.56
22.29
Ph
Ph
Cl
HN
NHAc
3pa
f1 (ppm)

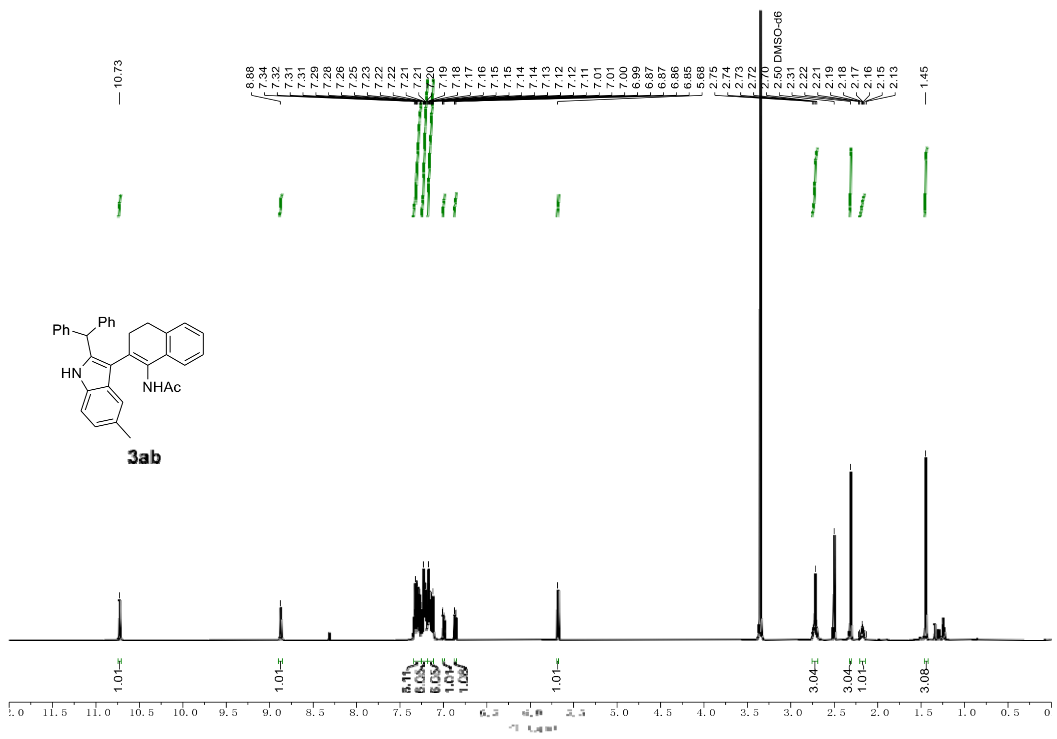

10.73
8.88 7.34 7.32 7.31 7.31 7.29 7.28 7.26 7.25 7.23 7.22 7.22 7.21 7.21 7.20 7.19 7.18 7.17 7.16 7.15 7.15 7.14 7.13 7.13 7.12 7.12 7.11 7.01 7.00 6.99 6.87 6.87 6.86 6.85 5.68
2.75 2.74 2.73 2.72 2.70 2.50 DMSO-d6 2.31 2.22 2.21 2.19 2.18 2.17 2.16 2.15 2.13
1.45
Ph
Ph
HN
NHAc
3ab
1.01
1.01
1.01
3.04
3.04
1.01
3.08

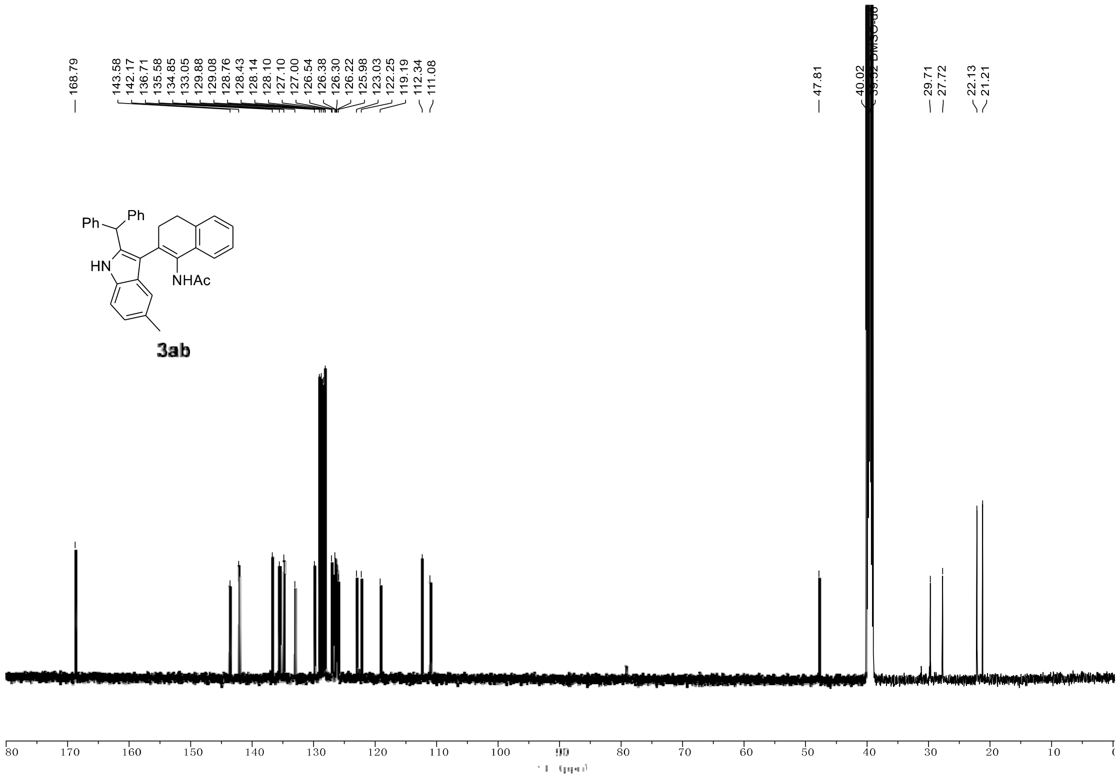

168.79
143.58 142.17 136.71 135.58 134.85 133.05 129.88 129.08 128.76 128.43 128.14 128.10 127.10 127.00 126.54 126.38 126.30 126.22 125.98 123.03 122.25 119.19 112.34 111.08
47.81
40.02
39.52 DMSO-d6
29.71 27.72
22.13 21.21
Ph
Ph
HN
NHAc
3ab

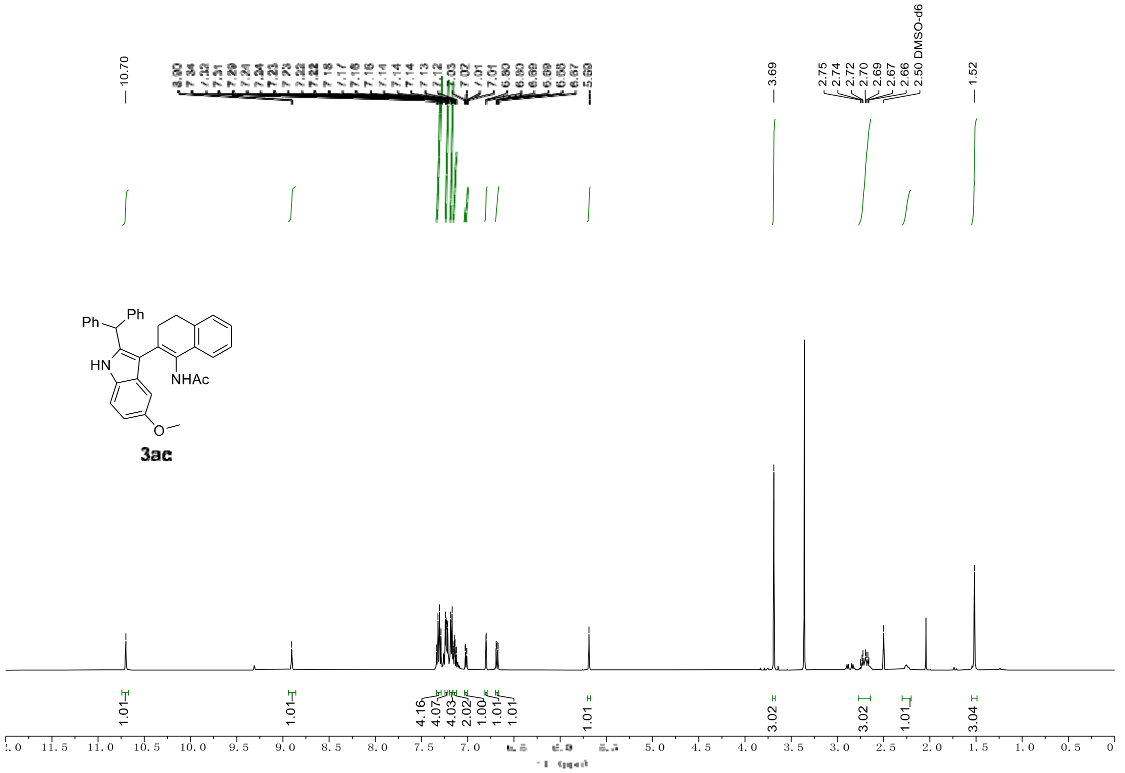
10.70
7.34
7.32
7.31
7.18
7.17
7.16
7.14
7.13
7.12
7.02
7.01
6.80
3.69
2.75
2.74
2.72
2.70
2.69
2.67
2.66
2.50 DMSO-d6
1.52
Ph
Ph
HN
NHAc
O
3ac
1.01
1.01
4.16
4.07
4.03
2.02
1.00
1.01
1.01
1.01
3.02
3.02
1.01
3.04
f1 (ppm)

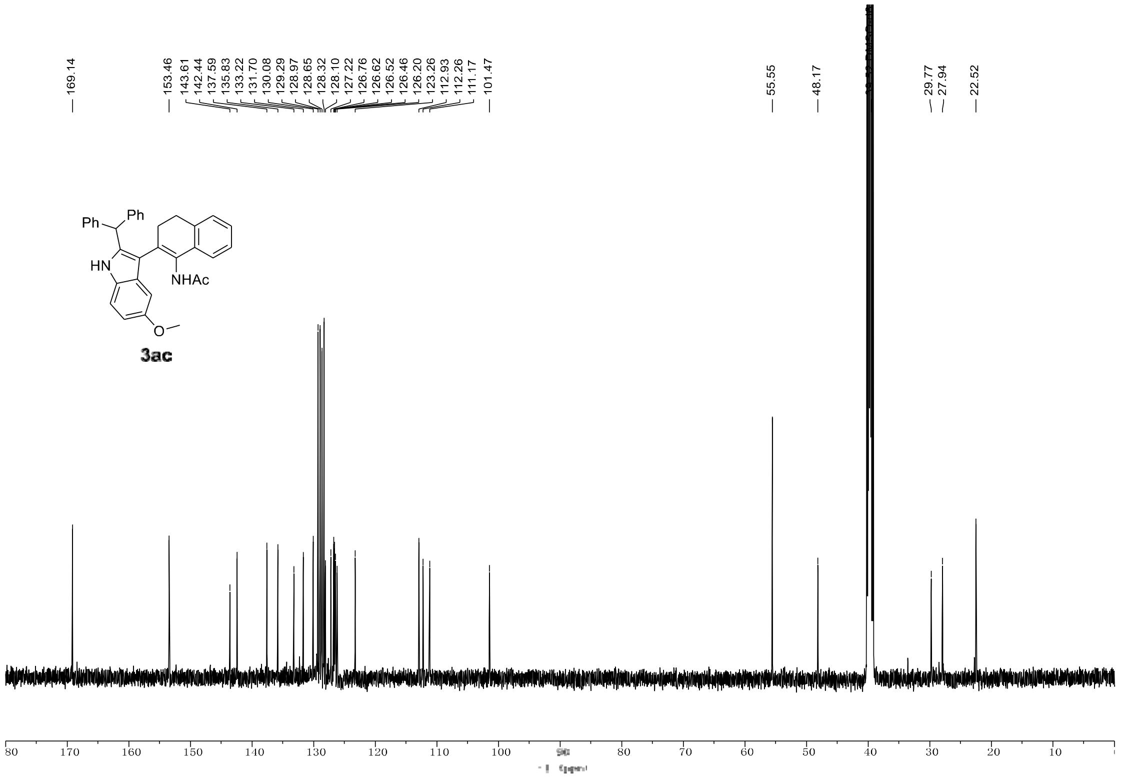
169.14
153.46
143.61
142.44
137.59
135.83
133.22
131.70
130.08
129.29
128.97
128.65
128.32
128.10
127.22
126.76
126.62
126.52
126.46
126.20
123.26
112.93
112.26
111.17
101.47
55.55
48.17
29.77
27.94
22.52
Ph
Ph
HN
NHAc
O
3ac
f1 (ppm)

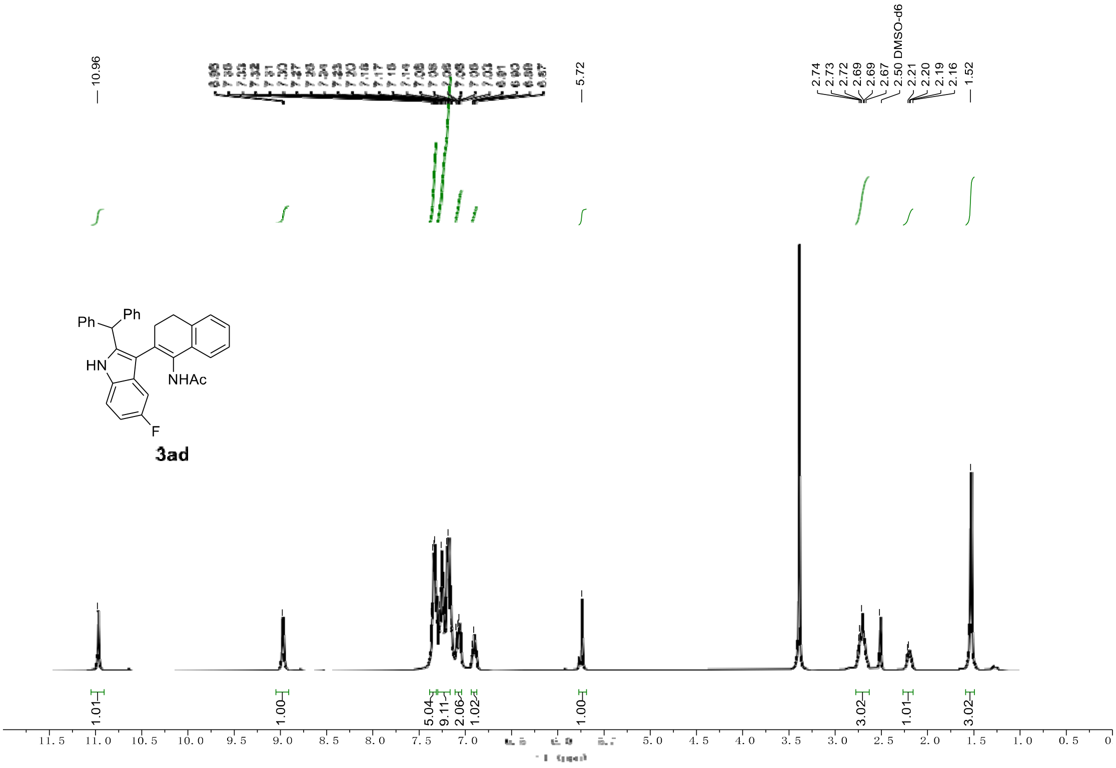

3ad

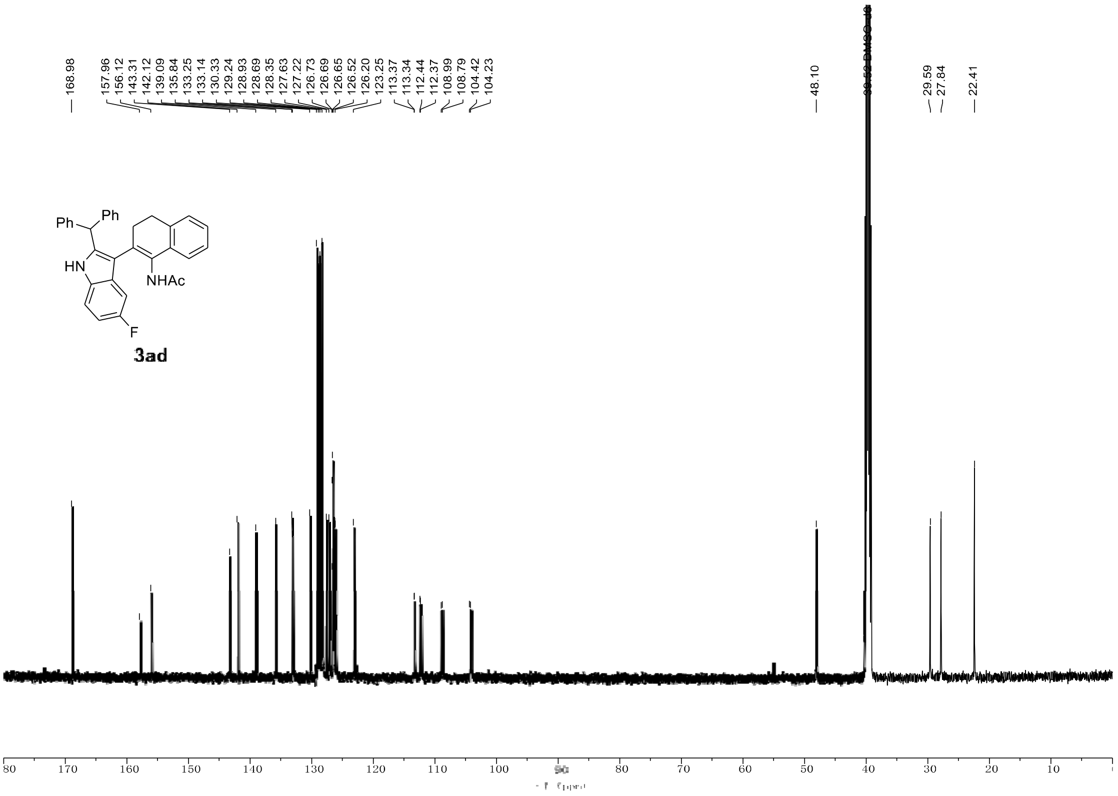

3ad

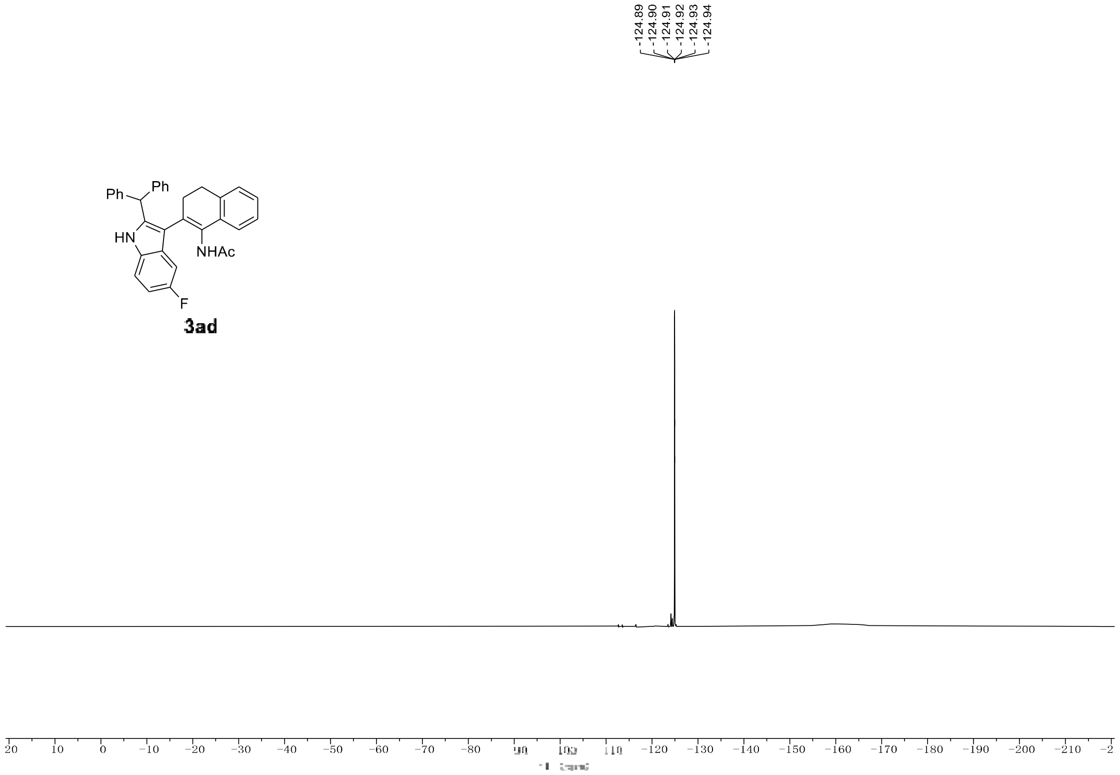
-124.89
-124.90
-124.91
-124.92
-124.93
-124.94
Ph
Ph
HN
NHAc
F
3ad
20 10 0 −10 −20 −30 −40 −50 −60 −70 −80 −90 −100 −110 −120 −130 −140 −150 −160 −170 −180 −190 −200 −210

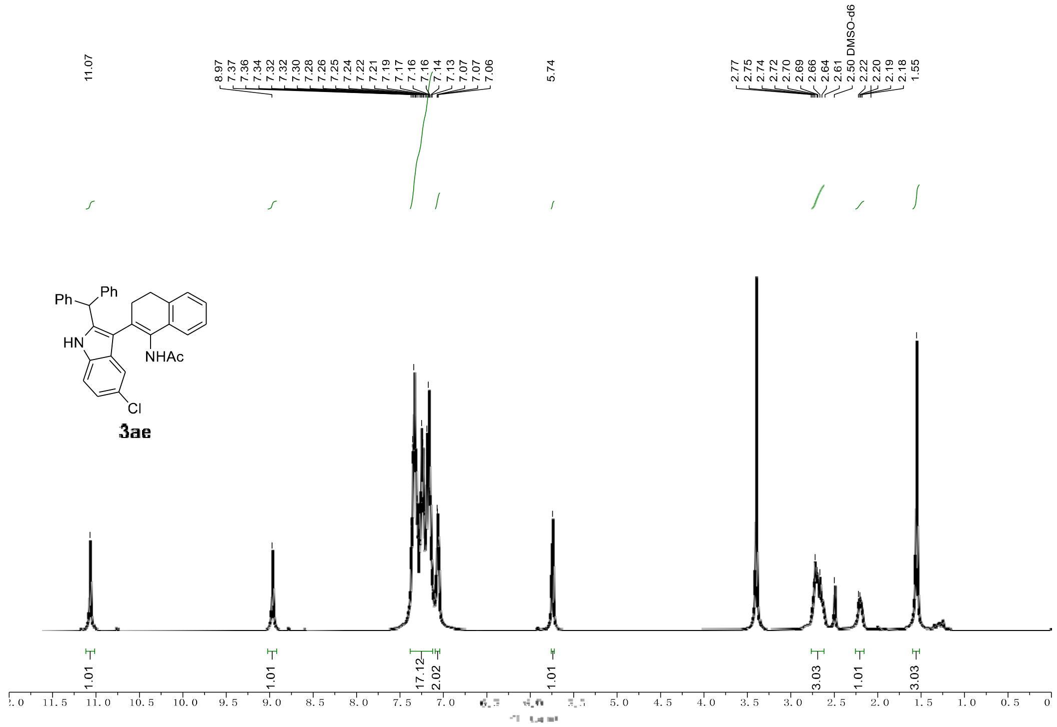
11.07
8.97
7.37
7.36
7.34
7.32
7.32
7.30
7.28
7.26
7.25
7.24
7.22
7.21
7.19
7.17
7.16
7.16
7.14
7.13
7.07
7.06
5.74
2.77
2.75
2.74
2.72
2.70
2.69
2.66
2.64
2.61
2.50 DMSO-d6
2.22
2.20
2.19
2.18
1.55
Ph
Ph
HN
NHAc
Cl
3ae
1.01
1.01
17.12
2.02
1.01
3.03
1.01
3.03
11.5 11.0 10.5 10.0 9.5 9.0 8.5 8.0 7.5 7.0 5.0 4.5 4.0 3.5 3.0 2.5 2.0 1.5 1.0 0.5 0

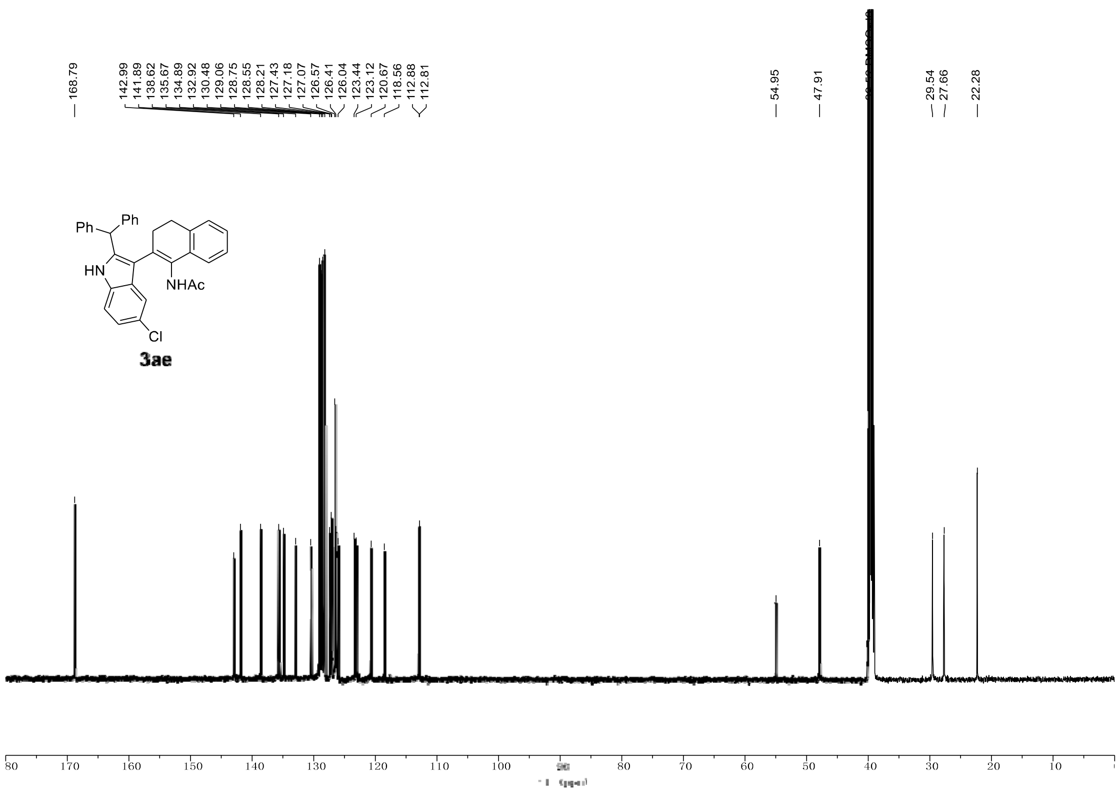
168.79
142.99
141.89
138.62
135.67
134.89
132.92
130.48
129.06
128.75
128.55
128.21
127.43
127.18
127.07
126.57
126.41
126.04
123.44
123.12
120.67
118.56
112.88
112.81
54.95
47.91
29.54
27.66
22.28
Ph
Ph
HN
NHAc
Cl
3ae

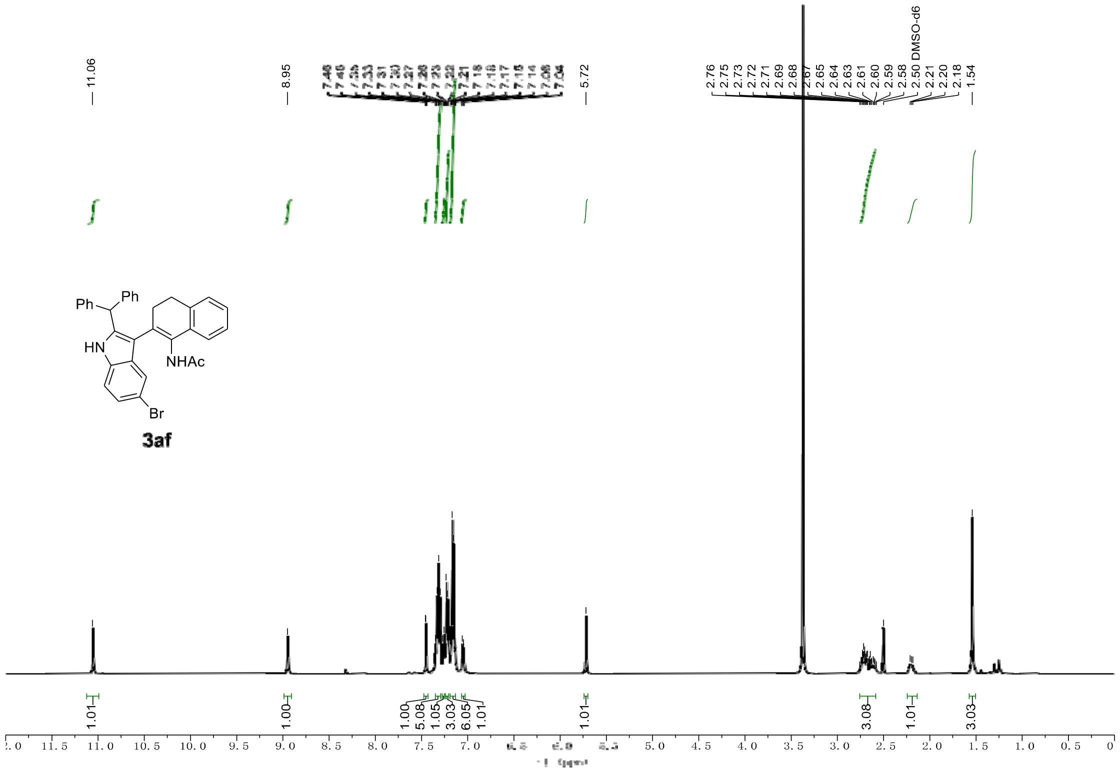
11.06
8.95
5.72
2.76
2.75
2.73
2.72
2.71
2.69
2.68
2.67
2.65
2.64
2.63
2.61
2.60
2.59
2.58
2.50 DMSO-d6
2.21
2.20
2.18
1.54
Ph
Ph
HN
NHAc
Br
3af
1.01
1.00
1.00
5.08
1.05
3.03
6.05
1.01
1.01
3.08
1.01
3.03

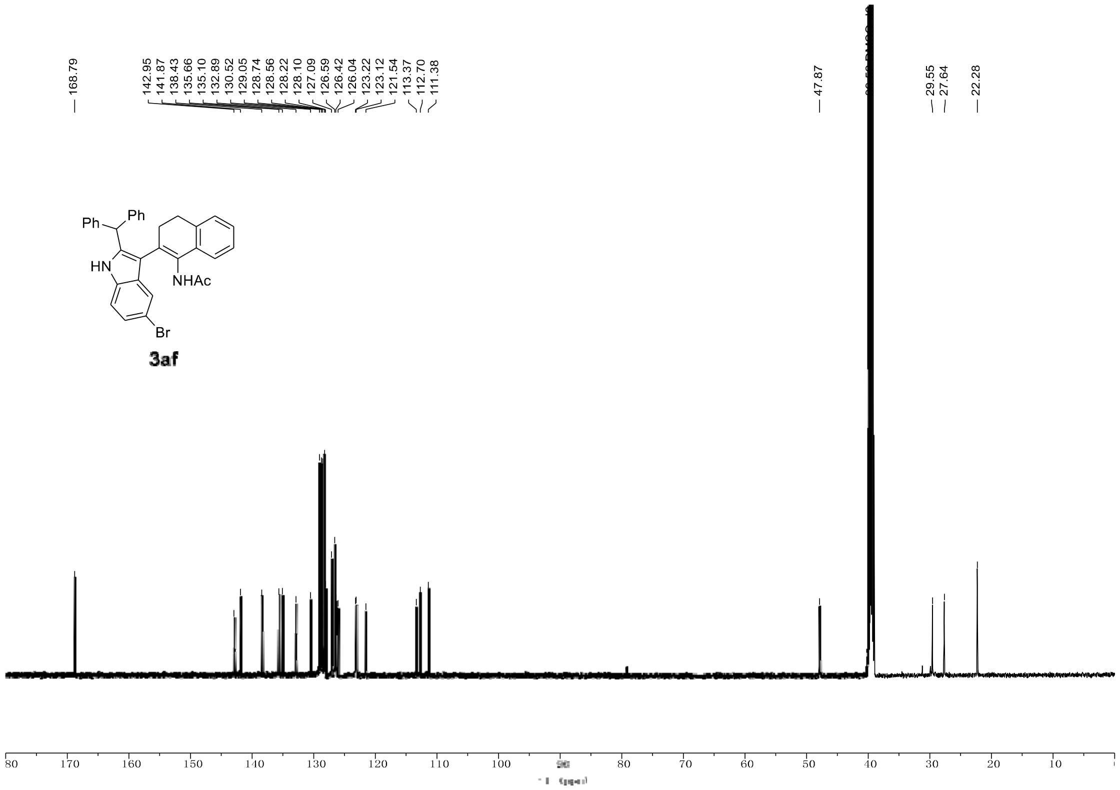

168.79
142.95
141.87
138.43
135.66
135.10
132.89
130.52
129.05
128.74
128.56
128.22
128.10
127.09
126.59
126.42
126.04
123.22
123.12
121.54
113.37
112.70
111.38
47.87
29.55
27.64
22.28
Ph
Ph
HN
NHAc
Br

3af

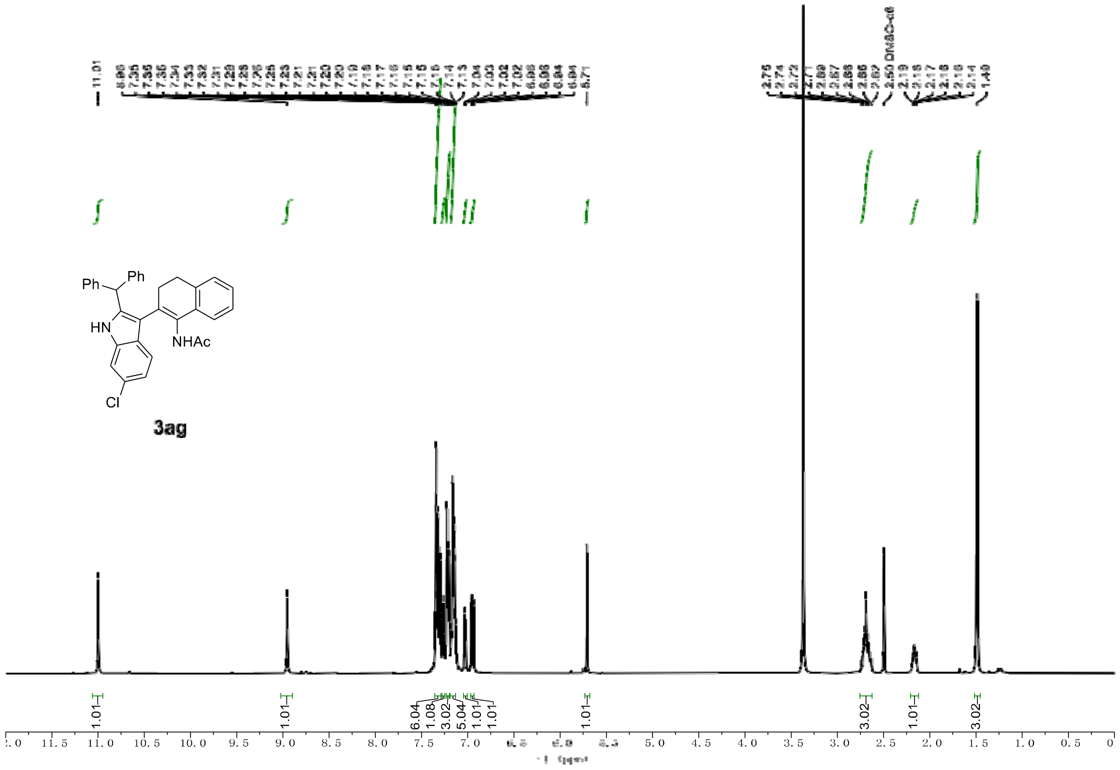

11.01
Ph
Ph
HN
NHAc
Cl
1.01
1.01
6.04
1.08
3.02
5.04
1.01
1.01
1.01
3.02
1.01
3.02

3ag

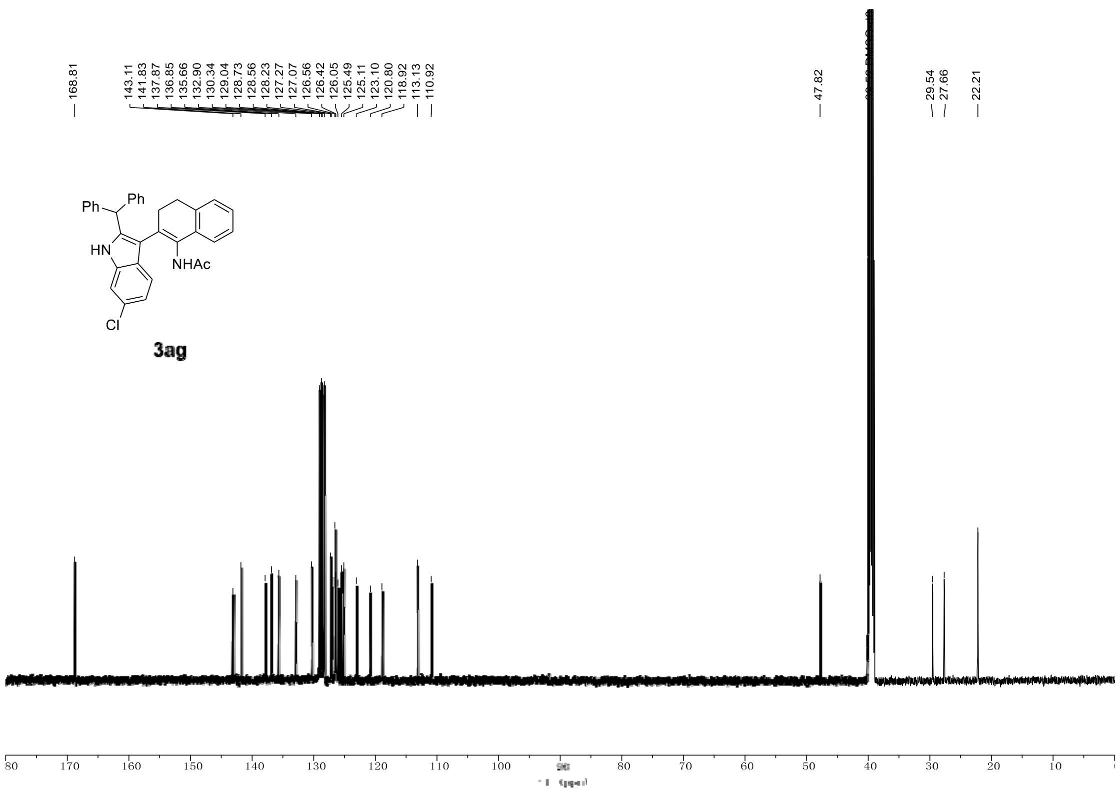
168.81
143.11
141.83
137.87
136.85
135.66
132.90
130.34
129.04
128.73
128.56
128.23
127.27
127.07
126.56
126.42
126.05
125.49
125.11
123.10
120.80
118.92
113.13
110.92
47.82
29.54
27.66
22.21
Ph
Ph
HN
NHAc
Cl
3ag

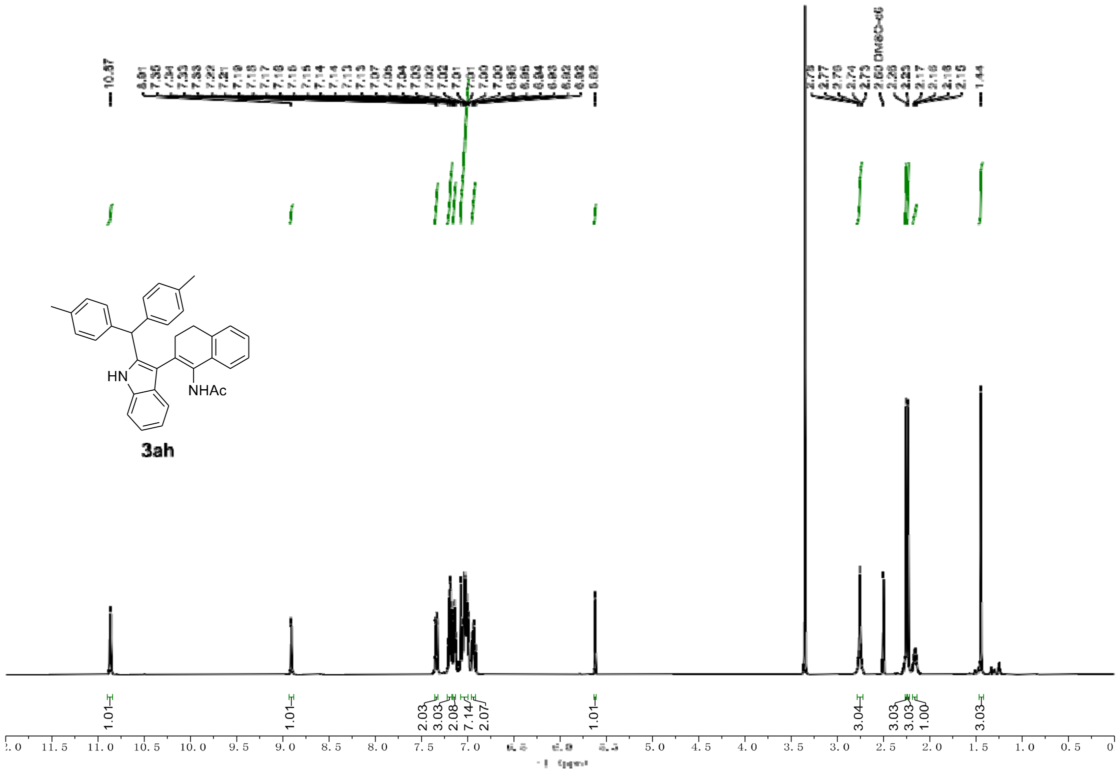
HN
NHAc
3ah
1.01
1.01
2.03
3.03
2.08
7.14
2.07
1.01
3.04
3.03
3.03
1.00
3.03

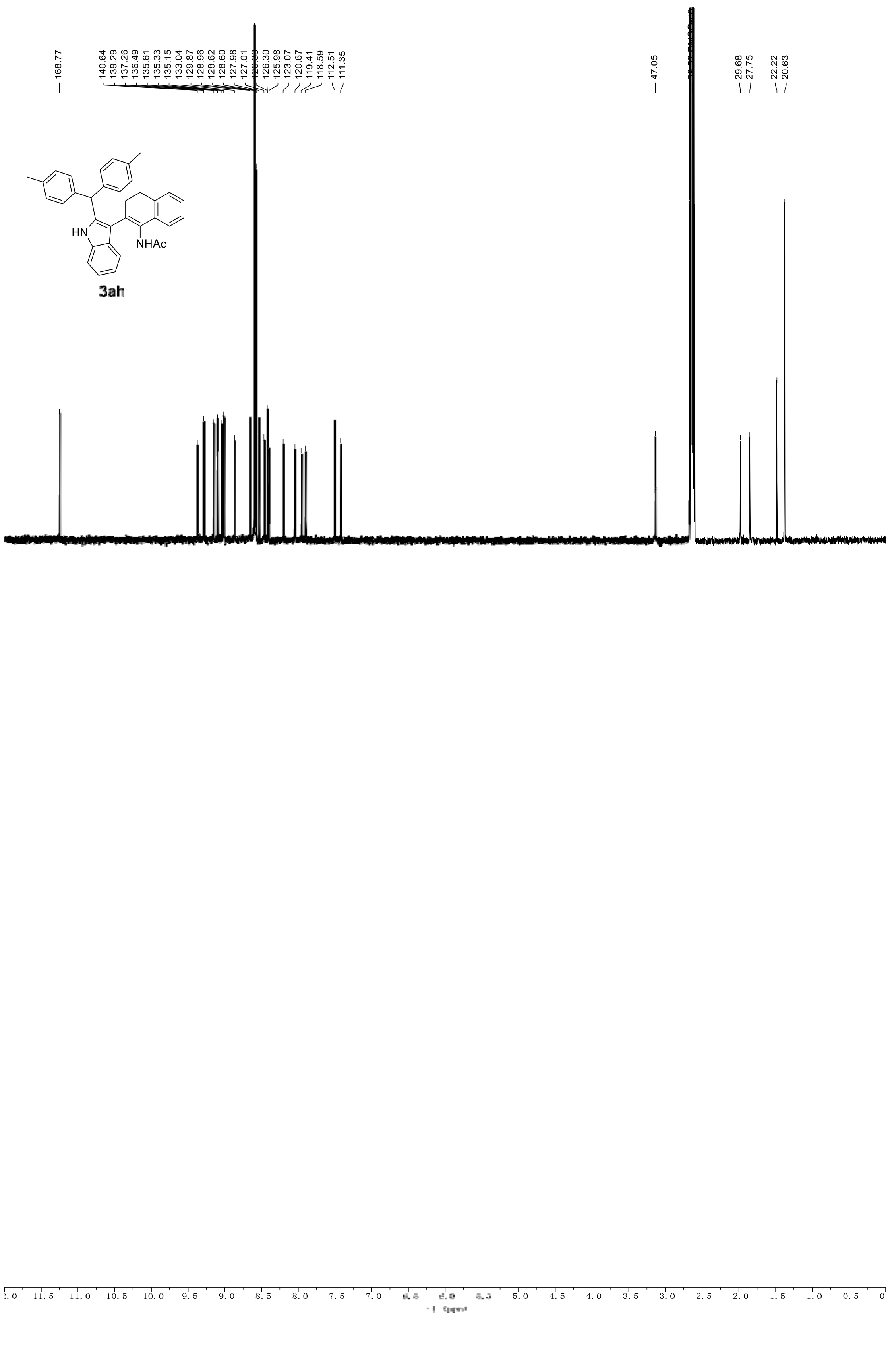
168.77
140.64
139.29
137.26
136.49
135.61
135.33
135.15
133.04
129.87
128.96
128.62
128.60
127.98
127.01
126.30
125.98
123.07
120.67
119.41
118.59
112.51
111.35
47.05
29.68
27.75
22.22
20.63
HN
NHAc
3ah
11.5 11.0 10.5 10.0 9.5 9.0 8.5 8.0 7.5 7.0 5.0 4.5 4.0 3.5 3.0 2.5 2.0 1.5 1.0 0.5 0
